# Global mercury concentrations in biota: their use as a basis for a global biomonitoring framework

**DOI:** 10.1007/s10646-024-02747-x

**Published:** 2024-04-29

**Authors:** David C. Evers, Joshua T. Ackerman, Staffan Åkerblom, Dominique Bally, Nil Basu, Kevin Bishop, Nathalie Bodin, Hans Fredrik Veiteberg Braaten, Mark E. H. Burton, Paco Bustamante, Celia Chen, John Chételat, Linroy Christian, Rune Dietz, Paul Drevnick, Collin Eagles-Smith, Luis E. Fernandez, Neil Hammerschlag, Mireille Harmelin-Vivien, Agustin Harte, Eva M. Krümmel, José Lailson Brito, Gabriela Medina, Cesar Augusto Barrios Rodriguez, Iain Stenhouse, Elsie Sunderland, Akinori Takeuchi, Tim Tear, Claudia Vega, Simon Wilson, Pianpian Wu

**Affiliations:** 1 Biodiversity Research Institute, 276 Canco Road, Portland, ME 04103 USA; 2 U.S. Geological Survey, Western Ecological Research Center, Dixon Field Station, 800 Business Park Drive, Suite D, Dixon, CA 95620 USA; 3 Swedish University of Agricultural Sciences, Uppsala, Sweden; 4 African Center for Environmental Health, BP 826 Cidex 03, Abidjan, Côte d’Ivoire; 5 Faculty of Agricultural and Environmental Sciences, McGill University, Montreal, QC Canada; 6 Department of Aquatic Sciences and Assessment, Swedish University of Agricultural Sciences, Upsalla, Sweden; 7 Research Institute for Sustainable Development Seychelles Fishing Authority, Victoria, Seychelles; 8 Norwegian Institute for Water Research, Oslo, Norway; 9 Littoral, Environnement et Sociétés (LIENSs), UMR 7266 CNRS La Rochelle Université, 2 Rue Olympe de Gouges, 17000 La Rochelle, France; 10 Department of Biological Sciences, Dartmouth College, Hanover, NH 03755 USA; 11 Environment and Cliamte Change Canada, National Wildlife Research Centre, Ottawa, ON K1S 5B6 Canada; 12 Department of Analytical Services, Dunbars, Friars Hill, St John, Antigua and Barbuda; 13 Department of Ecoscience, Aarhus University, Arctic Research Centre (ARC), Department of Ecoscience, P.O. Box 358, DK-4000 Roskilde, Denmark; 14 Teck American Incorporated, 2500 University Drive NW, Calgary, AB T2N 1N4 Canada; 15 U.S. Geological Survey, Forest and Rangeland Ecosystem Science Center, 3200 SW Jefferson Way, Corvallis, OR 97331 USA; 16 Sabin Center for Environment and Sustainability and Department of Biology, Wake Forest University, Winston-Salem, NC 29106 USA; 17 Centro de Innovación Científica Amazonica (CINCIA), Puerto Maldonado, Madre de Dios Peru; 18 Shark Research Foundation Inc, 29 Wideview Lane, Boutiliers Point, NS B3Z 0M9 Canada; 19 Aix-Marseille Université, Université de Toulon, CNRS/INSU/IRD, Institut Méditerranéen d’Océanologie (MIO), UM 110, Campus de Luminy, case 901, 13288 Marseille, cedex 09 France; 20 Basel, Rotterdam and Stockholm Conventions Secretariat, United Nations Environment Programme (UNEP), Chem. des Anémones 15, 1219 Vernier Geneva, Switzerland; 21 Inuit Circumpolar Council-Canada, Ottawa, Canada and ScienTissiME Inc, Barry’s Bay, ON Canada; 22 Universidade do Estado do Rio de Janeiro, Rua Sao Francisco Xavier, 524, Sala 4002, CEP 20550-013, Maracana, Rio de Janeiro, RJ Brazil; 23 Director of Basel Convention Coordinating Centre, Stockholm Convention Regional Centre for Latin America and the Caribbean, Hosted by the Ministry of Environment, Montevideo, Uruguay; 24 Tropical Marine Sciences - Laboratory of Coastal Biogeochemistry, University of Panamá, Panamá City, Panama; 25 Harvard University, Pierce Hall 127, 29 Oxford Street, Cambridge, MA 02138 USA; 26 National Institute for Environmental Studies, Health and Environmental Risk Division, 16-2 Onogawa Tsukuba, Ibaraki, 305-8506 Japan; 27 Centro de Innovaccion Cientifica Amazonica (CINCIA), Jiron Ucayali 750, Puerto Maldonado, Madre de Dios 17001 Peru; 28 Arctic Monitoring and Assessment Programme (AMAP) Secretariat, N-9296 Tromsø, Norway

**Keywords:** Bioaccumulation, Biomagnification, Geographical trends, Mercury, Minamata Convention, Temporal trends

## Abstract

An important provision of the Minamata Convention on Mercury is to monitor and evaluate the effectiveness of the adopted measures and its implementation. Here, we describe for the first time currently available biotic mercury (Hg) data on a global scale to improve the understanding of global efforts to reduce the impact of Hg pollution on people and the environment. Data from the peer-reviewed literature were compiled in the Global Biotic Mercury Synthesis (GBMS) database (>550,000 data points). These data provide a foundation for establishing a biomonitoring framework needed to track Hg concentrations in biota globally. We describe Hg exposure in the taxa identified by the Minamata Convention: fish, sea turtles, birds, and marine mammals. Based on the GBMS database, Hg concentrations are presented at relevant geographic scales for continents and oceanic basins. We identify some effective regional templates for monitoring methylmercury (MeHg) availability in the environment, but overall illustrate that there is a general lack of regional biomonitoring initiatives around the world, especially in Africa, Australia, Indo-Pacific, Middle East, and South Atlantic and Pacific Oceans. Temporal trend data for Hg in biota are generally limited. Ecologically sensitive sites (where biota have above average MeHg tissue concentrations) have been identified throughout the world. Efforts to model and quantify ecosystem sensitivity locally, regionally, and globally could help establish effective and efficient biomonitoring programs. We present a framework for a global Hg biomonitoring network that includes a three-step continental and oceanic approach to integrate existing biomonitoring efforts and prioritize filling regional data gaps linked with key Hg sources. We describe a standardized approach that builds on an evidence-based evaluation to assess the Minamata Convention’s progress to reduce the impact of global Hg pollution on people and the environment.

## Introduction

Global recognition of mercury (Hg) as an environmental contaminant with effects on humans and wildlife has resulted in the global establishment of a multilateral environmental agreement - the Minamata Convention on Mercury (Bank 2020). To date over 145 countries have ratified the Convention, agreeing to reduce anthropogenic sources of Hg to the environment and to evaluate if the established provisions are effective in meeting its goals. One important provision of the Convention is to monitor and evaluate the effectiveness of its implementation (Articles 1 and 22). This will require having standardized measurements of environmental concentrations of Hg in abiotic and biotic compartments over time (Evers et al. 2016). The establishment of “baseline” levels is critical for determining whether Hg levels have declined in response to source reductions mandated by the Convention, a monitoring principle that is required in other disciplines (e.g., Verra 2021). In order to track Hg concentrations over time, it will be necessary to select the environmental abiotic matrices (e.g., air, precipitation, water, soil, sediments) and various biotic tissue matrices (e.g., muscle, keratin materials such as feather or fur, blood, and eggs) from relevant bioindicators (e.g., fish, sea turtles, birds and marine mammals) that can provide reliable and repeatable measures both temporally and spatially. Ideally the tissue concentrations should also provide information on whether the indicator species are negatively affected by their Hg exposure by linking their Hg body burdens to effect levels determined for related species.

To date, there have been many spatial studies of Hg at the regional scale and temporal studies over timescales of decades in some locations; often these studies focus on locations or areas with known Hg contamination. However, to evaluate the effectiveness of the Convention, a global monitoring system that promotes standardized spatial and temporal coverage is needed. Given that establishing appropriate and effective monitoring frameworks has been a persistent challenge for wildlife conservation (e.g., Tear et al. 2005), increased efforts are needed to embrace more evidence-based approaches for assessing the effectiveness of actions (e.g., Salafsky et al. 2019). The overall goal of this paper is to describe the currently available repositories of peer-reviewed published biotic Hg data and existing monitoring programs to set the stage for developing a global Hg biomonitoring network that can provide accurate information for the assessment of the overall effectiveness of global efforts to reduce the adverse impacts of Hg pollution on people and the environment.

Globally, Hg enters ecosystems through the air (e.g., emissions from coal-fired power plants, incinerators, and volcanic activity), water (e.g., both inactive and active chlor-alkali facilities and artisanal small-scale gold mining), and land (e.g., natural geological formations, mine tailings, landfills, and other contaminated sites) (UNEP 2013; Pacyna et al. 2016; Kocman et al. 2017; Streets et al. 2017, Hsu-Kim et al. 2018; Obrist et al. 2018; Keane et al. 2023). Mercury emitted to the air and released into landscapes, where it can be transported across great distances, remains available for days to years, where its fate is complex as it moves through both terrestrial and aquatic ecosystems into biota (Driscoll et al. 2013; Gustin et al. 2016; Eagles-Smith et al. 2018).

Inorganic Hg emitted from natural or anthropogenic sources becomes more toxic and bioavailable in the environment when it is converted to methylmercury (MeHg), by a wide range of microbial communities (Fleming et al. 2006; Gilmour et al. 2013; Hsu-Kim et al. 2013; Yu et al. 2013). Certain ecosystem conditions (primarily those with an aquatic component, especially wetlands) can encourage the production and bioavailability of MeHg in the environment. Bacteria often produce more MeHg when moderate amounts of sulfate and low oxygen (hypoxic or anoxic) conditions are present to provide optimal conditions for the metabolic processes of the microorganisms (Hsu-Kim et al. 2013, 2018; Hu et al. 2020).

Environmental factors such as water pH, dissolved organic carbon, sulfur concentrations, and land use are important in influencing both inorganic Hg input and methylation potential (Gorski et al. 2008; Wyn et al. 2009; Gabriel et al. 2014; Schartup et al. 2015b; Chaves-Ulloa et al. 2016; Chételat et al. 2018; Rudd et al. 2018; Braaten et al. 2018, 2020). Ecological processes at the base of the food webs such as primary productivity and biomass dilution are also important in the trophic transfer of MeHg from algae to primary and secondary consumers (Pickhardt et al. 2002; Chen et al. 2005; Wu et al. 2019). The complex of redox and biological processes involved in Hg cycling make it particularly challenging to predict levels of potential concern in upper tropic level fish and wildlife from concentrations in air, water, and sediment (Gustin et al. 2016; Sunderland et al. 2016; Eagles-Smith et al. 2018). Ecological and biogeochemical factors play a large role in altering MeHg bioavailability to biota, although Hg concentrations in water and sediment are often not generally correlated with MeHg concentrations in biota (Tsui et al. 2023). Therefore, in sites where Hg deposition or Hg sources are low, levels and effects on biota may still be disproportionately high if the ecological conditions are conducive to MeHg production, bioaccumulation, and biomagnification. For example, MeHg concentrations in fish across freshwater ecosystems in western North America and in estuaries are poorly correlated with either total Hg or MeHg concentrations in aquatic sediments (Eagles-Smith et al. 2016a; Chen et al. 2014; Buckman et al. 2019). The difference between relatively high fish Hg levels in Sweden with low environmental levels of Hg (Braaten et al. 2020) and the low levels of Hg in many fish from waterbodies in China where levels of Hg in sediment, water and even the base of the food web are relatively high, is another example of the importance of understanding relationships between ecological conditions and the levels of biotic Hg. (Wu et al. 2023). The decoupling of inorganic Hg sources from MeHg production and bioavailability is evident at local (Evers et al. 2007) and landscape levels (Eagles-Smith et al. 2016b, 2018; Wang et al. 2023a, 2023b).

Mercury, in its methyl form, is a neurotoxicant and can impair physiological and neurological functions, behavior, reproduction, and survival in fish and wildlife (Scheuhammer et al. 2011; Ackerman et al. 2016; Evers 2018; Whitney and Cristol 2017), as well as humans (Tan et al. 2009; Karagas et al. 2012; Ha et al. 2017; Eagles-Smith et al. 2018; Basu et al. 2023). It readily biomagnifies through foodwebs, resulting in increasing MeHg concentrations as it moves from water and sediment to phytoplankton and plants, zooplankton, aquatic and terrestrial invertebrates, fish, wildlife, and humans. As MeHg moves through the base of the foodwebs, it can efficiently biomagnify in both aquatic and terrestrial organisms. As a result, top predators in foodwebs, including specific species of fish, amphibians, reptiles, birds, and mammals, may have MeHg concentrations in their tissues that are orders of magnitude higher than the concentrations found in water (often >10^6^ to 10^7^ higher). Generally, each trophic increase in the food web accounts for roughly an order of magnitude increase in MeHg concentrations, with the largest enrichment step occurring between water and phyto and zooplankton in aquatic systems (Lee and Fisher 2016; Wu et al. 2019).

Exposure to MeHg has been well documented in fish and wildlife around the world. Contamination can arise directly from inorganic Hg point sources, such as those along rivers (Jackson et al. 2011a; Kinghorn et al. 2007; Nguetseng et al. 2015; Santschi et al. 2017; Geyer and Ralston 2018), around lakes (Anderson et al. 2008; Suchanek et al. 2008; Kumari and Maiti 2019; Chen et al. 2021), and in estuaries (Eagles-Smith and Ackerman 2009; Chen et al. 2014; Buckman et al. 2015; Sullivan and Kopec 2018). Owing to atmospheric transport, inorganic Hg sources may not be local (i.e., <100 km) and subsequent impacts to biota are well described in most continents, including North America (Evers and Clair 2005; Evers et al. 2011a; Ackerman et al. 2016; Eagles-Smith et al. 2016a, b; Evers et al. 2020; AMAP 2021), South America (Sebastiano et al. 2016; May Junior et al. 2017; Manhães et al. 2022), Europe (Åkerblom et al. 2014; Nguetseng et al. 2015; Pacyna et al. 2017), Asia (Kim et al. 2012; Watanuki et al. 2016; Abeysinghe et al. 2017; Noh et al. 2017), Africa (Hanna et al. 2015; van Rooyen et al. 2023), and multiple ocean basins (Carravieri et al. 2014, 2016; Peterson et al. 2015; Drevnick et al. 2015; Lee et al. 2016; Bodin et al. 2017; Drevnick and Brooks 2017; Chastel et al. 2022).

Numerous studies document adverse impacts across many fish and wildlife species. In fish, adverse impacts of MeHg exposure include immunological, reproductive, and behavioral impairment (Hammerschmidt et al. 2002; Depew et al. 2012a; Carvan et al. 2017) as well as reduced capacity for predator avoidance (Webber and Haines 2003). In birds, numerous studies have documented reduced reproductive success, behavioral change (e.g., reduced time incubating), and neurological problems (e.g., ataxia) (Depew et al. 2012a, b; Basu 2015; Ackerman et al. 2016; Evers 2018; Whitney and Cristol 2017; Cristol and Evers 2020). However, many species vary in their sensitivity to MeHg toxicity - potentially based on foraging guilds and phylogeny as identified and discussed by Heinz et al. 2009). For example, embryo survival and hatching success in Passeriforms (i.e., songbirds), appears to be more sensitive to MeHg toxicity than in other orders of birds that have been more extensively studied, such as Anseriformes ducks. In mammals, elevated MeHg concentrations can result in biochemical changes in the brain, ataxia, and reduced reproductive output (Basu et al. 2007; Dietz et al. 2013, 2019, 2021, 2022; Evers 2018; Manhães et al. 2021). Based on these and other in situ studies collectively, the evidence is clear that biomagnification and bioaccumulation of MeHg is shown to adversely affect the reproductive success of many fish and wildlife populations. These biota represent multiple foraging guilds across many habitats and geographic areas of the world.

Understanding exposure pathways of MeHg in terrestrial and aquatic foodwebs and how MeHg adversely affects upper trophic level wildlife is important for developing meaningful assessments and monitoring efforts. Ultimately, identifying the proper fish and wildlife bioindicators for MeHg biomonitoring is complex, because their suitability differs according to geographic area, timescale of interest, conservation concern, and whether the overall goal is for ecological or human health or simply to track changes over time in a consistent and representative species. Herein, we describe some of the regional and global spatial and temporal patterns of MeHg exposure in fish and wildlife based on peer-reviewed literature with an emphasis on relevant bioindicators.

The objectives of this paper are to provide an overview of global, peer-reviewed biotic Hg data to: 1) spatially describe selected global human exposure and ecological bioindicators 2) assess existing biomonitoring data and programs in select regions around the world, and 3) develop strategies for establishing a global Hg biomonitoring framework that can respond to the global strategy defined by the Minamata Convention that calls for establishing an evidence-based monitoring approach to improve our understanding of linkages with Hg sources, spatial gradients and temporal trends.

## Methods

Data within the primary or peer-reviewed literature represent numerous studies that include Hg concentrations in taxa identified in Article 19 of the Minamata Convention on Mercury. Between 2013 and 2023, the Center for Mercury Studies of Biodiversity Research Institute created and maintained a database of Hg concentrations in biotic tissue called the Global Biotic Mercury Synthesis (GBMS). The published data compiled in the GBMS database are summarized here with an emphasis on organisms identified to be of interest for monitoring within the Minamata Convention, which include fish (both teleosts and elasmobranchs), sea turtles, birds, and marine mammals (see Supplementary Information for a listing of the 1701 references used). In some cases, these peer-reviewed sources incorporate data from national monitoring studies, and in other cases are individual scientific studies conducted by governmental agencies, academic researchers, and others. Many Hg concentration measurements in biota, especially fish, have been generated by government agencies around the world. Many of these associated data are not represented here because they are not published in the peer-reviewed literature. However, these unpublished data are significant sources of information that should be collected in a standardized way for evaluating the effectiveness of the Minamata Convention.

Lastly, data for Hg concentrations in tissues that were collected from the published literature include individual (30.4%), composited (4.3%), and averaged (65.2%) data. Mercury concentrations in fish and wildlife are typically log-normally distributed, including the pooled GBMS dataset, and therefore using geometric means are preferable to using arithmetic means. However, the published data were often presented as arithmetic means. Therefore, the composited and averaged data were typically extracted as arithmetic means from the published literature. For consistency, all Hg concentration data presented here are arthimetic means with assoicated standard deviations unless otherwise noted.

For this initial assessment, we did not standardize Hg concentrations. For evaluating the effectiveness of the Minamata Convention, the primary goal will be to evaluate long-term trends in Hg concentrations in fish and wildlife. Because sampling effort and specifics vary among sites and years, it will be important in future trend analyses to account for ecological covariates that are known to influence Hg concentrations in animals. Ecological covariates can be accounted for either in the experimental design (by using a specified subset of the data and excluding any samples that do not meet strict criteria) or during statistical analysis (if sample sizes are sufficient, by including these covariates in the statistical model). For example, in fish, Hg concentrations generally increase substantially with length (Eikenberry et al. 2015), and therefore fish Hg concentrations are generally size-standardized for statistical comparisons (Eagles-Smith et al. 2016a; Drevnick and Brooks 2017). In birds, Hg concentrations generally are higher in males than females (Evers et al. 1998, 2005; Ackerman et al. 2008) and can change with age (Evers et al. 2005; Ackerman et al. 2011). In most wildlife, Hg concentrations vary substantially among sites and day of the year (Ackerman et al. 2019; Chételat et al. 2020). Thus, for effectiveness evaluation of the Minamata Convention, these additional metadata will be important for standardizing Hg concentrations. Several examples of large-scale statistical evaluations of wildlife Hg concentrations are available as a guide (Ackerman et al. 2016; Eagles-Smith et al. 2016a; Drevnick and Brooks 2017; Schoch et al. 2020).

All biotic samples were assigned a Taxonomic Serial Number (TSN) based on the identification provided in the published literature using the Integrated Taxonomic Information System (ITIS) to allow for standardization (ITIS 2023). Species level assignments were made where possible and the lowest taxonomic level that could be reliably assigned was used where species level data was not available.

Fish trophic levels were assigned by species using ‘Fishbase’ and the mean trophic level for the genus or family used, respectively, when presented (Boettiger et al. 2012). The widespread occurrence of ‘fishing down’ is the reason why, in 2004, the Convention on Biological Diversity (CBD) chose the mean trophic level of fisheries catches as an index of the biodiversity of large fishes (defined as fish with trophic levels >3.5), called the *Marine Trophic Index* or MTI (Pauly and Watson 2005). As a result, a threshold of trophic level 3.5 was used for visualization for some graphs.

### Selection of bioindicators

A key initial step in bioindicator selection is to decide whether an organism is linked to a human exposure or ecological health endpoint – which can often be combined for both purposes if carefully considered. Biota that have been identified to best fit these two categories are well described and are categorized within their respective biomes and associated aquatic ecosystems (Table 1). Where applicable, utilization of Indigenous Knowledge is important to incorporate (Houde et al. 2022). Additionally, bioindicators should be reflective of changes in the availability of MeHg in the environment. One of the challenges of using multiple bioindicators is that their Hg concentrations are also affected by food web processes, physical movement, and physiology in addition to changes in the availability of MeHg in the environment (Chételat et al. 2020). Therefore, a key aspect of bioindicator selection for evaluating the effectiveness of reductions in environmental loads of Hg driven by the Minamata Convention is that their concentrations are less sensitive to variability caused by these other factors.

**Table 1:** Potential choices of bioindicator species for ecological and human health as grouped by major terrestrial biomes and their associated aquatic ecosystems (as described and adapted from Evers et al. 2016)

Target terrestrial biomes	Associated aquatic ecosystems		Bioindicators for assessment of potential environmental impact (“ecological health“)				Bioindicators for assessment of potential human exposure (also used for assessing environmental impacts)		
		*Freshwater and marine fish*	*Sea turtles*	*Freshwater/ terrestrial birds*	*Marine birds*	*Mammals*	*Freshwater fish*	*Marine fish*	*Marine mammals*
Arctic tundra	*Arctic Ocean and associated estuaries, lakes, rivers*	Sticklebacks^1^ (fresh/estuary) Arctic Cod^2,169^ Sculpin^3^ Sticklebacks^1^ (marine)		Loons^4,5,141,176^ Shorebirds^100,142,158^	Alcids^112,146^ Fulmars^6,147^ Gulls/Kittiwakes ^61,62, 63, 64, 65,66, 130^ Murres^6,127^ Petrels^123^ Sea Ducks^127^	Polar Bears ^7,99,113,132^ Seals^8, 113^	Arctic Char^9,60^ Arctic Burbot^67^ Grayling^10^	Halibut^202,203^ Cod^204,205,206,207^	Beluga^2, 12,99,222^ Narwhal^2,12^ Seals ^8,57,99,^ ^120,126,174^
Boreal forest and taiga	*North Pacific and Atlantic Oceans and associated estuaries, lakes, rivers*	Perch^13,170,171^ (fresh/estuary) Mummichogs & Silversides^14, 82^ Gobies/ Sticklebacks^101,102^ (marine)		Eagles^184^ Falcons^71,180^ Loons^5,15,139,141,144,177^ Osprey^17,96,98^ Songbirds^18^ (blackbirds, flycatchers, warblers)	Eagles^105,178,185^ Gulls^58,68^ Osprey^19^ Storm-Petrels/ Petrels^20,123,150^ Sea Ducks^107^ Skuas^157^	Mink^21,22^ Otter^21,22^ Sea Lions^124^ Seals^23,135^	Lake Trout^69,70^ Pike^10,70,183^ Sauger^10^ Walleye^10,70,183^	Flounder^201^ Tuna^197^	Pilot Whale ^24,173^
Temperate broadleaf and mixed forest	*Mid Pacific and Atlantic Oceans, Mediterranean Sea, associated estuaries, lakes rivers* *(Note: South Pacific and Atlantic Oceans in temperate zone and Southern Ocean placed here)*	Perch^13,172^ Catfish^152,167^ Cyprinnids^78,83^ (fresh/estuary) Mummichogs/ Silversides^14, 82,182^ Sticklebacks^25,101,^ ^102^ (marine)	Sea turtles ^29, 52,181^	Eagles^16,72,92,175,177^ Egrets/Herons^27,208,217^ Grebes^5,26,71,95^ Gulls^59,138^ Kingfishers^211,214,215^ Loons^4,139,140^ Osprey^17,19,91,92,93,94,95,171^ Shorebirds^104^ Terns^26,101,103^ Songbirds^18,35,81,210,211,221^ (blackbirds, flycatchers, sparrows, swallows, warblers, wrens)	Albatrosses^128,137^ Boobies^116,117,118^ Cormorants^28,118^ Eagles^72,179^ Frigatebird^116^ Gulls^68,138^ Osprey^5,19,161^ Pelicans^73,137, 159^ Penguins^48,137,149,159, 163,228, 229^ Shearwaters^76^ Skuas^49,155^ Terns^26,28^	Bats^85,86,87,88^ Dolphins & Porpoises^53,54,90,98,131,223^ Otter^21,22,119,224^ Sea Lions^125,225^ Seals^23,97,115,121,^	Bass^10,30,31^ Catfish^167^ Lake Trout^70^ Pike^70,164^ Walleye^31,70^	Mackerel^199,200^ Sharks^11,32, 129,148,218,227^ Billfish^11,45,134,136,192^ Tuna^32,45,195,196,197^	
Tropical rainforest	*South Pacific and South Atlantic and Indian Oceans, Caribbean Sea, and associated estuaries, lakes, rivers*	Catfish^23, 145,165,166^ Piranha^34,109,160^ Snook^208^ (fresh/estuary) Lionfish^188^ Bay Snook^34^ (marine)	Sea turtles ^29,143,181^	Egrets/Herons^27^ Kingfishers^212,213^ Shorebirds^153,162^ Songbirds^11,36^ (antbirds, warblers, woodcreepers, wrens)	Albatrosses^37,38,50,51^ Boobies^131,219^ Frigatebirds^4,74,75,116,131^ Noddy^39, 47,219^ Storm-Petrels/ Petrels^51, 220^ Shearwaters^39,76,111^ Terns^39,47,156,162,219^ Tropicbirds^39,133^	Beaked Whale^56^ Dolphins^111,114^ Jaguar^89^ Otter^40,80^ Pilot Whale^55^ Seals^41,226^	Catfish^166,168^ Nile perch^79^ Peacock bass^33^ Snakehead^209^ Tigerfish^84^ Wolffish^110^	Barracuda^186,187,188^ Grouper^42,189,190^ Mahi-mahi^108,151,188^ Sharks^43,44,46,122^ Snapper^188,191^ Swordfish^46,192,193^ Tuna^46,77.106,194,198^	

Note that bioindicators for human exposure are also useful for environmental assessments

^1^Kenney et al. (2014), ^2^AMAP 2011), ^3^Rigét et al. (2007), ^4^Evers et al. (2014), ^5^Jackson et al. (2016), ^6^Braune 2007), ^7^Routti et al. (2011), ^8^Dietz et al. (2013), ^9^Gantner et al. (2010), ^10^Eagles-Smith et al. (2016a), ^11^Sayers et al. (2023), ^12^Wagemann and Kozlowska (2005), ^13^Wiener et al. (2012a), ^14^Weis and Khan (1990), ^15^Evers et al. (2011b), ^16^Bowerman et al. (1994), ^17^Odsjö et al. (2004), ^18^Jackson et al. (2015), ^19^Wiemeyer et al. (1988), ^20^Goodale et al. (2008), ^21^Yates et al. (2005), ^22^Klenavic et al. (2008), ^23^Brookens et al. (2008), ^24^Dam and Bloch (2000), ^25^Eagles-Smith and Ackerman (2009), ^26^Ackerman et al. (2016), ^27^Frederick et al. (2002), ^28^Braune (1987), ^29^Day et al. (2005), ^30^Kamman et al. (2005), ^31^Monson et al. (2011), ^32^Cai et al. (2007), ^33^Bastos et al. (2015), ^34^Mol et al. (2001), ^35^Lane et al. (2011), ^36^Townsend et al. (2013), ^37^Finkelstein et al. (2006), ^38^Burger and Gochfeld (2000), ^39^Kojadinovic et al. (2007), ^40^Fonseca et al. (2005), ^41^Marcovecchio et al. (1994), ^42^Evers et al. (2009), ^43^Kiszka et al. (2015), ^44^Maz-Courrau et al. (2012), ^45^Storelli and Marcotrigiano (2001), ^46^Bodin et al. (2017), ^47^Sebastiano et al. (2017), ^48^Carravieri et al. (2016), ^49^Carravieri et al. (2017), ^50^Bustamante et al. (2016), ^51^Anderson et al. (2010), ^52^Maffucci et al. (2005), ^53^Correa et al. (2013), ^54^Aubail et al. (2013), ^55^Bustamante et al. (2003), ^56^Garrigue et al. (2016), ^57^Brown et al. (2016), ^58^Burgess et al. (2013), ^59^Weseloh et al. (2011), ^60^Evans et al. (2015), ^61^Braune et al. (2006), ^62^Lucia et al. (2015), ^63^Lucia et al. (2016), ^64^Braune et al. (2016), ^65^Miljeteig et al. (2009), ^66^Bond et al. (2015), ^67^Pelletier et al. (2017), ^68^Blukacz-Richards et al. (2017), ^69^Abma et al. (2015), ^70^Gandhi et al. (2014), ^71^Hartman et al. (2017), ^72^DeSorbo et al. (2018), ^73^Newtoff and Emslie 2017), ^74^Sebastiano et al. (2016), ^75^Mott et al. (2017), ^76^Watanuki et al. (2016), ^77^Bosch et al. (2016b), ^78^Cerveny et al. (2016), ^79^Hanna et al. (2016), ^80^Soresini et al. (2021), ^81^Pacyna et al. (2017), ^82^Chen et al. (2014), ^83^Buckman et al. (2015), ^84^Webb et al. (2015), ^85^Yates et al. (2014), ^86^Little et al. (2015),^87^Åkerblom and de Jong 2017), ^88^Korstian et al. (2018), ^89^May Junior et al. (2017), ^90^Titcomb et al. (2017), ^91^Rumbold et al. (2017), ^92^Kalisinska et al. (2014), ^93^Hughes et al. (1997), ^94^Henny et al. (2009), ^95^Anderson et al. (2008), ^96^DesGranges et al. (1998), ^97^Peterson et al. (2016b), ^98^Reif et al. (2015), ^99^Krey et al. (2015), ^100^Perkins et al. (2016), ^101^Eagles-Smith and Ackerman (2009), ^102^Eagles-Smith and Ackerman (2014), ^103^Ackerman et al. (2008), ^104^Ackerman et al. (2007), ^105^Burger and Gochfeld (2009), ^106^Chouvelon et al. (2017), ^107^Savoy et al. (2017), ^108^Adams (2009), ^109^Ouboter et al. (2012), ^110^da Silva et al. (2019), ^111^Durante et al. (2020), ^112^Albert et al. (2021), ^113^Lippold et al. (2021), ^114^Manhães et al. (2021), ^115^Lian et al. (2021), ^116^Gilmour et al. (2019a), ^117^Bighetti et al. (2021), ^118^Le Croizier et al. (2022a), ^119^Dibbern et al. (2021), ^120^Houde et al. (2020), ^121^Damseaux et al. (2021), ^122^Amorim-Lopes et al. (2020), ^123^Pollet et al. (2023), ^124^Rea et al. (2020), ^125^Castellini et al. (2022), ^126^Ewald et al. (2019), ^127^Burnham et al. (2021), ^128^Mills et al. (2020), ^129^Kim et al. (2016), ^130^Fleishman et al. (2019), ^131^Machovsky-Capuska et al. (2020), ^132^Lippold et al. (2022), ^133^Zarn et al. (2020), ^134^Rudershausen et al. (2023), ^135^MacMillan et al. (2022), ^136^Biton-Porsmoguer et al. (2022), ^137^Furtado et al. (2019), ^138^Sánchez-Fortún et al. (2020), ^139^Evers et al. (1998), ^140^Evers et al. (2003), ^141^Eriksson et al. (1992), ^142^Hargreaves et al. (2010), ^143^Mondragón et al. (2023), ^144^Burgess et al. (2005), ^145^Makaure et al. (2023), ^146^Pacyna-Kuchta et al. (2020), ^147^Braune et al. (2010), ^148^Erasmus et al. (2022a), 2022b), ^149^Dodino et al. (2022), ^150^Furtado et al. (2021), ^151^Ahmed et al. (2020), ^152^Zhang et al. (2014), ^153^Pandiyan et al. (2020), ^154^Motas et al. (2021), ^155^Mills et al. (2022), ^156^Cusset et al. (2023), ^157^Albert et al. (2022), ^158^Pratte et al. (2020), ^159^Quadri-Adrogué et al. (2022), ^160^de Matos et al. (2021), ^161^Hopkins et al. (2007), ^162^Correia et al. (2023), ^163^Pilcher et al. (2020), ^164^Chalabis-Mazurek et al. (2021), ^165^Utete and Fregene (2020), ^166^Elawady et al. (2019), ^167^Squadrone et al. (2015), ^168^Azevedo et al. (2012), ^169^Campbell et al. (2005), ^170^Wu et al. (2018), ^171^Hughes et al. (1997), ^172^Strandberg et al. (2016), ^173^Gajdosechova et al. (2016), ^174^Brunborg et al. (2006), ^175^Bechard et al. (2009), ^176^Schmutz et al. (2009), ^177^Bjedov et al. (2023), ^178^Dominguez et al. (2003), ^179^Jagoe et al. (2002), ^180^Keyel et al. (2020), ^181^Rodriguez et al. (2022), ^182^Baumann et al. (2017), ^183^Packull-McCormick et al. (2023), ^184^Sun et al. (2019), ^185^Anthony et al. (2007), ^186^Ritonga et al. (2023), ^187^Rumbold et al. (2018), ^188^Christian et al. (2024), ^189^Condini et al. (2023), ^190^Sinkus et al. (2021), ^191^Bank et al. (2007), ^192^Esposito et al. (2018), ^193^Jinadasa et al. (2013), ^194^Jinadasa et al. (2018), ^195^Drevnick et al. (2015), ^196^Médieu et al. (2022), ^197^Tseng et al. (2021), ^198^Houssard et al. (2019), ^199^Adams and McMichael (2007), ^200^Costa et al. (2021), ^201^Leah et al. (1992), ^202^Jewett and Duffy (2007), ^203^Bentzen et al. (2016), ^204^Gopakumar et al. (2021), ^205^Ruus et al. (2015), ^206^Ono et al. (2019), ^207^Cladis et al. (2014), ^208^Mol et al. (2001), ^209^Lobus et al. (2011), ^210^Edmonds et al. (2010), ^211^Evers et al. (2005), ^212^Oliveira et al. (2023), ^213^Hurtado et al. (2023), ^214^Zamani-Ahmadmahmoodi et al. (2009), ^215^White and Cristol (2014), ^216^Hill et al. (2008), ^217^Goutner and Furness (1997), ^218^Alves et al. (2023), ^219^Bustamante et al. (2023), ^220^Quillfeldt et al. (2023), ^221^Kopec et al. (2018), ^222^Yurkowski et al. (2023), ^223^Sedak et al. (2022), ^224^Ryazanov et al. (2023), ^225^Symon et al. (2023), ^226^Charapata et al. (2023), ^227^Riesgo et al. (2023), ^228^Gimeno et al. (2024), ^229^Gilmour et al. 2019c

**Fig. 1:**
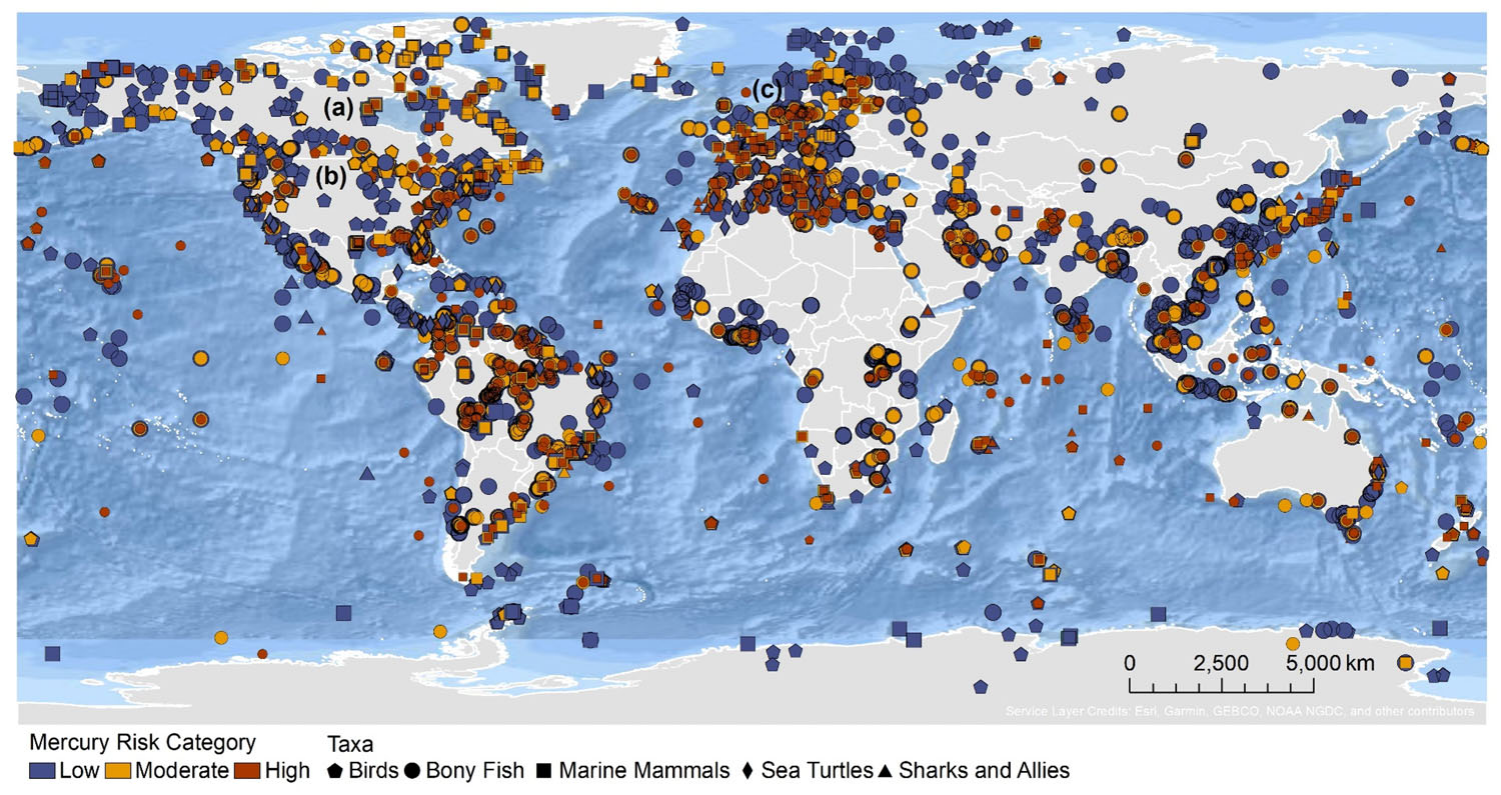
Distribution of five major taxa and their total Hg concentrations in three risk categories based on mean data derived from a survey of the available peer-reviewed English literature. Risk categories by major taxa and tissue type are: (1) cartilaginous fish (sharks and allies) and (2) bony fish muscle (µg/g, ww): <0.23 = low, 0.23–0.46 = moderate, >0.46 = high; (3) sea turtle muscle and egg (µg/g, ww): <0.22 = low, 0.22–0.46 = moderate, >0.46 = high; (4) bird body feathers (adult; µg/g, fw): <10.0 = low, 10.0–20.0 = moderate, >20.0 = high; bird blood (adult; µg/g, ww): <1.0 = low, 1.0–3.0 = moderate, >3.0 = high; eggs (µg/g, ww): <0.5 = low, 0.5–1.0 = moderate, >1.0 = high; (5) marine mammal muscle (µg/g, ww): <0.22 = low, 0.22–0.46 = moderate, >0.46 = high. Letters indicate additional available fish Hg samples that were not mapped: **a** >330,000 additional fish Hg concentrations within the Canadian Fish Mercury Database (Depew et al. 2013); **b** an estimated >500,000 additional fish Hg concentrations available within state databases in the United States, and; **c** >54,000 additional fish Hg concentrations within Fennoscandia (Braaten et al. 2019). Data for migratory species are plotted according to the reported location of sampling, which in some cases may not reflect the region where Hg exposure occurs

The extensive datasets of Hg concentrations in biota found in the published literature provide a basis for choices of species for potential monitoring (Fig. 1; Evers et al. 2016). Careful selection can ensure comparability at regional and global scales. A systematic literature search (range of years covered was 1972 to 2023) emphasized Hg data from: (1) biota identified in Article 19 of the Minamata Convention (fish, sea turtles, birds, and marine mammals), (2) species for human consumption, (3) taxonomic groups at high risk of MeHg exposure, (4) potential bioindicators for MeHg monitoring purposes, and (5) species from areas of concern due to current significant Hg sources (e.g., coal-fired power plants and artisanal small-scale gold mining [ASGM]). The taxonomic presentation structure follows phylogenetic order.

Because the selection of the taxa recommended for bioindicator species emphasizes the animal groups identified in the Minamata Convention’s Article 19, and their ability to represent MeHg exposure in a particular system, invertebrates are not considered. Therefore, organisms such as phyto and zooplankton are not included for biomonitoring given their high temporal and spatial variability within waterbodies and that they require maximizing within-date replication and higher frequency sampling during a season (Ward et al. 2012; Chen et al. 2012). In addition, the percent MeHg concentrations (generally less than 75%) are not as high as those in higher trophic level fish (>95%; Driscoll et al. 2007).

Lastly, published studies included here are those for which there is reasonable confidence about their validity, including those with: (1) sufficient description of the characteristics of the organism sampled (i.e., species, date, location, size/age, and tissue analyzed); (2) an appropriate method of sample collection; and (3) detailed information on sampling location (i.e., market-based fish Hg concentrations are excluded). For North America, extensive biotic Hg datasets published in response to three regional, one state, and one National Park synthesis efforts are included for the: (1) northeastern United States and eastern Canada (Evers and Clair 2005), (2) Great Lakes Region of the United States and Canada (Evers et al. 2011a), (3) western United States and Canada (Eagles-Smith et al. 2016b), (4) New York State (Evers et al. 2020), and (5) Acadia National Park, Maine, United States (Burton et al. 2024); although, all of the data are not mapped (Fig. 1 and see qualifiers “a” and “b”).

The data collected and incorporated into GBMS represent the arithmetic mean, or individual sample concentrations (when available), standard deviation (SD), minimum and maximum values, total number of individuals for each species, and tissue type that could be georeferenced within a peer-reviewed publication. These data were then joined by taxa and tissue type to generate a global average and variation. The raw data underlying the averaged statistics used herein were not always available and therefore, individual metadata of biota were not included (e.g., no adjustments or normalization for age, sex and size were conducted). Each of the published studies’ Hg concentrations was mapped by major taxonomic group (i.e., cartilaginous and bony fish, sea turtles, birds and marine mammals) and tissue type and were placed in three risk categories based on human exposure or ecological health thresholds (i.e., low, medium and high).

### Risk categories

The health-related risk categories based on human exposure are developed from a combination of benchmarks pertaining to animal tissues consumed by humans and generated from standards used in the United States (USFDA 2022), for Arctic communities (AMAP 2015), and by the World Health Organization that generally relate to MeHg exposure levels of concern recognized for humans (Višnjevec et al. 2014; Basu et al. 2018). For human populations, those most at risk of MeHg exposure include: (1) sensitive individuals (e.g., women of childbearing age, pregnant women, and children); (2) communities dependent on a diet of aquatic organisms (e.g., Indigenous and subsistence fish consumers); and (3) diets regularly including high trophic-level fish (e.g., recreational anglers). The greatest risks to humans from dietary uptake of MeHg are observed with high consumption of upper trophic level species. For example, primary consumers (e.g., shellfish such as mussels) at trophic level 2 generally have relatively low MeHg concentrations and are usually considered safe for consumption (Chase et al. 2001). Secondary consumers (e.g., salmon, herring) are at trophic level 3, but are usually considered to be healthy choices.

For tertiary or higher consumers, which are carnivorous fish that generally consume vertebrate prey, MeHg concentrations can be elevated to levels that trigger human health concerns. The variability of concentrations in fish with a trophic level of 3.5 or more can be related to size, species, and location (Keppeler et al. 2020). Therefore, fish exceeding a trophic level of 3.5 that are commonly harvested are important bioindicators to assess potential exposure risk of Hg to humans. Large marine predatory fish such as tuna, swordfish and shark can have elevated MeHg concentrations, frequently exceeding the no consumption limits (or choices to avoid) identified by the USEPA and U.S. Food and Drug Administrations (USFDA [i.e., 0.46 µg/g, ww; Table 2]). While the European Union (EU) identifies the maximum level for total Hg in fish muscle for human consumption is 0.5 μg/g ww, they also include an exception for ‘large predators’ for which the maximum level is 1.0 μg/g ww (EU 2023). The USEPA - USFDA consumption limits are used herein.

**Table 2:** Fish Hg concentrations and meal frequency ingestion for people, based on U.S. guidelines^a^

Guideline or criterion by agency	Mercury in fish (µg/g, ww)^c^	Fish consumption guideline
U.S. Environmental Protection Agency (USEPA) – U.S. Food and Drug Administration (USFDA) fish advice^b^	<0.15	Best choices; 2–3 meals per week
	<0.23	Good choices; 2 meals per week
	<0.46	Good choices; 1 meal per week
	>0.46	Choices to avoid; 0 meals per week

^a^Mercury concentrations are interpreted in the context of the number of fish meals that could be consumed to stay within the USEPA health-based reference dose for MeHg adopted in 2001 (Stern 2005)

^b^USFDA (2022); https://fda.gov/food/environmental-contaminants-food/technical-information-development-fdaepa-advice-about-eating-fish-those-who-might-become-or-are

^c^Note that only U.S. guidelines are used as thresholds for this paper; other guidelines are available through other entities, including the European Union (EU), which has guidelines based on Hg concentrations in the water (EEB 2021) for ecological health and there are EU and World Health Organization human health guidelines, which are higher than those identified by the USEPA and USFDA

The impacts of MeHg on fish health and reproductive welfare are not well established, but have been summarized previously (Depew et al. 2012a, b; Table 3a) and include threshold limits identified by Sandheinrich et al. (2011). While fish Hg concentrations are commonly examined for their impacts on humans (i.e., muscle tissue) or for wildlife exposure (e.g., whole body), the MeHg concentrations in fish tissues also can be assessed for their impact on behavior, reproductive abilities, and overall health.

**Table 3:** a. Screening benchmarks of MeHg in the diet of piscivorous fish and birds for reproductive endpoints. b. Estimated effect Hg concentrations in two broad feeding groups of birds – fish eaters (piscivores) and invertebrate eaters (invertivores)

a.		
Taxa	Screening benchmark: dietary MeHg (µg/g, ww)^a^	Endpoint of interest
Fish	>0.04	Potential impaired reproductive success (Depew et al. 2012a)
	>0.20	Probable impaired reproductive success (Sandheinrich et al. 2011)
	>0.50	Adverse effects in behavior (Depew et al. 2012a)
Birds	0.10–0.18	Adverse effects on behavior (Depew et al. 2012b)
	0.18–0.40	Impaired reproductive success (Depew et al. 2012b)
	>0.40	Reproductive failure (Depew et al. 2012b)

b.							
Feeding group	Species used for designation	Tissue type/effect endpoint	EC10	EC20	EC30	EC40	Reference
Bird - Piscivores	Common Loon (*Gavia immer*)	Adult blood/fewer fledged young	1.5 µg/g	2.0 µg/g	2.5 µg/g	3.0 µg/g	Burgess and Meyer (2008); Evers et al. (2008)
		Egg/lowered hatching success	0.48 µg/g	0.65 µg/g	0.80 µg/g	0.98 µg/g	Evers et al. (2003, 2008)
		Adult feather/equivalent to fewer fledged young^b^	10 µg/g	20 µg/g	30 µg/g	40 µg/g	Evers et al. (2008),
Bird – Invertivores	Carolina Wren (*Thryothorus ludovicianus*)	Adult blood/lowered nesting success	0.70 µg/g	1.2 µg/g,	1.7 µg/g	2.2 µg/g	Jackson et al. (2011a)
		Egg/equivalent to lowered nesting success	0.11 µg/g	0.20 µg/g	0.29 µg/g	0.36 µg/g	Jackson et al. (2011a)
		Adult body feather/equivalent to lowered nesting success	2.4 µg/g	3.4 µg/g	4.5 µg/g	5.3 µg/g	Jackson et al. (2011a)

Each group is represented by a single species with sufficient data to illustrate using an endpoint of lowered reproductive success for four levels of impact. Tissue type concentrations are shown for blood and eggs (wet weight, ww) and feathers (fresh weight, fw) according to effect concentrations (EC) by percentage (e.g., EC_10_ denotes a 10% effect level)

^a^Assuming dietary Hg is adjusted for >95% MeHg

^b^Adult piscivore feather ECs are estimated and are based on the finding for an EC_40_ that is 40 µg/g (fw) in secondary feathers

Fish may exhibit impaired reproductive success at relatively low dietary MeHg concentrations as low as 0.04 µg/g, ww (Depew et al. 2012a) and may have adverse visible behavioral impacts at dietary MeHg concentrations of 0.50 µg/g, ww or higher (Depew et al. 2012a) (Table 3a). A recent synthesis of the effects of Hg on freshwater fish further summarizes adverse effects at physiologic, histologic, biochemical, enzymatic, and genetic levels; and that some fish species demonstrate greater sensitivity to MeHg than others (Morcillo et al. 2017). Ultimately, lower reproductive success reduces the size and sustainability of healthy fish populations, which could have adverse impacts on associated populations of piscivores and human recreational and commercial interests. Unlike freshwater fish, there have been few rigorous published studies evaluating toxicity of MeHg to marine fish (Scheuhammer et al. 2015; Morcillo et al. 2017).

For understanding risk to the health of birds, known risk categories for diet (Table 3a) and various tissue types (e.g., eggs, blood, and feathers) are well-established for some piscivores and invertivores (Table 3b). The science behind characterizing risk benchmarks that are based on both laboratory and wildlife populations has improved significantly from the initial study of lab-based lowest-observed adverse effect levels on a single species – the mallard (*Anas platyrhynchos*) (Heinz 1979) to more recent efforts. Current research documents effect concentrations related to different levels of breeding success in invertivore songbirds (Carolina wren, *Thryothorus ludovicianus*) and piscivorous waterbirds (common loons, *Gavia immer*) and is well supported by data collected in both laboratory and wild bird populations (Ackerman et al. 2016; Evers 2018; Whitney and Cristol 2017).

Adverse effect thresholds are not as well established for marine mammals, primarily because of field study and ethical challenges (Dietz et al. 2022). Brain Hg concentrations were found to have a significant positive correlation with liver concentrations, and brain Hg concentrations reported in cetaceans were one order of magnitude higher than pinnipeds and generally exceeded neurotoxicity thresholds (López-Berenguer et al. 2020).

### Preferred tissue types and important metadata

Understanding the pharmacodynamics of Hg species concentrations in organisms is important because MeHg biomagnifies through foodwebs in polar (Ruus et al. 2015; Seco et al. 2021; Matias et al. 2022), temperate (Arcagni et al. 2018), and tropical (Bisi et al. 2012; Seixas et al. 2014) ecosystems, and bioaccumulates over time in individual fish (Drevnick and Brooks 2017), birds (Evers et al. 1998), and marine mammals (Lailson-Brito et al. 2002, 2012; Krey et al. 2015). The cycling, speciation, and toxicology of Hg can vary substantially among different tissues, which can have important implications for interpreting Hg concentrations (Manhães et al. 2021). Understanding how the selection of tissue types dictate interpretative power in the bioaccumulation and biomagnification of MeHg and subsequent potential health impacts is a critical aspect for developing monitoring designs (Eagles-Smith et al. 2016b; Chételat et al. 2020). Additionally, to establish relevant species, tissues, and timing of sampling of importance for human Hg exposure in Indigenous Populations, Indigenous Knowledge can provide crucial information (AMAP 2021; Houde et al. 2022).

This review focuses on tissues with well-established methods of measurement and interpretation and for which there is a large body of data and are regularly used for monitoring purposes (Table 4). There are many available matrices and tissue choices dependon monitoring objectives, interests, and outcomes. Often the most useful tissues can be non-lethally collected in the field. Samples that can be analyzed to assess total Hg or MeHg exposure are often from tissue types for targeted biotic groups (Table 4). Composite samples are sometimes used to estimate population Hg concentrations at a decreased cost (Gandhi et al. 2016) and are especially useful for cost-effective long-term trend assessments (Gandhi et al. 2016). Because most of the Hg in tissues that are commonly tested for biomonitoring purposes is in the MeHg form (i.e., generally >95%), analyses of total Hg (which is less expensive to analyze) is also more cost effective. The development of direct analyzers that couple thermal decomposition with Hg amalgamation and atomic absorption detection has simplified Hg determination and made analysis more accessible to those without advanced and costly laboratory facilities (Windmöller et al. 2017).

**Table 4:** Major biota groupings and tissues identified for MeHg monitoring using non-lethal methods

Biota group	Tissue type	% MeHg	Sample preparation type^a^	Analysis type	Source reference for % MeHg	Comments about use of tissue type
Fish	Muscle fillet	75–95% (but varies on average as low as 65%)	ww or dw	THg or MeHg	Bloom (1992); Lescord et al. (2018)	Recent evidence indicates that %MeHg may be lower for some fish species (Manceau et al. 2021a) and for some cooking approaches (Wang et al. 2013) so to confirm the expected amounts. 10% of fish should be analyzed for MeHg content.
	Muscle biopsy	75–95% (but varies)	dw	THg	Peterson et al. (2004)	dw is best because of moisture loss concerns. Muscle biopsy to muscle fillet has a r^2^ = 0.96. Biopsy plug depth may impact Hg measured – 5 mm plugs are best below dorsal fin (Cizdziel et al. 2002) and are without skin and adipose tissue.
	Fin clips, muscle fillet and whole body	varies	dw	THg or MeHg	Cerveny et al. (2016)	There is a significant correlation between fin clips and muscle fillet/whole body (*p* < 0.01).
	Blood	>95%	ww or dw	THg		Assumed to be >95% MeHg based on other vertebrates.
Sea turtles	Scutes, carapace fragments, and nails	~10%	fw (or dw if scutes need washing)	THg	Rodriguez et al. (2019); Benjamin et al. (2018)	While scutes are keratinized material the %MeHg may be relatively low and needs more data.
	Blood	>95%	ww or dw	THg		Assumed to be >95% MeHg based on other vertebrates.
	Muscle	>95%?	ww or dw	THg		Assumed to be >95% MeHg based on other vertebrates.
Birds	Blood	>95%	ww or dw	THg	Rimmer et al. (2005); Edmonds et al. (2010)	Elimination of MeHg in adult blood comprises an initial fast phase, with half-time of one day, and a slow terminal phase with half- time between 44–65 days. Molt is a crucial factor in determining the rate of MeHg elimination (Monteiro and Furness 2001; Fournier et al. 2002a, 2002b). Body condition is important for proper interpretation of blood Hg levels (Mallory et al. 2018). Developing nestling have lower blood Hg concentrations due to depuration into feathers (Ackerman et al. 2011).
	Dried Blood on Whatman cards	>95%	dw	THg	Perkins and Basu (2018)	Sayers et al. (2023) supports this approach with new caveats.
	Feather	~100%	dw or fw whole feathers	THg	Burger (1993), Renedo et al. (2017)	Use feathers with care for interpretation; see Peterson et al. (2019) for a tool and guidelines for feather processing, analysis, and Hg interpretation. Feather tips can correlate with the complete feather (Ma et al. 2021).
	Rhamphotheca	>95%	fw	THg		Outer surface of the beak that consists of a thin sheath of keratin and were 2-fold > than in P1 and S8 feathers (de Medeiros Costa et al. 2021).
	Nails or claws or talons	>95%	fw	THg	Hopkins et al. (2007)	Total Hg gull ‘claws’ better reflect Hg concentrations in internal organs vs. feathers (Grajewska et al. 2019).
	Eggs	>96%	dw or ww	THg	Ackerman et al. (2013) (96% for 22 species)	Wet weight can be problematic if eggs are not collected immediately after laying because of potential moisture loss (Stickel et al. 1973; Dolgova et al. 2018).
	Eggshells and membranes	>95%	dw	THg	Peterson et al. (2017)	Membranes are assumed to be primarily MeHg, but shells are entirely inorganic Hg.
	Muscle	>95%	ww or dw	THg	Renedo et al. (2021)	MeHg comprised over 99% of total Hg in breast muscle of waterfowl (Sullivan and Kopec 2018).
Marine mammals	Skin	>90%	dw	THg	Wagemann et al. (1998)	Muktuk (in marine mammals) includes layers of skin and blubber.
	Fur or hair	>90%	dw (or fw if fur is not washed)	THg	Evans et al. (2000)	Use fur with caution; fur/hair may not relate to blood and muscle depending on growth patterns (Peterson et al. 2016a, b).
	Whiskers (vibrissae)	>90%	dw	THg		Whiskers especially useful for pinnipeds (Rea et al. 2020) and can be aged and provide a time series for Hg (Charapata et al. 2023)
	Muscle	>90%	ww or dw	THg	Wagemann et al. (1998)	

^a^Sample preparation type that is underlined is the preferred type, but the alternative is acceptable if properly performed; Reported as wet weight (ww), dry weight (dw), and/or fresh weight (fw). Fw denotes keratin-based samples that are not cleaned or dried prior to total Hg (THg) analyses

Other metadata that are important to improve interpretive power include physiological, demographic, and ecological factors (Chételat et al. 2020). For example, accounting for the health and fitness of indicator organisms is important for standardized comparisons, as is the identification of species, size, age, and sex. Covariation between Hg concentration and fish size (length and weight) and age requires a standardization to allow for investigation of temporal trends of Hg concentrations. However, for most of this data compilation, biotic Hg concentrations were not indexed or standardized according to size, age, or sex. This is a weakness of the dataset and is an important reason for designing a standardized sampling framework to strengthen the ability to interpret the data.

In general, larger and older individuals have higher MeHg concentrations than smaller and younger individuals, and males that are larger in body size than females tend to have higher concentrations in fish and birds (Evers et al. 2005; Robinson et al. 2012; Ackerman et al. 2008, 2015, 2016; Hartman et al. 2017), with a few exceptions related to foraging segregation between sexes like in albatrosses (Carravieri et al. 2014). An exception from the evaluation of fish Hg concentrations without data on age and/or size are fish Hg databases in Scandinavia (Braaten et al. 2019) and across North America (Kamman et al. 2005; Monson et al. 2011; Eagles-Smith et al. 2016a). Braaten et al. (2019) used the individual fish weight and Hg concentration in combination with fish species information and sampling year to find the modeled (i.e., expected) Hg concentration for fish at a standard weight; there are similar findings in tuna species as well (Médieu et al. 2021, 2022).

Changes in an animal’s physiology, health status, or ecological life history events can also have a substantial effect on MeHg concentrations, regardless of an animal’s actual environmental MeHg exposure. For example, the maternal transfer of MeHg to offspring during reproduction can reduce the female’s tissue concentrations of MeHg but increases risk to offspring, and the amount of MeHg transferred from females to their offspring differs among species (Ackerman et al. 2020). Weight change can also influence the interpretation of MeHg concentrations in animals. For instance, rapid growth of juvenile birds can cause mass dilution of contaminants and substantially reduce MeHg concentrations as juvenile birds age (Ackerman et al. 2011). Rapid growth in fish can also result in “growth dilution” and has been measured in freshwater and marine fish (Ward et al. 2010; Baumann et al. 2017). In contrast, annual life changes in adult body mass, such as fasting- and breeding-associated declines in body mass during periods of haul-out on land for marine mammals, can substantially increase MeHg concentrations (Peterson et al. 2018). In the same way, infections can cause MeHg remobilization and changes its body distribution (Manhães et al. 2021).

Seasonality can have large implications for biotic Hg monitoring programs (Eagles-Smith and Ackerman 2009; Braaten et al. 2014). Seasonal changes in MeHg exposure may be related to changing methylation rates and bioavailability in estuaries (e.g., saltmarsh sparrows, *Ammodramus caudacutus*, increase in blood Hg concentrations from early to late summer; Lane et al. 2011), molt strategies (Condon and Cristol 2009), migratory patterns for birds (Ackerman et al. 2019) and arrival to over-wintering areas (Eagles-Smith et al. 2009a), or lake-specific variation in Hg dynamics (e.g., Clark’s and western grebes, *Aechmophorus clarkia and A. occidentalis*, decrease in blood Hg from spring to autumn; Hartman et al. 2017). Lower food availability in winter can also result in losses in body condition factor and increases in Hg concentrations in fish tissue (Martyniuk et al. 2020; Piro et al. 2023).

Lastly, as outlined in AMAP (2021), and summarized by Houde et al. (2022), Indigenous Knowledge provides invaluable information for the interpretation of tissue Hg concentrations in the Arctic environment and should be appropriately utilized together with scientific evidence for a holistic and comprehensive analysis. Examples include explaining Hg concentrations in whitefish in Nunavut, Canada, that were lower than expected. Indigenous Knowledge explained that whitefish migrated out to sea to feed after the ice went out, which was not known to scientists, and explained the lower Hg levels that were found in these fish. Similarly, research in Nunavik found elevated levels of selenoneine in the blood of women. Selenoneine is a protective compound against negative impacts of Hg. Knowledge holders explained that this could be connected to only women eating the tail of the beluga, and analysis confirmed that selenoneine concentrations in the skin of the beluga tail are nearly twice as high compared to the skin from other areas of the whale (AMAP 2021; Houde et al. 2022). Other research on beluga whales in Nunavik investigated Indigenous Knowledge including on migration, body condition, foraging ecology, predation, breeding, calving and behavior of animals - all of which can help understand beluga exposure to Hg and other contaminants (Breton-Honeyman et al. 2016). Indigenous Peoples have lived on their lands for hundreds or even thousands of years, in an intricate relationship with their environment, with knowledge being passed on through many generations and ensuring their survival. Consequently, they have the most intimate understanding of their ecosystems and their complex connections, as well as any changes that occurred over time – be it in the Arctic, the Amazon, or other biomes.

## Results

Biotic Hg concentrations for targeted taxa (based on Article 19 of the Minamata Convention) were collected from over 1700 peer-reviewed (See Supplementary Materials) scientific publications that represent >588,000 individuals at over 4100 unique locations in 139 countries (Fig. 1). The coverage of biotic Hg tissues concentrations in the GBMS data repository is global and comprises every continent and ocean basin (Table 5). When considering the geographical patterns in ‘risk levels’ it should be noted that the data shown in Fig. 1 represent samples collected over several decades and include many studies specifically focussing on areas with known Hg contamination, which can bias the resulting picture.

**Table 5:** Summary of biotic Hg data from the GBMS database (i.e., number of individuals) by priority taxonomic group identified by the Minamata Convention across oceanic and continental geographies

	Fish	Sea turtles	Birds	Marine mammals	Subtotal
Continental^b^					
Africa	6126	n/a	192	n/a	6318
Antarctica	0	n/a	0	n/a	0
Asia	14,093	n/a	3794	567^a^	18,454
Australia	323	n/a	3	n/a	326
Europe	62,321	n/a	7712	220^a^	70,253
North America	191,346	n/a	50,449	n/a	241,795
South America	38,126	n/a	356	95^a^	38,577
Subtotal	312,335	n/a	62,506	882	375,723
Oceanic					
Antarctic	1228	n/a	6305	1738	9271
Arctic	1808	n/a	7498	8730	18,036
Gulf of Mexico-Caribbean	8480	557	467	818	10,322
Indian	9662	397	1851	487	12,397
Mediterranean	13,720	773	2054	2600	19,147
North Atlantic	26,504	1,438	13,951	6698	48,591
North Pacific	24,049	1,077	33,933	6996	66,055
South Atlantic	12,428	714	2808	1398	17,348
South Pacific	8152	51	3054	351	11,608
Subtotal	106,031	5007	71,921	29,816	212,775
Total	418,366	5007	134,427	30,698	588,498

^a^The marine mammal category also includes seals and dolphins that inhabit freshwater systems

^b^Mercury concentrations in sea turtles and marine mammals are shown within their representative ocean basin instead of continent

Furthermore, the density of datapoints in each region varies greatly and reveals the areas that are less studied than others. The GBMS dataset demonstrates the extent of the global dataset (Table 6) and shows the greatest availability of data at the continental level from North America, Europe, and South America whereas there is less availability from Africa, Antarctica, Asia, and Australia. For ocean basins, the greatest availability of data comes from the Arctic Ocean and Mediterranean Sea, with lesser availability from the Caribbean Sea, Indian Ocean, and North Atlantic and Pacific Oceans and minimal data from the South Atlantic and Pacific Oceans.

**Table 6:**
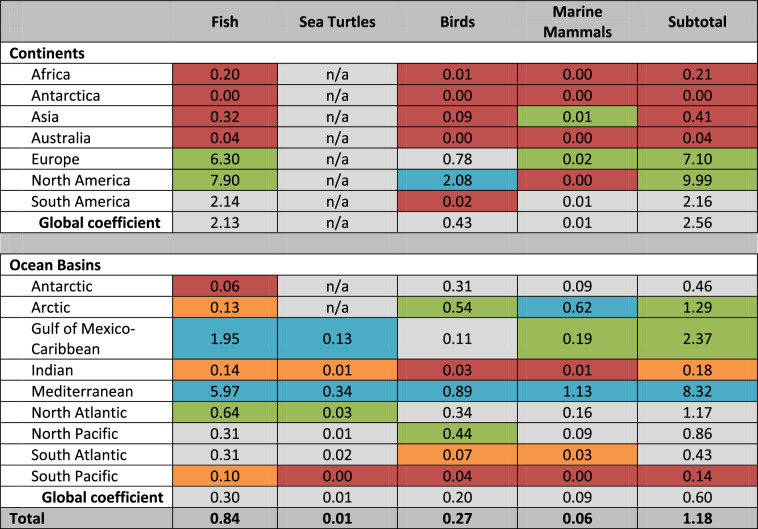
Summary of sampling strength of available biotic Hg data (i.e., [number of individuals/sq. km) × 1000]) by priority taxonomic group identified by the Minamata Convention across oceanic and continental geographies

Global averages are used to categorize relative sampling intensity as very high (>4× above global average in blue), high (2× above global average in green), medium (global average in gray), low (2× below global average in orange) and very low (>4× below global average in red)

The most well-represented species group in the GBMS database are teleosts; bony, ray-finned fishes that are extremely diverse, as they contain over 95% of all fish species and are ubiquitous around the world in freshwater and marine ecosystems. In the GBMS database, marine teleosts were represented in 30 Orders by 92,426 individuals at 826 distinct locations, while freshwater teleosts included 26 Orders with 312,335 individuals at 973 distinct locations. By comparison, cartilaginous fish (elasmobranchs) comprised of sharks, skates, and rays, were represented in 13 Orders by 13,605 individuals at 212 distinct locations. Birds were the second most abundant species group, represented in 26 Orders by 134,427 individuals at 1296 distinct locations. Marine mammals were divided into four groups (i.e., toothed and baleen cetaceans, pinnipeds, and polar bears) and represented by 30,698 individuals at 601 locations. Finally, sea turtles were represented by 5007 individuals from a total of 82 distinct locations.

Many of the data collected reflect the various monitoring programs that exist at local (e.g., New York State, USA), national (e.g., Northern Contaminants Program in Canada), and regional scales (e.g., the Caribbean Regional Mercury Monitoring Network), and even multi-hemispheric scales (e.g., the Arctic Monitoring and Assessment Programme [AMAP]). A summary of these programs is provided based on a review by UNEP (UNEP 2016). In the interest of developing a Hg monitoring network that uses existing Hg data and biomonitoring programs, a framework has been developed for oceans and continents that can draw on the existing Hg data and potentially meet the biomonitoring interests of the Minamata Convention if key geographic and taxonomic data gaps can be filled. These three broad elements are herein covered: (1) biotic data Hg exposure profiles from GBMS, (2) existing Hg monitoring programs, and (3) a path forward for new Hg monitoring frameworks.

To provide sustainable and long-term biomonitoring capacity in key regions around the world where Hg inputs are likely having adverse impacts to human communities and ecological health (e.g., Arctic, tropical areas associated with artisanal small-scale gold mining, and oceanic islands), the focus should be placed on expanding and stabilizing existing national initiatives that use relevant sample sizes that can meet statistical power for confidence in understanding spatial gradients (e.g., ecosystem sensitivity spots; Evers et al. 2011b; Evers et al. 2023) and temporal trends (Bignert et al. 2004; Rigét et al. 2011; Braaten et al. 2019; Morris et al. 2022a). Moreover, it is crucial to foster international collaboration and coordination among national or local projects to create harmonized regional approaches, and to strive, where possible, to integrate biomonitoring activities into a standardized framework to properly assess regional and global spatiotemporal patterns of risk to human and environmental health.

The GBMS database and associated peer-reviewed publications provide a platform to assess broad spatial scales of Hg tissue concentrations in key food items related to human health for general (e.g., tuna, swordfish) and regional fish populations (e.g., sharks, freshwater fish), Indigenous Peoples (marine and freshwater fish, toothed whales, pinnipeds) and subsistence communities (which can include all the major taxa of concern). The health of ecosystems can also be viewed through bioindicators that are not necessarily key food items but are representative of taxa where the literature is robust (e.g., sea turtles, seabirds, loons, raptors, freshwater birds, landbirds, and marine mammals). We begin each section with a brief rationale for why each taxonomic group is important for Hg biomonitoring and then discuss associated caveats. We include data from the GBMS database, which summarizes Hg data from over 1700 peer-reviewed publications, to demonstrate the breadth of biotic tissue Hg data availability (spatially and temporally) and to better understand local, regional, and global patterns that can be used as a beginning point for identifying data and knowledge gaps for effectiveness evaluation purposes of the Minamata Convention.

Below, biotic data are organized by 1) Human exposure bioindicators - those organisms which are consumed by humans and may potentially pose a risk to human health; and 2) Ecological health bioindicators – those organisms that best represent Hg impacts to ecological health (Table 1).

### Human exposure bioindicators

Many Indigenous Peoples in remote places depend on their local ecosystems for sustenance. For example, Arctic Indigenous Peoples rely on access to their traditional country foods for food security, for their general health and well-being, and as part of their spiritual and cultural identity, among many other things (AMAP 2021; Basu et al. 2022). However, due to exposure through the diet, Arctic Indigenous Peoples can experience some of the highest Hg levels globally (Basu et al. 2018, 2023). In such situations, good risk communication is essential to ensure that the proper messages are conveyed in balancing the risks associated with Hg exposure against the nutritional and cultural benefits of traditional diets.

In many other parts of the world, communities depend in part, and sometimes completely, on wild animals for subsistence. The following section describes known Hg concentrations for a broad range of biota and geographic areas. Specifically highlighted with data summaries are: (1) high trophic level marine fish that are widespread across the world’s oceans: tuna, billfish, and sharks; (2) the Caribbean and Mediterranean Seas; (3) freshwater fish within six continents; (4) seabirds and waterfowl in subarctic marine systems; and (5) marine mammals (e.g., toothed whales in the northern oceans). Due to the importance of dietary Hg exposure and the global impact on human health, patterns depicting the interaction of dietary MeHg uptake in humans are herein described for all the world’s major biomes from the Arctic and subarctic to temperate and tropical aquatic ecosystems. Ingesting elevated fish muscle Hg concentrations, such as in sharks, can exceed commonly suggested reference concentrations in less than two weeks (Baek et al. 2023). Often, biotic Hg concentrations can be linked to anthropogenic Hg point sources, such as ASGM activities, which have been connected to elevated Hg levels in nearby communities (Gibb and O’Leary 2014; Basu et al. 2023).

While other environmental (e.g., contaminant mixes; Alves et al. 2022), micronutrient (e.g., selenium; Lailson-Brito et al. 2012; Gochfeld and Burger 2021; Storelli et al. 2022; Sabino et al. 2022), and nutritional factors (e.g., omega-3s; Sardenne et al. 2020) clearly can confound assessments of Hg on human health those costs and benefits are not evaluated herein. The following biotic groups illustrate how biotic Hg exposure can be linked to human exposure concerns in several key ecosystems in the world using select bioindicators.

#### Marine fish - tuna

##### Rationale and caveats for Hg biomonitoring

**Fig. 2:**
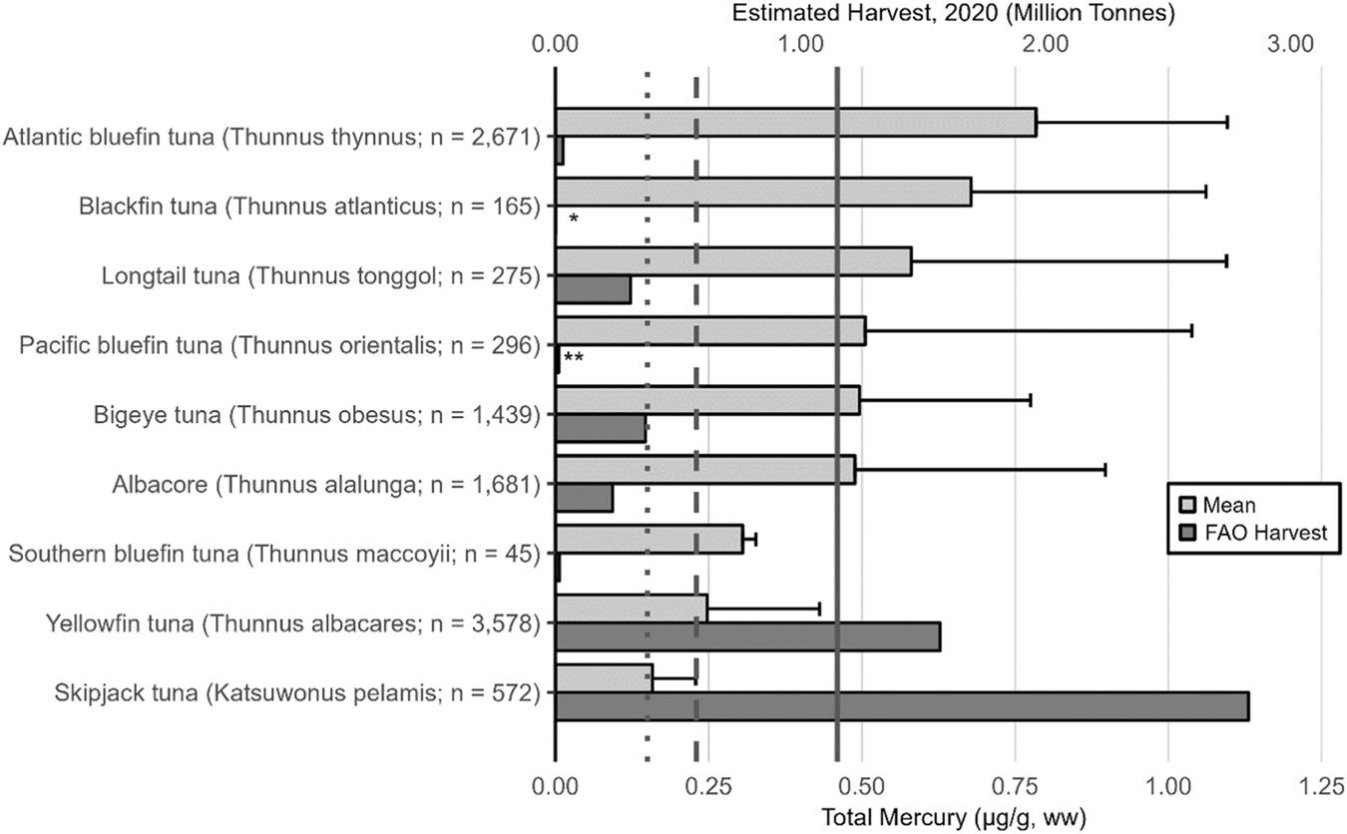
Mercury concentrations in nine species of tuna and their associated global harvest. Light gray bars represent the arithmetic mean ± SD of total Hg concentrations (µg/g, ww) in muscle tissue. Dark gray bars show FAO harvests estimates (in tonnes). Tuna with harvests of <15,000 tonnes are depicted with “**” while tuna with harvest of <5000 tonnes are depicted with “*”. Data are not normalized by size. Canned tuna Hg data are not included here. USEPA-USFDA human health thresholds for Hg consumption (µg/g, ww) are shown as dotted (0.15) dashed (0.23) and solid (0.46) lines (see Table 2 for consumption guidelines)

Tuna species are one of the most important global sources of seafood and inhabit broad areas the Atlantic, Pacific, and Indian Oceans. Commercial harvests tracked by the Food and Agriculture Organization (FAO) for the seven most commercially available species totaled 5.2 million metric tons in 2018, worth an estimated value of $41 billion; this does not include substantial value associated with subsistence and artisanal fisheries and sport fisheries (McKinney et al. 2020). Projections indicate that the global market may reach over $50 billion by 2028. However, sustainably managing tuna fisheries to allow depleted stocks to recover has been challenging and generally does not account for the adverse impacts of MeHg tissue concentrations that may reduce reproductive output and growth rates. Excessive fishing pressure continues to threaten tuna stocks of eastern Pacific yellowfin, Pacific bluefin, Atlantic bigeye, Indian Ocean yellowfin, and southern bluefin. Mercury biomonitoring deliberations should consider tracking Hg concentrations in all nine of the tuna species that average or range above the 0.22 µg/g, ww threshold of “a two-meal limit/week” (see Table 2 for human meal frequency and Fig. 2 for the Hg profile) and biomonitoring considerations should account for species differences, size classes, changes in stock abundance from overfishing, differences in foodweb structure, and size of home range (Schartup et al. 2019).

The GBMS database includes 10,722 Hg concentrations of 9 species representing 120 publications. Muscle Hg concentrations and commercial harvest vary widely by species. The smallest tuna species (e.g., skipjack tuna, *Katsuwonus pelamis*) has average Hg concentrations under the USEPA-USFDA advisory level of 0.23 µg/g, ww while the largest (e.g., Pacific and Atlantic bluefin tunas, *Thunnus orientalis and T. thynnus*, respectively) have the highest average Hg concentrations and often exceed advisory levels (Fig. 2).

These patterns vary by size class within species and ocean basin origin. For example, whereas yellowfin tuna (*Thunnus albacares*) tends to have lower average muscle Hg concentrations than seven of the nine tuna species with data (Fig. 2) larger individuals (e.g., weighing over 70 kg) typically have Hg concentrations that are of human health concerns (Bosch et al. 2016a). Yellowfin, bigeye tuna (*Thunnus obesus*), and albacore tuna (*Thunnus alalunga*) Hg concentrations grouped by major ocean basin indicates that the eastern and northern areas of the Pacific Ocean have significantly higher Hg concentrations than other ocean basins (Ferriss and Essington 2011; Nicklisch et al. 2017; Houssard et al. 2019; Médieu et al. 2021). This area of the Pacific Ocean is where increasing tuna Hg concentrations have been recorded over the past decade (Drevnick et al. 2015; Drevnick and Brooks 2017) and modeled for several decades into the future (Sunderland et al. 2009). Tuna Hg concentrations in other ocean basins are known to be decreasing (North Atlantic Ocean; Lee et al. 2016) or remaining stable (southwestern Pacific Ocean; Médieu et al. 2021). As well as size and origin, other interpretive factors to consider include whether the tuna is canned or fresh (for the same species; canned tuna tend to have lower Hg concentrations; García et al. 2016) and farmed vs. wild. Although farmed tuna tend to have lower Hg concentrations (Balshaw et al. 2008; Annibaldi et al. 2019), the amount of Hg bioaccumulation in muscle tissue in wild-caught, pen-raised tuna depends on time spent in rearing pens (Srebocan et al. 2007).

#### Marine fish - billfish

##### Rationale and caveats for biomonitoring

**Fig. 3:**
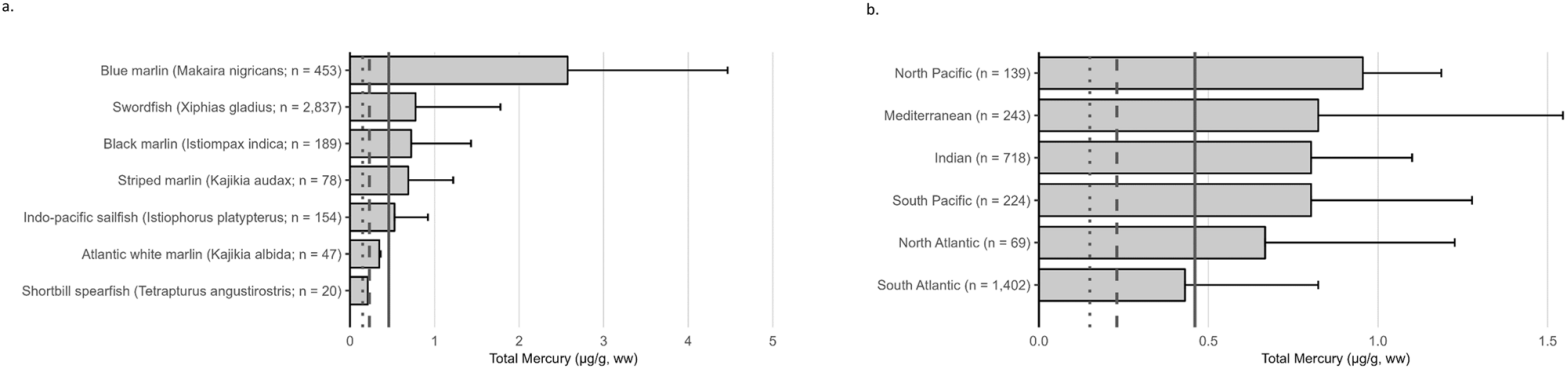
**a** Mercury concentration in seven species of billfish. Gray bars illustrate the arithmetic mean ± SD of total Hg concentrations (µg/g, ww) in dorsal muscle tissue. USEPA-USFDA human health thresholds for Hg consumption (µg/g, ww) are shown as dotted (0.15) dashed (0.23) and solid (0.46) lines (see Table 2 for consumption guidelines). **b** Mercury concentrations in swordfish. Gray bars illustrate the arithmetic mean ± SD of global total Hg concentrations (µg/g, ww) in dorsal muscle tissue of swordfish from six ocean basins. USEPA-USFDA human health thresholds for Hg consumption (µg/g, ww) are shown as dotted (0.15) dashed (0.23) and solid (0.46) lines (see Table 2 for consumption guidelines)

Large and relatively long-lived pelagic species such as billfishes can be used as bioindicators for understanding expansive spatial gradients of MeHg contamination in the world’s oceans using current commercial resources. Mercury concentrations in billfish, such as marlin (multiple genera; Drevnick and Brooks 2017, Vega-Sánchez et al. 2017, Bille et al. 2020, Rudershausen et al. 2023) and swordfish (*Xiphias gladius*, Mendez et al. 2001, Branco et al. 2007), are some of the highest known for marine teleost fish (Rodrigues and Amorim 2016) and adverse impacts to their physiology and body condition may be of concern for some populations (Biton-Porsmoguer et al. 2022). Swordfish are the most widespread of the billfish and northern hemisphere stocks are generally managed sustainability (western, central, and eastern North Pacific and North Atlantic stocks) (National Oceanic Atmospheric Administration, Department of Commerce unpubl. data) and provide a long-term opportunity for broad geographic and robust sampling options. Southern hemisphere swordfish stocks are less understood and in the case within the Indian Ocean are declining. Mercury biomonitoring deliberations should consider tracking Hg concentrations in swordfish, which average above the 0.46 µg/g, ww threshold of “choices to avoid” (see Table 2 for human meal frequency and Fig. 3a for the Hg profile) and biomonitoring considerations should account for differences among billfish species, size classes, changes in stock abundance from overfishing, differences in foodweb structure, and size of home range.

The GBMS database includes over 3778 Hg concentrations of seven billfish species representing 54 publications. Of the billfish, the highest average Hg concentrations are in blue marlin (*Makaira nigricans*), nearly 4× global averages of the swordfish (Fig. 3a). In swordfish, Hg tissue concentrations vary according to major ocean basin with a tendency for a doubling of Hg concentrations in the Northern Hemisphere compared to the Southern Hemisphere (Fig. 3b; 0.79 ± 0.52 µg/g, ww and 0.54 ± 0.42 µg/g, ww, respectively). Elevated Hg levels in swordfish are to be expected because of their high trophic level and relatively long lifespan (>10 years). As these data illustrate, swordfish often exceed human health thresholds (see Fig. 3b vertical lines – only the South Atlantic population has mean levels below the “do not eat” threshold), making their consumption a human health concern. However, swordfish have important commercial value and are an important source of income for many oceanic island communities.

The Indian Ocean is a good case study where approximately 30,000 tonnes of swordfish are harvested annually (i.e., 25% of annual global catch during 2016–2018), half of it being caught by fleets of Indian Ocean coastal countries (FAO 2018; IOTC 2020). Sri Lanka, India, and Seychelles fisheries are the main contributors accounting for 24%, 10% and 8% of the annual total catch of swordfish in the Indian Ocean, respectively, contributing mostly to the global export market. Exports/imports of fish products are however strictly monitored when it comes to fish Hg content particularly for Europe, which is the top importing market for swordfish (FAO 2018). Indeed, the EU requires predatory pelagic fish (e.g., tuna and swordfish) imports to have <1.0 µg/g, ww of Hg for human consumption (EU Commission 2006). Total Hg concentrations in swordfish have been well investigated since the 2000s in the Indian Ocean, highlighting variable Hg concentrations depending on the swordfish size/age (largest and oldest swordfish having the highest Hg levels), and the area where it was caught. Overall, higher Hg concentrations were recorded in swordfish from the Western Indian Ocean compared to the Eastern Indian Ocean (Hg: 0.9 ± 0.1 and 0.6 ± 0.1 µg/g, ww, respectively) (Esposito et al. 2018), and from the southern Indian Ocean compared to the central-northern Indian Ocean (Hg: 2.0 ± 0.1 and 0.9 ± 0.1 µg/g, ww, respectively) (Sabino et al. 2022).

Approximately 13%, 13% and 43% of swordfish caught from the Sri Lanka, Seychelles and Reunion waters, respectively, were found to exceed the EU advisory level (Hollanda et al. 2017; Jinadasa and Fowler 2019; Kojadinovic et al. 2006). Swordfish with concentrations over this EU advisory level are not permitted for export to the EU. These large, high commercial value specimens therefore must either remain within island communities or are exported to other countries for less value. Thus, high Hg concentrations in fish can result in significant adverse economic, ecological and human health impacts, especially in the case of Small Island Developing States that rely highly on their fisheries such as the Seychelles (Bistoquet et al. 2018). Indeed, the last EU ban on Seychelles swordfish exports (2014) led the Seychelles longline fleet to favor exports of large tropical tunas over swordfish, as Hg concentrations in the tuna species within the central-western Indian Ocean are generally below 0.5 µg/g, ww (Bodin et al. 2017). However, this may not be a long-term solution due to the declining status of tuna populations in the Indian Ocean (e.g., yellowfin tuna: overfished and subject to overfishing) (IOTC 2018).

#### Marine fish – sharks

##### Rationale and caveats for biomonitoring

**Fig. 4:**
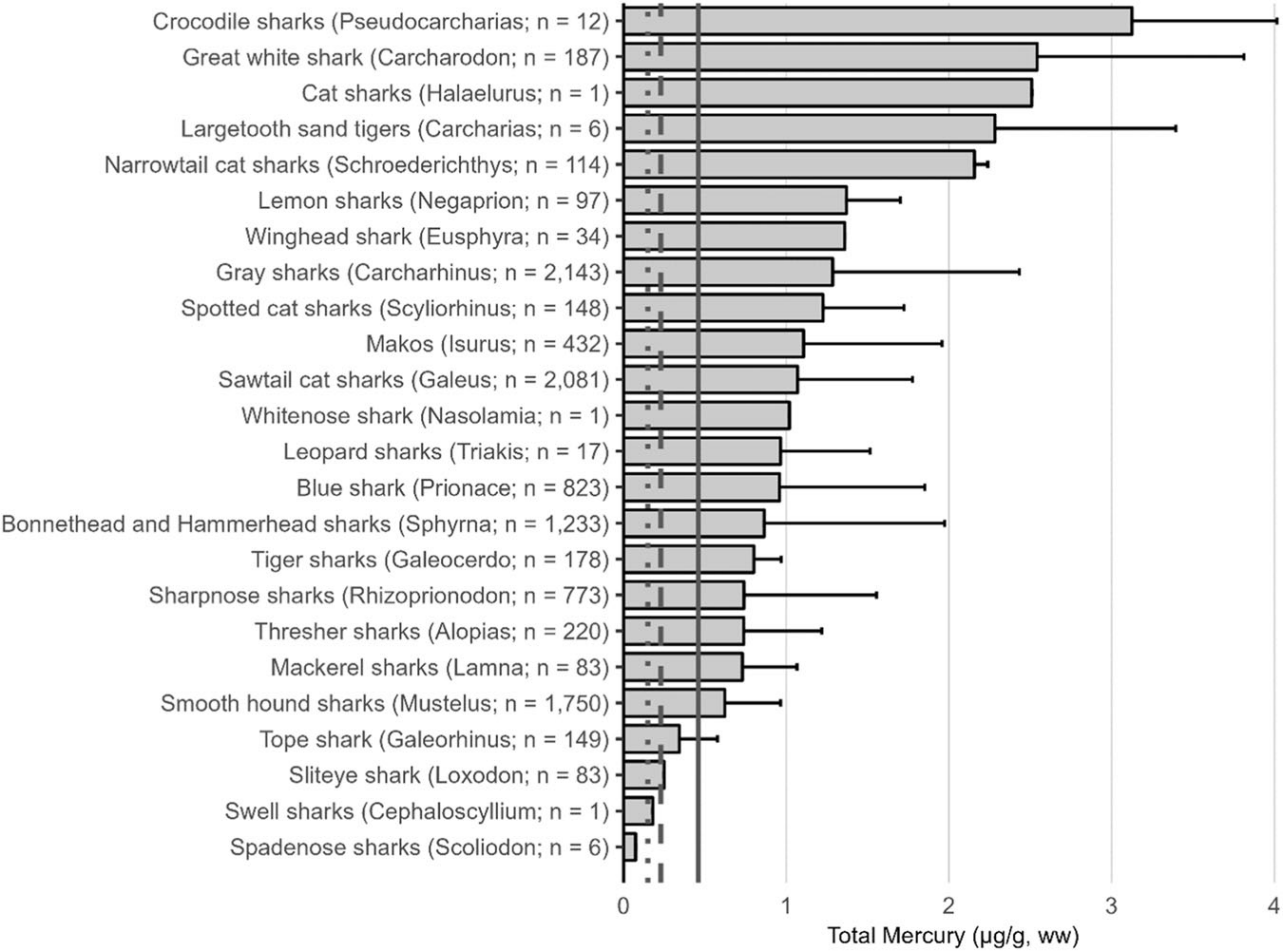
Mercury concentrations in sharks. Gray bars illustrate the arithmetic mean ± SD of global total Hg concentrations (µg/g, ww) in muscle tissue of sharks by genus (24 genera) from the Orders of Mackerel and Ground Sharks from the GBMS database. USEPA-USFDA human health thresholds for mercury consumption (µg/g, ww) are shown as dotted (0.15) dashed (0.23) and solid (0.46) lines (see Table 2 for consumption guidelines)

Sharks are a diverse and important group of marine species, as there are over 470 species of sharks that are defined within eight Orders in the subclass Elasmobrachii (i.e., elasmobranchs, which include sharks, skates, and rays), containing several top predators that are known to have cascading impacts on ecosystems they inhabit (Hammerschlag et al. 2019, 2022). Sharks are an important source of food in many cultures, and have been severely overexploited, with many species facing high extinction risk (Gallagher et al. 2012; Pacoureau et al. 2021; Sherman et al. 2023; Worm et al. 2024). Most shark species are known to contain elevated muscle Hg concentrations and their use as top trophic level bioindicators for marine ecosystems is well established. Mercury biomonitoring deliberations should consider tracking Hg concentrations in the 21 of 24 (88%) shark genera that average or range above the 0.46 µg/g, ww threshold of ”choices to avoid” (see Table 2 for human meal frequency and Fig. 4 for the Hg profile) and biomonitoring considerations should account for species differences, size classes, knowledge of prey availability, foraging depth (that can be measured with stable isotopes), and size of home range. Shark fins can be used as an indicator of Hg exposure (Kim et al. 2016; Vélez et al. 2021).

The GBMS database shows that species within two of the eight Orders, the mackerel (Order Lamniformes) and ground (Order Carcharhiniformes) sharks generally have tissue Hg concentrations well above the human health advisory levels of no consumption set by the USEPA (0.46 µg/g, ww) and World Health Organization (1.0 µg/g, ww; Fig. 4). Implications of these elevated Hg tissue concentrations are also of concern for overall shark health, which adds to population stresses due to overfishing. Many species are on the IUCN Red List of Threatened Species and are overfished for their fins and meat.

The GBMS database includes 10,578 Hg concentrations of 24 genera of sharks. Many of the measurements are from blue (*Prionace glauca*), mako (*Isurus* spp.), great white (*Carcharodon carcharias*), hammerhead (*Sphyrna* spp.), silky (*Carcharhinus falciformis)*, bull (*Carcharhinus leucas*), lemon (*Negaprion brevirostris*), and porbeagle (*Lamna nasus*) sharks, where large individuals well exceed human health advisory standards (Branco et al. 2004, 2007; Escobar-Sánchez et al. 2011; Maz-Courrau et al. 2012; de Carvalho et al. 2014; McKinney et al. 2016; Nicolaus et al. 2016; Matulik et al. 2017; Biton-Porsmoguer et al. 2018; Terrazas-López et al. 2019; Rodriguez-Gutiérrez et al. 2020; Maurice et al. 2021; Erasmus et al. 2022a, 2022b; Riesgo et al. 2023). Pelagic foraging piscivorous species tend to have higher Hg tissue concentrations compared to those foraging in benthic habitats and on invertebrates (de Pinho et al. 2002; Matulik et al. 2017). Further, the mesopelagic zone may be an important entry point for MeHg into the foodweb (Choy et al. 2009) – a zone that provides over 70% of the prey for larger species, such as the great white sharks (*Carcharodon carcharias*) in the northeastern Pacific Ocean (Le Croizier et al. 2020). Ultimately, trophic level of prey dictates muscle MeHg concentrations in sharks (Le Croizier et al. 2022b), but distribution of prey species in the ocean water column may also be an important factor (Choy et al. 2009; Furtado et al. 2021).

Of the 24 shark genera with published muscle Hg concentrations, the GBMS data shows that average levels exceed the USEPA human health standards of 0.46 µg/g, ww for 83% of genera and the WHO and EU standard of 1.0 µg/g, ww in 50% of genera (Fig. 4). Mercury concentrations are similar in all muscle tissue including fins (O’Bryhim et al. 2017; Kim et al. 2016), which are often also used as a basis for soup (Barcia et al. 2020). This is noteworthy given the practice of shark fin soup consumption in Asia (Worm et al. 2024).

Despite having among the highest levels of Hg recorded in any vertebrate, the physiological and behavioral effects of Hg concentrations on elasmobranchs remains largely unknown (Wosnick et al. 2023). Although chronic dietary MeHg uptake of 0.2 µg/g, ww in freshwater fish have been found to affect reproduction and other subclinical endpoints (Depew et al. 2012a), studies on the effects of MeHg in the shark brain indicate abilities to demethylate (Ehnert-Russo and Gelsleichter 2020) and potentially use detoxifying mechanisms through selenium-Hg liaisons (Branco et al. 2007; Dutton and Venuti 2019; Medina-Morales et al. 2020) or other physiological abilities (Le Croizier et al. 2020). For example, while Merly et al. (2019) found that blood concentrations of Hg in white shark (*Carcharodon carcharias*) exceeded levels that are known to be toxic to humans, no negative effects on shark health parameters were detected, including body condition, total leukocytes, or granulocyte to lymphocyte ratios. The authors speculated that sharks may have protective mechanisms that mitigate harmful effects of heavy metal exposure. However, only circulating blood Hg concentrations were measured, which may be more transient and less likely to impact shark health. Conversely, Wosnick et al. (2021) found that Hg concentrations in hepatic and gill tissues of sharks were associated with increased activity of alkaline phosphatase and deregulation of urea and lactate markers, respectively. The former relationship suggests possible alterations in liver-kidney functioning from Hg toxicity, while the later association suggests potential compromised gill functioning in osmoregulation. Clearly, there is a need to better understand the effects of Hg exposure on elasmobranch fitness and survival.

In addition to high Hg concentrations, as long-lived and high trophic level generalist species, sharks are prone to bioaccumulation and biomagnification of various heavy metals and other toxins (e.g., Hammerschlag et al. 2016; Shipley et al. 2021), which may additively or synergistically impact shark health and survival.

#### Marine fish – Mediterranean Sea

##### Rationale and caveats for biomonitoring

The Mediterranean Sea is a semi-enclosed area characterized by strong North-South and West-East gradients of environmental conditions with a residence time of waters of approximately a century (Millot and Taupier-Letage 2005). It covers an area of about 2,500,000 km^2^ (970,000 mi^2^) and has an average depth of 1500 m (4900 ft) with the deepest point at 5267 m (17,280 ft) in the Ionian Sea. Total captured fisheries production in the Mediterranean and Black Seas peaked in 1988 at approximately 1.8 million tonnes and although this has since fallen to around 1.2 million tonnes/yr for the period 2018–2022 (FAO 2020, 2022) fish stocks are still an important food source for local communities. Herrings, sardines, and anchovies accounted for 56% of the total landings with a mean annual amount over 665,000 tonnes, followed by miscellaneous coastal fishes (10%, 117,300 tonnes), and miscellaneous pelagic fish such as cods, hakes, and haddocks (10%, 123,500 tonnes). Catches of small pelagic species presented large fluctuations during this period linked to the variability of environmental factors, while decreasing landings were observed for some demersal species (European hake, whiting, turbot and sole) and increasing landings for a few other ones (red mullet, surmullet and blackspot seabream) (FAO 2022).

**Fig. 5:**
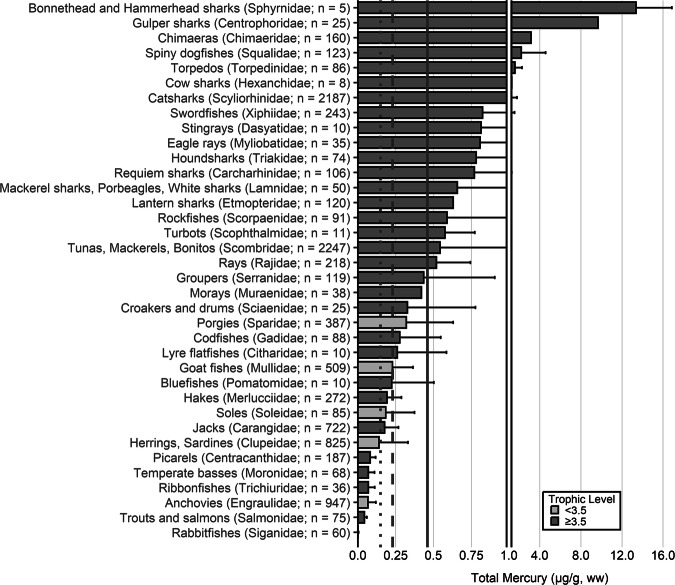
Mercury concentrations in Mediterranean Sea fish (including estuarine species). Gray bars illustrate the arithmetic mean ± SD of global total Hg concentrations (µg/g, ww) in muscle tissue of 36 fish families from the GBMS database representing the Mediterranean Sea. USEPA-USFDA human health thresholds for Hg consumption (µg/g, ww) are shown as dotted (0.15) dashed (0.23) and solid (0.46) lines (see Table 2 for consumption guidelines). The double line is a break in the x-axis to better depict and view lower Hg concentrations

In the Mediterranean Sea, the Western Mediterranean continues to be the most productive area, accounting for 20% of the total landings, followed by the Eastern Mediterranean (15%), the Adriatic (14%) and the central Mediterranean (14%), while the Black Sea provided 38% of the total catch with 446,067 tonnes during the 2018–2020 period. Mercury biomonitoring deliberations should consider tracking fish Hg concentrations in the 24 of 36 (67%) fish families that average or range above the 0.46 µg/g, ww threshold of “choices to avoid” (see Table 2 for human meal frequency and Fig. 5 for the Hg profile) and account for differences in species distributions and abundance, as well as location within the Mediterranean Sea.

The GBMS dataset for the Mediterranean Sea includes 13,720 Hg concentrations for 111 fish species in 58 families – including 36 focal families– based on 59 publications (Fig. 5). These findings indicate several families of sharks – including Bonnethead/Hammerhead sharks (Sphyrnidae; x = 13.4 ± 3.5; n = 5), Gulper sharks (Centrophoridae; x = 9.66 µg/g; n = 25), Chimaeras (Chimaeridae; x = 3.14 µg/g; n = 160), Spiny dogfishes (Squalidae; x = 2.2 ± 2.4 µg/g; n = 123) - have the highest Hg concentrations for this region. Swordfish and tunas (*Thunnus and Katsuwonus*) have some of the highest Hg concentrations in bony fishes and average well above the USEPA safety threshold level for human exposure in sensitive populations (0.15 µg/g, ww). While, Indigenous Peoples from the Amazon and the Arctic have been found to have the highest Hg levels globally (Basu et al. 2018, 2023), non-indigenous people living in the Mediterranean region have the second highest Hg levels (Petrova et al. 2020). Most marine fish from the Mediterranean Sea have average Hg concentrations that are restrictive for safe human consumption (Cinnirella et al. 2019).

However, the lowest trophic level fish species (e.g., those that depend on zooplankton as primary prey) including herring, sardines, anchovies, and picarels, that accounted for more than half the total landings, have the lowest average Hg concentrations and are generally safe for human consumption (Fig. 5). Higher Hg concentrations of Mediterranean fish are generally recorded in larger/older individuals than in smaller/younger ones, in high trophic level predators than in low trophic level herbivores and zooplanktivores, in benthic than in pelagic species, in deeper than shallower environments, and in oligotrophic than mesotrophic waters (Cresson et al. 2014, 2015; Maulvault et al. 2016; Chouvelon et al. 2018; Sánchez-Muros et al. 2018). This explains why the short-lived pelagic zooplanktivores (Engraulidae and Clupeidae) exhibited Hg concentrations lower than the minimum USEPA threshold (<0.16 µg/g, ww), while deep demersal families (Scorpaenidae) and large, long-live pelagic predators (Xiphiidae and Scombridae) presented much higher Hg concentrations (>0.60 µg/g, ww) (Biton-Porsmoguer et al. 2022), that are restrictive for human health (Fig. 5). Demersal and deep sharks and rays also presented very high Hg concentrations in the Mediterranean Sea (Storelli et al. 2002).

Several studies have shown that Mediterranean fish species have higher concentrations of Hg in their tissues than the same species from the Atlantic Ocean (e.g., Renzoni et al. 1998; Cossa et al. 2012; Cransveld et al. 2017; Cammilleri et al. 2018; Chouvelon et al. 2018; Di Bella et al. 2018; Mauffret et al. 2023). More generally, high concentrations of Hg have been observed in Mediterranean predatory organisms, likely because the Mediterranean is one of the places in the World Ocean where Hg methylation potential is the highest (Cossa and Coquery 2005). The high Hg-enrichment in Mediterranean fish compared to other regions at the same latitudes results from the synergy of several factors: (1) the shallower location of the MeHg maximal concentration in the water column that induces a higher MeHg transfer into the biota, (2) the slower growth rates of fishes resulting in a higher age-at-length that induces a longer exposure to Hg at a given length, (3) higher concentrations in zooplankton, and (4) longer food webs linked to oligotrophic conditions and small sizes of phytoplankton cells (Buckman et al. 2018; Cossa et al. 2022).

The synergy of environmental and biological factors induces a high spatial variability in Hg concentrations of Mediterranean fishes (Cinnirella et al. 2019), exemplified here by the high standards deviations on Hg concentration means in Fig. 5. At the basin level, fishes from the Western Mediterranean appear more contaminated than those from the Eastern Mediterranean, in relation to higher MeHg concentrations in the Western basin waters (Cossa et al. 2022). At a regional scale, the areas of particular concern are the North of the Western basin (Cresson et al. 2014), the Adriatic Sea (Storelli et al. 2005; Grgec et al. 2020), the Tyrrhenian Sea (Buckman et al. 2018), some places in the Ionian Sea (Signa et al. 2017) and the Sea of Marmara (Keskin et al. 2007), while lower concentrations are reported from fishes from the Aegean Sea (Kucuksezgin et al. 2001), the Black Sea (Harmelin-Vivien et al. 2009) and the coast of Tunisia (Joiris et al. 1999).

#### Marine fish – Caribbean Sea

##### Rationale and caveats for biomonitoring

**Fig. 6:**
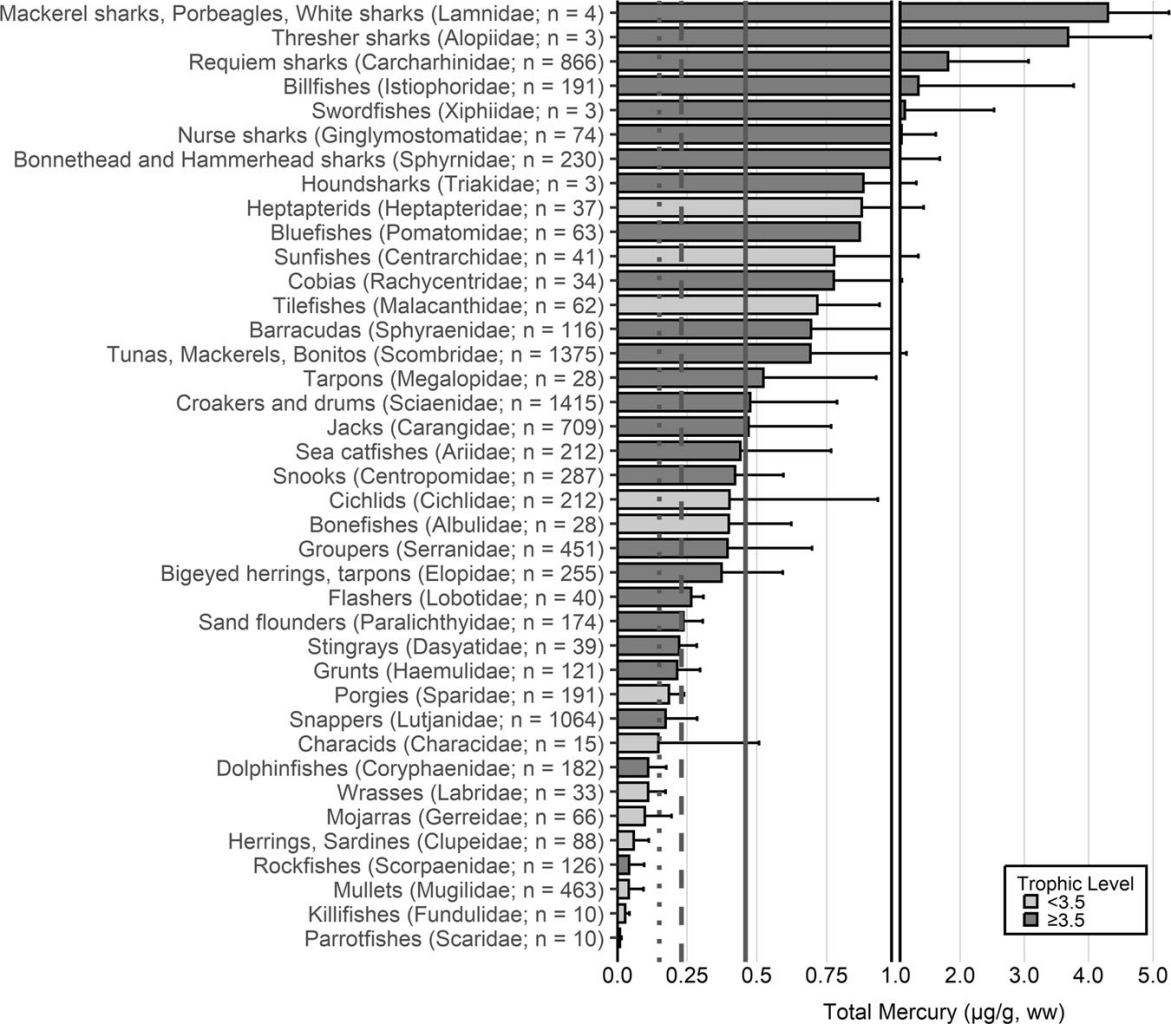
Mercury concentrations in Caribbean Sea fish (including estuarine species). Gray bars illustrate the arithmetic mean ± SD of total Hg concentrations (µg/g, ww) in muscle tissue of 39 fish families from the GBMS database that represent the Caribbean Sea. USEPA-USFDA human health thresholds for Hg consumption (µg/g, ww) are shown as dotted (0.15) dashed (0.23) and solid (0.46) lines (see Table 2 for consumption guidelines). The double line is a break in the x-axis to better depict and view lower Hg concentrations

The Caribbean Sea includes numerous islands of the West Indies, and adjacent coasts of North and South America and has an area of about 2,754,000 km^2^ (1,063,000 mi^2^). The Sea’s deepest place is the Cayman Trough, between the Cayman Islands and Jamaica, at 7686 m (25,217 ft) below sea level. The Caribbean Sea has the world’s second largest barrier reef, the Mesoamerican Barrier Reef. It extends over 1000 km along the coasts of Mexico, Belize, Guatemala, and Honduras. The area generates a relatively robust fishing industry, accounting for 500,000 tonnes of fish a year (FAO 2018). Mercury biomonitoring deliberations should consider tracking fish Hg concentrations in 25 of 39 (64%) fish families that average or range above the 0.46 µg/g, ww threshold of “choices to avoid” (see Table 2 for human meal frequency and Fig. 6 for the Hg profile) and account for differences in species distributions and abundance, as well as location within the Caribbean Sea.

A new monitoring effort, the Caribbean Region Mercury Monitoring Network, is now established with the laboratory hub in Antigua and Barbuda. The Network has selected key bioindicators of Hg for purposes that meet economic, human health safety, and logistical reasons for long-term Hg monitoring. The focal species include three that have average Hg concentrations below 0.22 µg/g, ww and have important local and commercial importance: yellowfin tuna (*Thunnus albacares*), red snapper (*Lutjanus campechanus*) and mahi-mahi (*Coryphaena hippurus*). Other species such as the great barracuda (*Sphyraena barracuda*) routinely have elevated Hg concentrations but are not as regularly consumed because of ciguatera fish poisoning concerns (Chinain et al. 2021). There are also multiple grouper species that are of local economic interests and should have regular monitoring of their Hg concentrations – species, size class and location are important factors for interpretation (Sinkus et al. 2021; Christian et al. 2024). For teleost fish, large pelagic species are generally of greater concern to human health than small pelagic and reef species (Shrestha et al. 1988; Ricketts et al. 2016). All shark species have mean Hg concentrations that are well above human health standards (Fig. 6) and especially for some areas such as in Trinidad and Tobago (Mohammed and Mohammed 2017).

The GBMS dataset for fish (elasmobranchs and teleosts) for the Caribbean Sea includes 8,480 Hg concentrations for 193 species in 67 families – including 39 families of greatest interest from 26 publications (Fig. 6). The findings indicate that mackerel sharks/Porbeagles/white sharks (Lamnidae; 4.3 ± 0.95 µg/g, ww; n = 4), thresher sharks (Alopiidae; x = 3.68 ± 1.29 µg/g, ww; n = 3), requiem sharks (Carcharhinidae; x = 1.81 ± 1.25 µg/g, ww; n = 866), billfish (x = 1.35 ± 2.42 µg/g, ww; n = 191) and swordfish (x = 1.14 ± 1.39 µg/g, ww; n = 3) have the highest Hg concentrations for this region. Species that have the lowest risk of Hg contamination to people include mahi-mahi, herring, sardines, lionfish (in the family Scorpaenidae), and mullets (Fig. 6: Adams 2009; Ahmed et al. 2020; Acosta-Coley et al. 2023). As part of the now established Caribbean Region Mercury Monitoring Network, a more recent analyses of over 1600 fish muscle samples for total Hg found a lower ratio of 26% of species exceeding the 0.46 µg /g, ww guideline (although few sharks and billfish were included) (Christian et al. 2024).

Areas of particular concern, which often times are related to Hg sources in the watersheds that flow into Caribbean Sea, include deltas, mangroves, and nearshore marine waters from ASGM activities in Suriname (Mol et al. 2001), from chlor-alkali facilities in Colombia (Alonso et al. 2000; Olivero-Verbel et al. 2008; Gallego Ríos et al. 2018) or other less defined sources such as long-distance transport (Guzmán and García 2002). Based on Hg concentrations in barred grunt (*Conodon nobilis*) from Trinidad and Tobago, levels are generally highly elevated in the Gulf of Paria and the Colombus Channel and could be related to river runoff with Hg from ASGM activities in countries of northern South America and carried towards Trinidad by the Guiana Current (Christian et al. 2024).

#### Freshwater fish - Africa

##### Rationale and caveats for biomonitoring

The major river basins of Africa include the Nile (~6700 km or 4160 miles), the Congo (~4670 km or 2900 miles), the Niger (~4170 km or 2590 miles), and the Zambesi (~2740k or 1700 miles), while the largest lakes include Lakes Victoria, Tanganyika, and Malawi. These and other areas have nearly four million people engaged in fishing-related activities (Heck et al. 2007) and for some countries provide up to 70% of their animal protein (FAO 2012; Hanna et al. 2015). Whereas industrial Hg releases are relatively small in Africa (with the exception of coal-fired power plants in South Africa), approximately 70% of the estimated total Hg emissions and releases are associated with artisanal and small-scale gold mining (ASGM; UNEP 2019a).

**Fig. 7:**
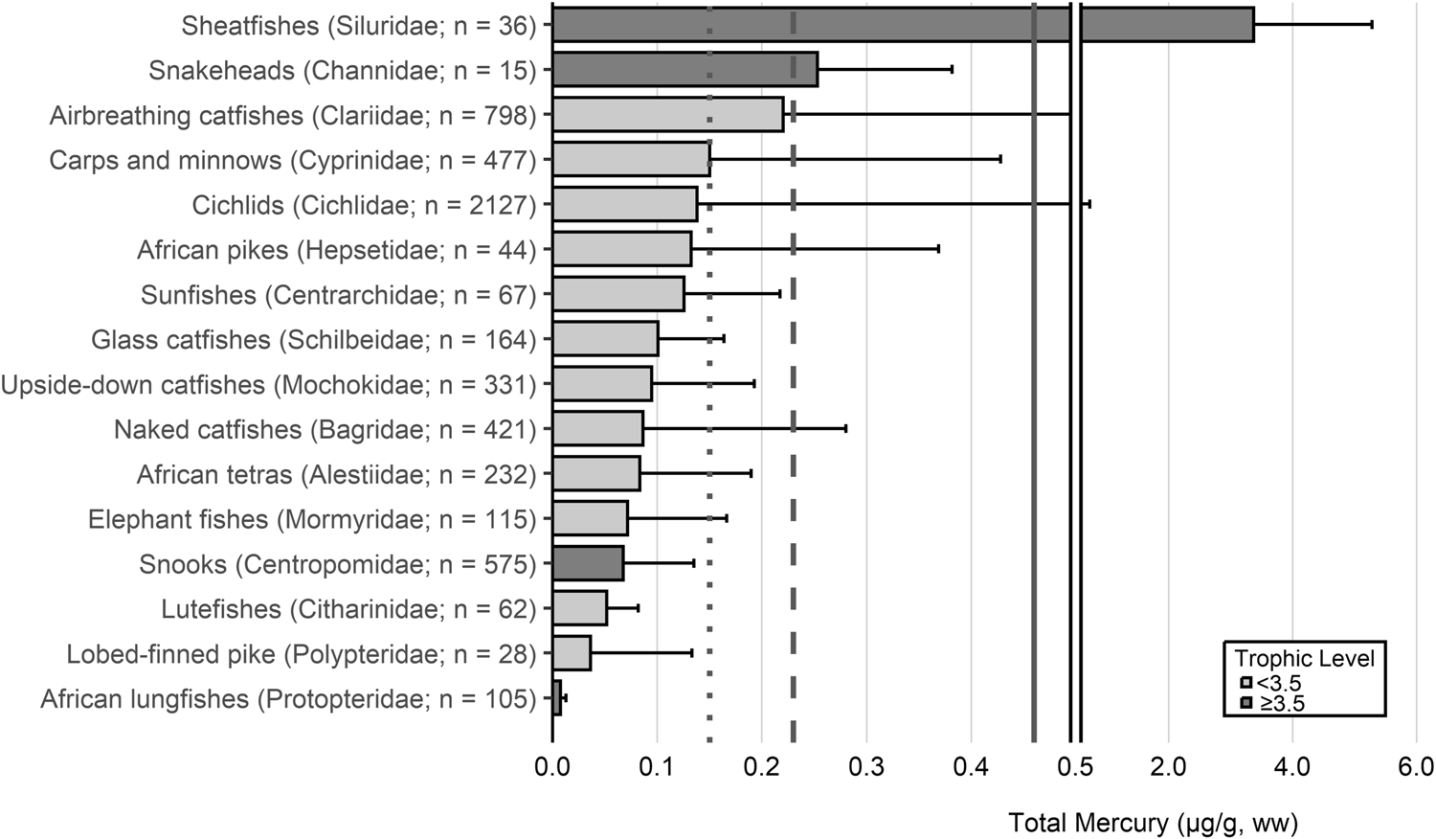
Mercury concentrations in freshwater fish in Africa (including estuarine species). Gray bars show the arithmetic mean ± SD of total Hg concentrations (µg/g, ww) in muscle tissue of 16 teleost fish families selected from the GBMS database that represent Africa. USEPA-USFDA human health thresholds for Hg consumption (µg/g, ww) are shown as dotted (0.15) dashed (0.23) and solid (0.46) lines (see Table 2 for consumption guidelines). The double line is a break in the x-axis to better depict and view lower Hg concentrations

Because of uncertainty of Hg in African fish, Hg biomonitoring deliberations should consider tracking fish Hg concentrations in seven of the 16 (44%) fish families that average or range above the 0.22 µg/g, ww threshold of (see Table 2 for human meal frequency and Fig. 7 for the Hg profile) and account for differences in species, size class, type of freshwater system (e.g., lake vs. river), association with small-scale gold mining activities and subsistence communities, as well as seasonality (wet versus dry season; Kouame et al. 2020).

Studies documenting Hg concentrations in fish from lakes contaminated through atmospheric deposition (vs. releases of Hg into the water from ASGM activities) reveal relatively low concentrations for fish communities as illustrated in the Okavengo Delta in Botswana (Black et al. 2011), Lake Tanganyika in Tanzania (Campbell et al. 2008), Aiba Reservoir in Nigeria (Atobatele and Olutona 2015), and in rift valley lakes (Campbell et al. 2003a) including Lake Tana (Habiba et al. 2017), Lake Victoria (Campbell et al. 2003b; Drouillard et al. 2024), and Lake Malawi (Kidd et al. 2003); although only 4% of inland water bodies have been sampled for Hg concentrations in fish (Hanna et al. 2015). Importantly, Hg concentrations in Nile perch (*Lates niloticus*) and tilapia (representing multiple genera), the two most important commercial species, tend to be <0.5 µg/g, ww (Hanna et al. 2016; Drouillard et al. 2024). Conversely, snakeheads (Channidae) and African pike (Hepsetidae) are generally elevated and may be important for Hg biomonitoring for human health purposes.

However, local studies within ecosystems that are sensitive to Hg input indicate aquatic ecosystems in Africa can have elevated Hg levels of concern in fish and other aquatic food items used by humans, especially when associated with ASGM activities. Concentrations in high trophic level fish from lakes and rivers in Burkina Faso, Egypt, Ghana, Kenya, Senegal, South Africa, Tanzania, Zimbabwe as well as estuaries in Cote d’Ivoire have documented Hg levels of concern for human consumption (Ouédraogo and Amyot 2013; Hanna et al. 2015; Niane et al. 2015; Rajaee et al. 2015; Gbogbo et al. 2017; Walters et al. 2017; Elawady et al. 2019; Mason et al. 2019, 2022; Debrah et al. 2020; Makaure et al. 2023; van Rooyen et al. 2023).

A review of fish Hg concentrations in the GBMS database (6,126 individuals in 41 families from 183 species) from 171 locations in 21 African countries in 66 papers found mean Hg concentrations were relatively low (i.e., below the 0.22 µg/g, ww human health threshold commonly used). Sixteen families with ≥15 individuals are depicted (Fig. 7). Hanna et al. (2015) reviewed 30 studies in Africa that documented fish Hg concentrations and found that only locations near ASGM operations had mean Hg levels above recommended human health guidelines. A similar pattern was found in Ghana (n = 1305 measures in 65 species) where only sampling sites associated with ASGM had fish Hg levels that exceeded human health thresholds, especially for those species at high trophic levels (Rajaee et al. 2015; Kortei et al. 2020).

Piscivore fish species that have been identified in the GBMS database to have muscle tissue over 0.22 µg/g, ww include the saddled bichir (*Polypterus endlicherii*), African pike (*Hepsetus spp*.), African tigerfish (*Hydrocynus vittatus*), snakeheads, and multiple catfish species within the order Siluriformes, including species within the families of Bagridae, Clariidae, Claroteidae, Mochokidae, and Schilbeidae.

#### Freshwater fish - South America

##### Rationale and caveats for biomonitoring

The major river basins of South America, including the Magdalena, Orinoco, Amazon, and La Plata, support a large freshwater fishery, providing livelihoods for small-scale artisanal fishers as well as major commercial enterprises (Barletta et al. 2010). In the remote interior areas of South America, indigenous communities are highly dependent on freshwater resources for subsistence, and for communities with high fish consumption (FAO 2018), the risk of MeHg exposure can be high (Uryu et al. 2001; Passos et al. 2008; Oliveira et al. 2010; Olivero-Verbel et al. 2015; Hacon et al. 2020; Montaña et al. 2021). Research over several decades in the Amazon Basin has repeatedly identified a link between a diet high in fish, especially piscivorous and omnivorous species, and elevated Hg concentrations in human biomarkers such as hair (Bidone et al. 1997; Lebel et al. 1997; Castilhos et al. 1998; Boischio and Henshel 2000; Bastos et al. 2006; Faial et al. 2015; Ouboter et al. 2018; Feingold et al. 2020).

**Fig. 8:**
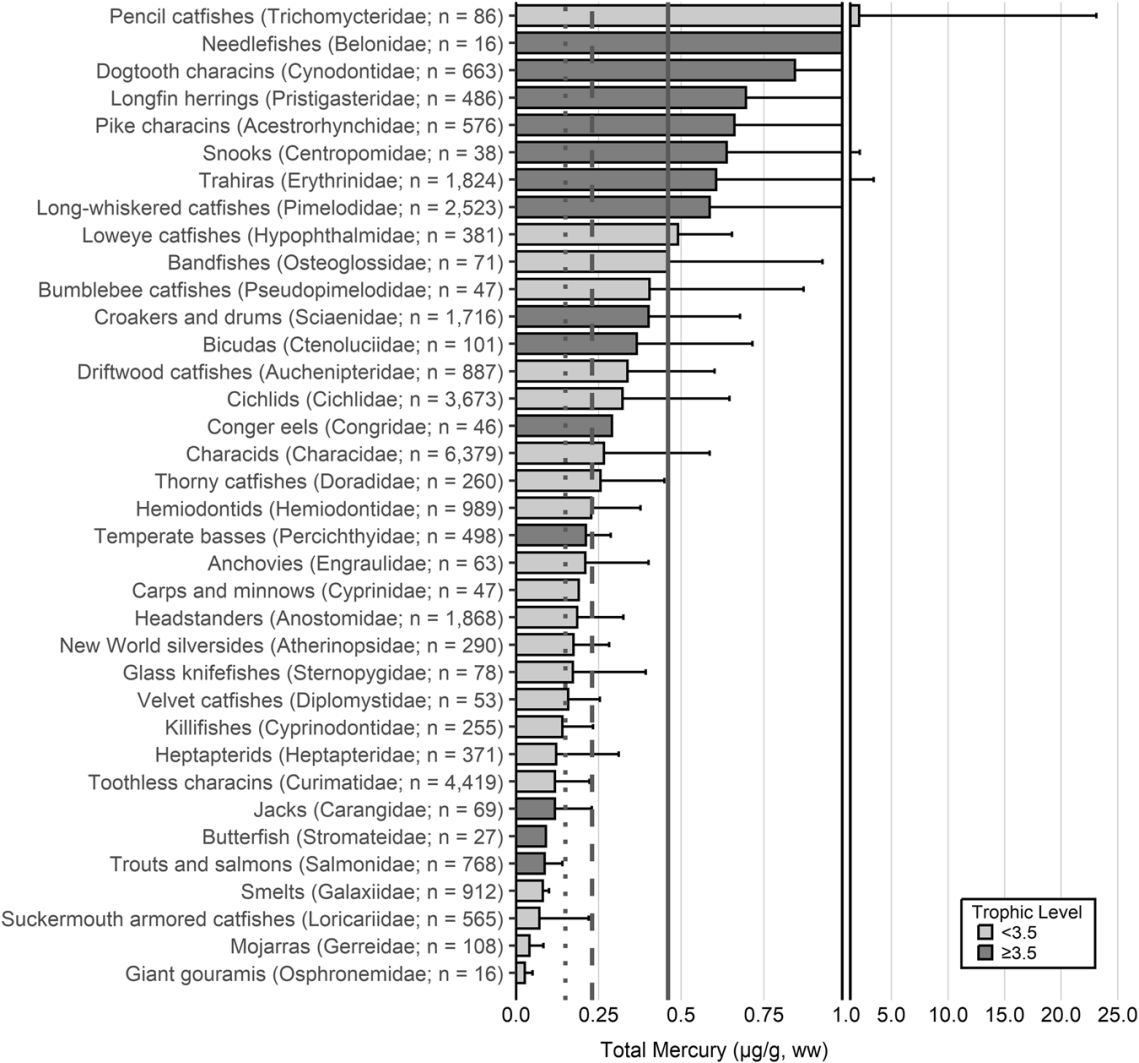
Mercury concentrations in freshwater fish in South America (including estuarine species). Gray bars illustrate the arithmetic mean ± SD of total Hg concentrations (µg/g, ww) in muscle tissue of 36 teleost fish families selected from the GBMS database that represents South America. USEPA-USFDA human health thresholds for Hg consumption (µg/g, ww) are shown as dotted (0.15) dashed (0.23) and solid (0.46) lines (see Table 2 for consumption guidelines). The double line is a break in the x-axis to better depict and view lower Hg concentrations

Mercury biomonitoring deliberations should consider tracking fish Hg concentrations in 17 of the 36 (47%) fish families that average or range above the 0.46 µg/g, ww threshold of ”choices to avoid” (see Table 2 for human meal frequency and Fig. 8 for the Hg profile) and accounting for differences in species, size class, type of freshwater system (e.g., lake vs. reservoir vs. river), and association with small-scale gold mining activities and Indigenous and subsistence communities. Seasonality of sample collection is also important as low water time periods result in higher fish Hg concentrations than otherwise (Nyholt et al. 2022).

Four hotspots in the Amazon Region have been identified as areas of particular concern because of the magnitude of ASGM activities since 2002 (Alvarez-Berríos and Mitchell Aide 2015): (1) the Madre de Dios region, Peru (Asner and Tupayachi 2017; Caballero Espejo et al. 2018; Diringer et al. 2020; Barocas et al. 2023); (2) the Guiana Shield region that includes French Guiana, Guyana, and Suriname; (3) the Tapajós–Xingú region (Malm et al. 1995; dos Santos et al. 2000; Nevado et al. 2010; Lino et al. 2019; Passos et al. 2008) that includes Central Amazon (Kasper et al. 2014) and the Madeira River (Bastos et al. 2006, 2015; Mussy et al. 2022; da Silva Montes et al. 2022) in Brazil; and (4) the Magdalena–Urabá region of Colombia (Ashe 2012; Hacon et al. 2014; Martinez et al. 2018; Gonzalez et al. 2019; Hacon et al. 2020). These and other Hg point sources (e.g., petroleum extraction; Webb et al. 2015) that are connected with river floodplain habitats, where daily and seasonal water level fluctuations can be extensive, appear to be sensitive to elevated methylation rates - during both droughts (Azevedo et al. 2018) and flood periods (da Silva et al. 2019).

The GBMS database for South America contains over 144 peer-reviewed publications on fish Hg concentrations from more than 319 different locations; 38,126 Hg concentrations from 350 species in 62 families are represented. The Hg dataset for fish in South America is taxonomically diverse with a description of Hg concentrations shown for 36 families with a sample size ≥15 (Fig. 8).

Of the 63 fish families with representative data, 49% have mean Hg concentrations over 0.22 µg/g, ww. Fifteen fish families exceed the USEPA human safety threshold (0.46 µg/g, ww on average) for avoiding consumption. The family Trichomycteridae (pencil or parasitic catfish) demonstrates the highest muscle Hg concentrations; this family is diverse with over 40 genera reflecting nearly 300 species. The most sampled taxa include high trophic-level species within the genus *Hoplias* (tigerfishes), *Serrasalmus* (piranhas), *Pseudoplatystoma* (sorubim catfishes), *Cichla* (neotropical cichlids), *Salminus* (dorado), and *Hoplias* (wolf fish). The GBMS dataset highlights areas of broad freshwater fish sampling on the continent, specifically in Brazil (Malm 1998; Ferreira da et al. 2019), and with some additional coverage in Colombia (Olivero et al. 1998; Salazar-Camacho et al. 2020), Ecuador (Webb et al. 2004), French Guiana (Richard et al. 2000; Gentès et al. 2019), Peru (Gammons et al. 2006; Diringer et al. 2015; Martinez et al. 2018), and Suriname (Ouboter et al. 2012; Vreedzaam et al. 2023); as well as in estuaries in Argentina (Marcovecchio et al. 2001) and Suriname (Mol et al. 2001).

From these data, it is also possible to identify ecologically sensitive hotspots of concern for ecological and human health (see Fig. 1). Much of the research on Hg to document ecological and human Hg exposure has been conducted in downstream areas potentially impacted by ASGM activities (Olivero-Verbel et al. 2015; Diringer et al. 2015; Moreno-Brush et al. 2016; Salazar-Camacho et al. 2017; Ouboter et al. 2018; Watson et al. 2020), especially when contaminated rivers flow into lakes (Lake Titicaca in Peru; Gammons et al. 2006) or reservoirs (Brokopondo Reservoir, Suriname; Ouboter et al. 2012; Tucurui Reservoir, Arrifano et al. 2018; and other reservoirs; Pestana et al. 2019). Watersheds that are downwind from ASGM activities also may have elevated biotic Hg concentrations (Gerson et al. 2022).

#### Freshwater fish – Asia

##### Rationale and caveats for biomonitoring

**Fig. 9:**
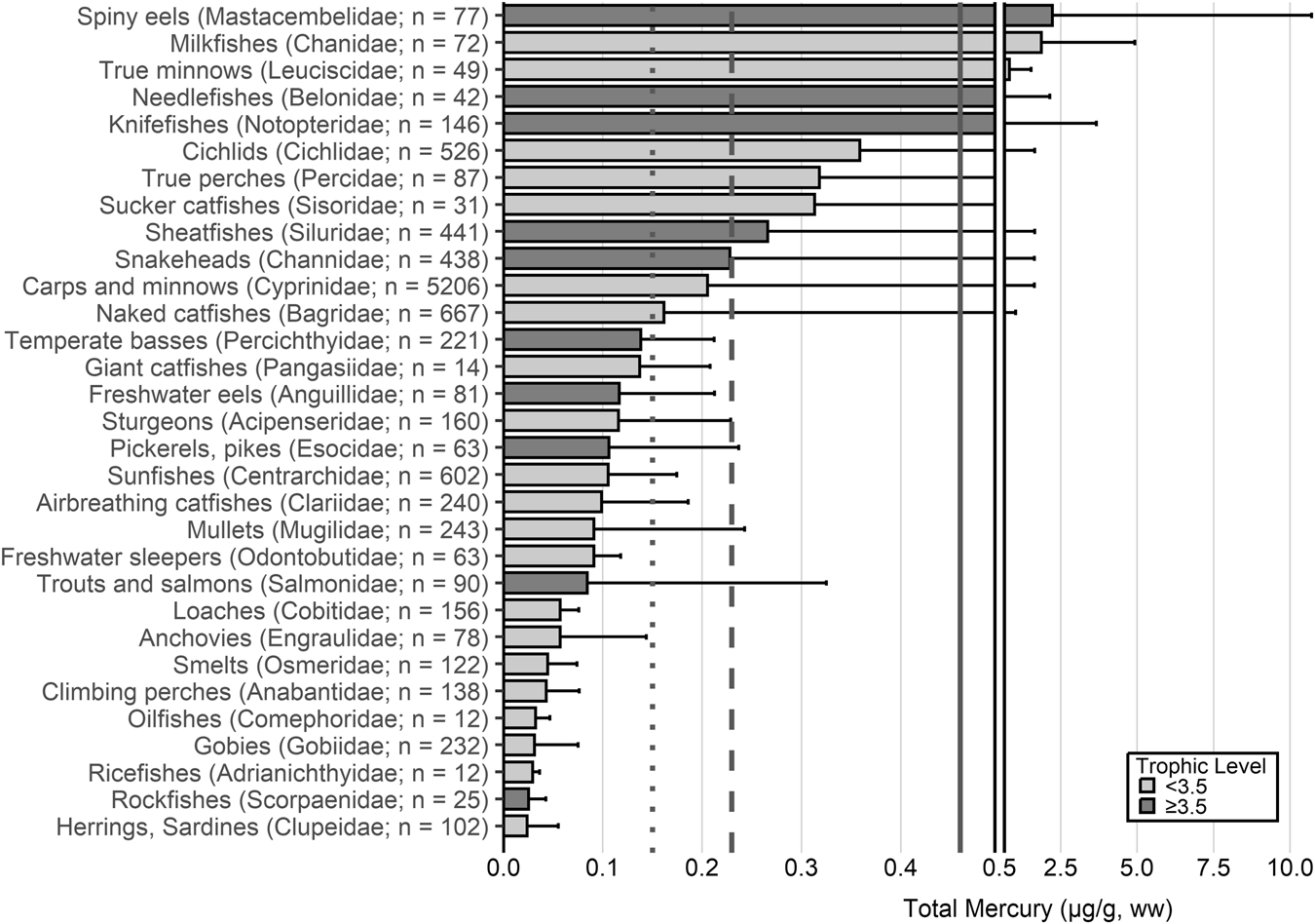
Mercury concentrations in freshwater fish in Asia (including estuarine species). Gray bars illustrate the arithmetic mean ± SD of global total Hg concentrations (µg/g, ww) in muscle tissue of 31 teleost fish families selected from the GBMS database that represents Asia. USEPA-USFDA human health thresholds for Hg consumption (µg/g, ww) are shown as dotted (0.15) dashed (0.23) and solid (0.46) lines (see Table 2 for consumption guidelines). The double line is a break in the x-axis to better depict and view lower Hg concentrations

Mercury emissions in Asia represent some of the highest in the world, and reductions are now being attempted by countries, such as China and India, in part due to the provisions of the Minamata Convention (Sharma et al. 2019; Feng et al. 2022). While freshwater ecosystems in the vast Asian landscape are dominated by rivers in the south (e.g., Ganges, Indus, Mekong, Yangtze, and Yellow rivers) and the north (Lena and Ob Rivers) and numerous oligotrophic lakes in the north – relatively few studies have documented biotic Hg concentrations in this most water-stressed continent of the world. Mercury biomonitoring deliberations should consider tracking fish Hg concentrations in 12 of the 31 (39%) fish families that average or range above the 0.46 µg/g, ww threshold of “choices to avoid” (see Table 2 for human meal frequency and Fig. 9 for the Hg profile) and account for differences in species, size class, type of freshwater system (e.g., lake vs. reservoir vs. river), overfishing, and association with ASGM areas and rice fields.

The knowledge of Hg in freshwater fish for Asia is sparse for the size and diversity of the landscape. China is the largest nation for the consumption and export of fish and fish products; however reported Hg concentrations in fish are generally low (Wang and Wang 2019; Feng et al. 2022; Souza-Araujo et al. 2022), even in relatively new reservoirs, such as in the Guizhou Province (Yan et al. 2010a, 2010b) and in the Three Gorges Reservoir (Xu et al. 2018; Wang et al. 2019b), and when there are point sources such as abandoned Hg mines (Qiu et al. 2009) and others (Zhu et al. 2012). A comprehensive review over the past 10 years did document significant geographical differences from the north to the south: Fish in north China rivers had more Hg than those in south China (Zhang and Wong 2007); Additionally, the Tibetan Plateau exhibited the highest total Hg levels (up to 0.87 µg/g, ww) (Wang and Wang 2019). Fish Hg concentrations are generally low across China because of fast-growing farmed and stocked fish species dominance, coupling with declining wild fish populations in freshwaters where trophic level enrichment of MeHg is generally dampened by eutrophication, and water chemistry parameters (e.g., alkaline pH and low dissolved organic carbon) that are not conducive to high methylation rates (Cheng and Hu 2012; Liu et al. 2012). Minimal shoreline wetland area due to anthropogenic activities, lower trophic biomagnification factor in temperate freshwater food webs compared to boreal ones, and the general lack of trophic level 4 fish in eutrophic waters also contribute to patterns of lower fish Hg concentrations (Chen et al. 2008; Liu et al. 2012; Xu et al. 2018; Wang and Wang 2019; Wu et al. 2019; Jing et al. 2020).

Exceptions have been reported by Razavi et al. (2014) in the mesotrophic Qiandao Lake, where the food web in this remote 50-year-old reservoir of East China demonstrated a high degree of omnivory and a long food web with trophic level up to 4.9 including wild fish species. Consequently, wild fish in this reservoir had generally higher Hg concentrations (up to 1.78 µg/g, ww in the Mandarin fish (*Siniperca chuatsi*) than stocked fish (up to 0.58 µg/g, ww in herbivorous fish species, such as the goldfish [*Carassius auratus]*). Although average fish Hg concentrations from Qiandao Lake was well under 0.5 µg/g, ww, it was still significantly higher than fish from Taihu Lake and reservoirs in Guizhou. Nevertheless, a further meta-analysis of both freshwater and marine consumer fish in China over the last three decades from 1980 presented that fish Hg concentrations were decreasing despite increased Hg emission over time, a phenomenon likely due to overfishing and aquaculture (Zhang et al. 2022).

The GBMS database includes 14,093 fish Hg concentrations from 75 families representing 304 species based on 93 publications. Of those families, 31 are featured here that have sufficient sample sizes (≥12) or are otherwise of interest (e.g., high Hg concentrations) (Fig. 9). The highest Hg concentrations were in spiny eels (Mastacembelidae) with mean Hg concentrations over 2.0 µg/g, ww. Milkfish, needlefish, true minnows, and knifefish families had particularly elevated Hg concentrations, averaging over 0.5 µg/g, ww. Several families have wide variation in Hg concentration that indicate other factors such as species, size class, freshwater type, and location are important to understand prior to identifying best bioindicators.

Representation of Asian freshwater fish Hg concentrations outside of China are relatively sparse. In Russia, a summary of 21 fish species from the Oka, Moskva, Osetr, Volga, and Akhtuba Rivers found relatively low Hg concentrations with average concentrations at or under 0.26 µg/g, ww (Gorbunov et al. 2016). However, lakes sampled in Russia contained fish with relatively elevated Hg concentrations (Buck et al. 2019; Dudarev et al. 2019).

Investigation of selected Korean reservoirs from 2016 to 2020 showed low fish Hg concentrations well under 0.5 µg/g, ww in barbel steed (*Hemibarbus labeo)*, largemouth bass, and bluegill (Jung et al. 2022). Whereas in Japan, Watanabe et al. (2021) presented 95% of salmon and trout samples contained MeHg at less than 0.05 µg/g, ww, extracted from the Japanese National Health and Nutrition Survey. Otherwise, there is no recent publicized freshwater fish Hg data in Japan since Matsunago (1975), who reported average fish Hg concentrations of 0.72 µg/g, ww in three different fish species (dace, crucian carp, and *Zacco* spp.) sampled in two rivers that received Hg mining waste at the time.

Freshwater fish Hg concentrations in certain South East Asia countries remain safe despite rapid urban development activities: Low fish Hg concentrations (0.051 ± 0.04 µg/g, ww) in Mekong River confirmed its pristine state of the ecosystem of Vientiane area, Laos (Guédron et al. 2014); Lobus and Komov (2016) validated that 76% of freshwater fish contained less than 0.10 µg/g, ww of total Hg in muscle tissue sampled from rivers, lakes, and reservoirs of Central and South Vietnam. Freshwater fish in Cambodia are generally low and do not exceed 0.12 µg/g, ww in striped snake-head fish (*Channa striata****)***, common climbing perch (*Anabas testudineus)* and peacock eel (*Macrognathus siamensis)*, as demonstrated by Agusa et al. (2005a, 2005b). Fish Hg remain well below 0.46 µg/g, ww as seen in indicator species collected from West Bay area of Laguna Lake, in the Philippines (Cuvin-Aralar 1990).

High levels of Hg in fish stocks have been found mainly in coastal areas in Thailand, Indonesia, and India. With increasing inland industrial activities in these regions, increased freshwater fish Hg concentrations have been reported in industrial sites in Thailand: Sampled striped snakehead from Tha Tum industrial complex contained elevated fish Hg concentrations up to 0.52 µg/g, ww (Tremlová et al. 2017). Surprisingly, fish Hg was even higher up to 0.56 µg/g, ww in the same species sampled from Thap Lan National Park neighboring Prachinburi industrial park. In another scenario of adverse anthropogenic impact on fish Hg and human health, Castilhos et al. (2006) reported elevated fish Hg concentrations of 0.58 ± 0.44 µg/g, ww with more than 45% of fish having Hg levels above 0.46 µg/g, ww across 154 specimens of 10 freshwater species from gold mining areas in Tatelu, Indonesia. Fish from the Ganges River at West Bengal in India was investigated showing that wallago catfish *(Wallago attu)* possessed high Hg content at 0.93 ± 0.61 µg/g, ww, while small-sized fishes from the same sampling site showed low fish Hg concentrations below DL (Pal et al. 2011).

#### Freshwater, estuarine, and marine fish – Australia

##### Rationale and caveats for biomonitoring

**Fig. 10:**
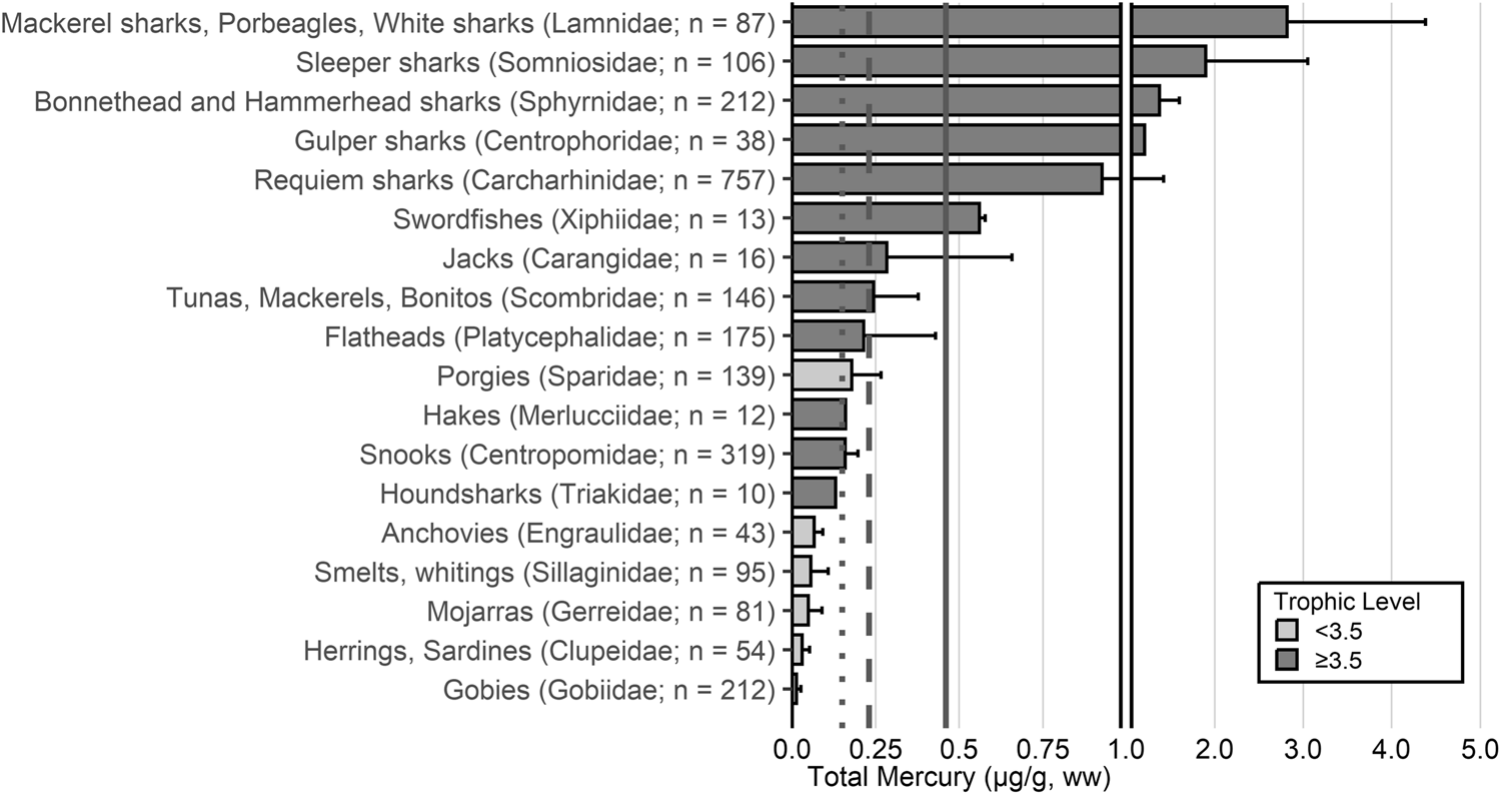
Mercury concentrations in freshwater, estuarine and coastal fish sampled from the continental shelf in Australia. Gray bars illustrate the arithmetic mean ± SD of global total Hg concentrations (µg/g, ww) in muscle tissue of 18 teleost and elasmobranchs families of interest selected from the GBMS database that represents Australia. Centropomidae (Snooks) includes barramundi. USEPA-USFDA human health thresholds for Hg consumption (µg/g, ww) are shown as dotted (0.15) dashed (0.23) and solid (0.46) lines (see Table 2 for consumption guidelines). The double line is a break in the x-axis to better depict and view lower Hg concentrations

Australia, including Tasmania and numerous islands, has rich and varied ecosystems, from desert to tropical rainforest and straddles the Indian and Pacific Oceans. Australia’s coastal seas include the world’s largest barrier reef, the Great Barrier Reef, encompassing almost 350,000 km^2^ off the northeast coast, and is a biodiversity hotspot. Mercury biomonitoring deliberations should consider tracking fish Hg concentrations in 7 of 18 (38%) fish families that average or range above the 0.46 µg/g, ww threshold of “choices to avoid” (see Table 2 for human meal frequency and Fig. 10 for the Hg profile) and account for differences in species distributions and abundance, as well as location across the continent.

The GBMS dataset for fish (elasmobranchs and teleosts) from Australia extending to the continental shelf (including estuarine and freshwater samples) includes 2646 Hg concentrations for 100 species in 48 families – including 18 families of greatest interest – from 16 publications (Fig. 10). The findings indicate that the five families with the highest Hg concentration are elasmobranchs, including mackerel sharks/Porbeagles/white sharks (Lamnidae; 2.82 ± 1.56 µg/g; n = 87), sleeper sharks (Somniosidae; x = 1.90 ± 1.15 µg/g; n = 106), Bonnethead and Hammerhead sharks (Sphyrnidae; 1.37 ± 0.23 µg/g; n = 212). The highest teleost families include swordfishes (Xiphiidae; 0.56 ± 0.02; n = 13), jacks (Carangidae; 0.28 ± 0.38; n = 16) and tunas, mackerels, bonitos (Scombridae; 0.24 ± 0.13; n = 146). Species that have the lowest risk of Hg contamination to people include gobies, herrings/sardines, and mojarras (Fig. 10).

Several independent research studies have been conducted to evaluate Hg concentrations in fish collected from Australian estuaries and nearshore coastal ecosystems with the focus on understanding health implications, environmental processes, and anthropogenic impacts (Gagnon et al. 2016; Maher et al. 2020; Butler et al. 2022). Mercury contamination of fish in nearshore Australian marine environments is not evident except at several locations with historical Hg contamination (Maher et al. 2020). In Australia, as in other regions like the Caribbean Sea, areas that of particular concern for elevated Hg concentrations in fish include wetland (particularly mangroves) and estuarine habitats. Butler et al. (2022) found that barramundi (*Lates calcarifer*) in floodplain wetlands concentrated Hg at almost twice the level of those that remained in saline habitats.

The Australian Government’s scientific research organization, the Commonwealth Scientific and Industrial Research Organization (CSIRO), has recently conducted an extensive review of published data on contaminants (including Hg) in sea turtles, birds, and marine mammals. Most samples were collected opportunistically in Australia between the 1970s and 2022 (Jarolimek et al. 2023). While long-term datasets are not available for the same species, the review provides baseline information on Hg levels in Australian marine fauna. Lastly, the Food Standards Australia and New Zealand (FSANZ) conducts the Australian Total Diet Study (ATDS) and evaluates Hg levels in a wide range of Australian foods (including seafood) with the aim to estimate the dietary exposure of the Australian population to Hg, identify risks and risk management options, and provides recommendations on the safe consumption of fish (note that Fig. 10 uses the standards set by the USEPA and USFDA (Table 2).

#### Freshwater fish – North America and Europe

##### Rationale and caveats for biomonitoring

**Fig. 11:**
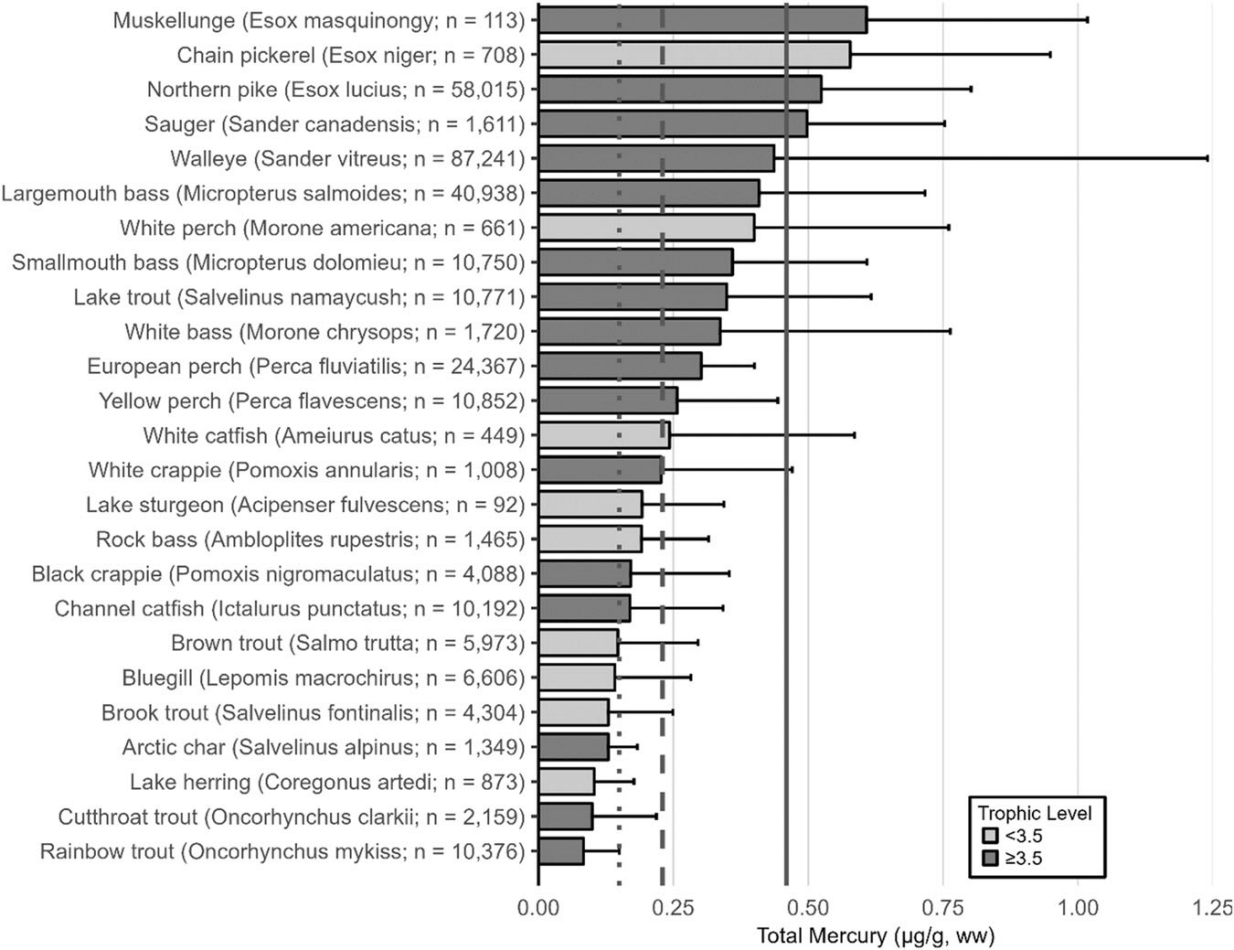
Representative Hg concentrations in selected game fish species from North America and Europe. Gray bars illustrate the arithmetic mean ± SD of global total Hg concentrations (µg/g, ww) in muscle tissue of 25 teleost game fish species selected from the GBMS database that represent North America and Europe. USEPA-USFDA human health thresholds for Hg consumption (µg/g, ww) are shown as dotted (0.15) dashed (0.23) and solid (0.46) lines (see Table 2 for consumption guidelines)

The freshwaters of North America and Europe are extensive, and the lakes and rivers have a diverse fish community from temperate to Arctic waters. While the Great Lakes Basin in the U.S. and Canada provide a dominant recreational fishery for the region, riverine and lake fisheries across this extensive area are important contributors to local economies (Evers et al. 2011a; Wiener et al. 2012b) and for some areas, subsistence purposes such as in Arctic Inuit communities (AMAP 2021). Mercury biomonitoring deliberations should consider tracking fish Hg concentrations in 12 of the 25 (48%) fish families that average or range above the 0.46 µg/g, ww threshold of ”choices to avoid” (see Table 2 for human meal frequency and Fig. 11 for the Hg profile) and account for differences in species, size class, type of freshwater system (e.g., lake vs. reservoir vs. river), and association with Indigenous and subsistence communities.

Both continents have or have had well established Hg monitoring programs that generally revolve around game fish, though there has been an absence of harmonized, national monitoring programs that span decades of time. However, monitoring efforts have generated hundreds of thousands of fish analyzed for Hg over the past 4–5 decades. In the U.S., such programs have focused on the Great Lakes, interior lakes, rivers, and streams including in the Great Lakes Fish Monitoring and Surveillance Program (Monson et al. 2011; Zhou et al. 2017) and the National Rivers and Streams Assessment (Wathen et al. 2015a, 2015b). Mercury data have also been summarized for lakes and rivers for the Northeast (Kamman et al. 2005; Millard et al. 2020), Midwest (Monson et al. 2011), and the West (Eagles-Smith et al. 2016a). In Canada, massive datasets (>300,000 Hg concentrations in the Canadian Fish Mercury database; Depew et al. 2013) describing spatial and temporal game fish Hg trends have been summarized as well (Gandhi et al. 2014; Eagles-Smith et al. 2016a). Similar efforts in Europe, especially in Scandinavia – spanning 55°–70°N, also provide definitive baseline information that can be used for temporal comparison purposes (Braaten et al. 2019).

To evaluate spatial and temporal variation of Hg in fish, the game fish Hg data are generally normalized with regard to size using well-established standard units (e.g., 55 cm and 1 kg for northern pike; Sorensen et al. 1990). Such standards have been applied to robust Hg datasets (i.e., >50,000 data points) in the U.S., Canada, and northern Europe for pike, bass, and walleye (Johnels et al. 1967; Kamman et al. 2005; Monson 2009; Monson et al. 2011; Gandhi et al. 2015; Eagles-Smith et al. 2016a) for human health assessments and using perch (*Perca* spp.) (Scheuhammer et al. 2016) for evaluating concerns for ecological health purposes. Other normalizing techniques include using individual fish weight and Hg concentration in combination with fish species information and sampling year to find expected Hg concentration for fish at a standard weight. In Braaten et al. (2019), multiple linear regression models were applied to describe Hg concentrations, where potential explanatory variables included fish weight, fish species, sampling year, and the interaction terms year × species and weight × species, to evaluate changes in fish Hg concentrations with weight and species over time.

Records of Hg in freshwater fish across Fennoscandia (Norway, Sweden, Finland, Kola Peninsula in Russian Federation) have been collected for over 50 years (since 1965) in almost 3000 lakes and rivers and collated into a single database by the International Cooperative Programme for assessment and monitoring of the effects of air pollution on rivers and lakes (or ICP Waters), under the UNECE Air Convention (Braaten et al. 2017) – only the peer-reviewed published data are in the GBMS database. Fish Hg concentrations vary widely among lakes in Fennoscandia owing partly to differences in local and regional Hg pollution in the lakes, but particularly factors controlling net methylation, trophic structures, and subsequent biomagnification (Braaten et al. 2019).

Measured Hg concentrations in the south (55°N–60°N) of Fennoscandia are generally higher than in the north (60°N–70°N), with over 40% of all lakes containing fish muscle Hg concentrations exceeding the WHO/FAO limit of 0.5 µg/g, ww widely used as a trigger for human consumption safety in Europe. The dataset includes important species for recreational fishing such as northern pike (*Esox lucius*) (South: 0.63 ± 0.01 µg/g,ww [n = 24,849], North: 0.60 ± 0.01 µg/g,ww [n = 3360]), Arctic char (*Salvelinus alpinus*) (South: 0.45 ± 0.18 µg/g,ww [n = 284], North: 0.11 ± 0.10 µg/g,ww [n = 514]), European perch (*Perca fluviatilis*) (South: 0.26 ± 0.02 µg/g,ww [n = 20,276], North: 0.20 ± 0.01 µg/g,ww [n = 2326]), and brown trout (*Salmo trutta*) (South: 0.14 ± 0.03 µg/g,ww [n = 1816], North: 0.16 ± 0.40 µg/g,ww [n = 230]). Half a century of fish Hg concentrations in Fennoscandian lakes have shown a clear decline (Åkerblom et al. 2014; Braaten et al. 2017, 2019). However, there is no consistent decline in lakes for which Hg originates from atmospheric sources only (e.g., Rask et al. 2024). Closing of local industrial pollution sources over the past 50 years is likely to have led to a reduction in fish Hg concentrations.

The GBMS database for North America and Europe contains over 170 peer-reviewed publications on fish Hg concentrations from more than 240 sites across 100 different waterbodies (e.g., lakes, rivers, estuaries, and bays); more than 253,667 individual fish from more than 240 genera are represented. The Hg dataset for fish is robust, especially for game fish, of which 25 species of 8 families are featured here as key bioindicators for human health purposes, including walleye (*Sander vitreus*), largemouth bass (*Micropterus salmoides*), and northern pike (Fig. 11). Of these species, 48% have a mean Hg concentration over the USEPA human health benchmark of 0.46 µg/g, ww.

#### Seabirds – human consumption assessment

##### Rationale and caveats of biomonitoring

Although hunting of seabirds is not as common globally as it once was, in some areas of the world, marine birds (waterfowl, shorebirds, and seabirds) and their eggs can still be a regular and necessary food source for remote subsistence communities. This is especially true across the circumpolar Arctic and Subarctic (e.g., Naves 2018; Otsuki et al. 2024), and in a scattering of small island nations, like Grenada (Smart et al. 2020), where alternative sources of protein may be limited at times, driving both legal and illegal consumptive harvests of seabirds.

In North America and Europe, there are several examples of both indigenous and non-indigenous harvests of seabirds and/or their eggs, some with significant cultural relevance. For example, Baffin Island in the eastern Canadian Arctic is an ecologically significant area, supporting many species of seabirds and marine mammals. Inuit communities across the Canadian Arctic, such as those on Baffin Island, rely heavily on a variety of marine resources, including seabirds (Chan et al. 1995; Mallory et al. 2004). The presence of elevated Hg in seabirds throughout the Canadian Arctic is well established (Muir et al. 1999; Mallory et al. 2004; Campbell et al. 2005; Mallory and Braune 2012; Burnham et al. 2021), with either stable or increasing Hg trends observed in seabirds in recent decades (Braune et al. 2015, 2016).

Indigenous People and subsistence hunters in Alaska also have a long history of harvesting and consuming marine resources, including seabirds and their eggs (Burger et al. 2007; Naves 2009). Mercury accumulation in seabirds from the Aleutian Islands has also been well documented (Burger et al. 2007, 2008, 2009; Burger and Gochfeld 2009; Savoy et al. 2017). According to Burger et al. (2007), 90% of households from an Aleutian village consumed birds to some degree each year. Previous studies have shown that some seabird species from the Aleutian Islands contain edible parts (e.g., breast meat, eggs) with Hg levels that approach or exceed human consumption advisory levels (0.22 µg/g, ww; Burger et al. 2007, 2009). Although estimates of subsistence harvests of seabirds may have declined somewhat across the state in recent decades, birds and their eggs remain a necessary source of nutrition in some particularly isolated Alaskan communities (e.g., the St. Lawrence–Diomede Islands; Naves 2018), as well as remaining culturally important across the region. Abundance and ease of collection mean the eggs of the glaucous-winged gull (*Larus glaucescens*) are highly sought after, and the number collected each year can exceed 6000 (Naves 2009). Larger gull species regularly exhibit elevated Hg concentrations within a seabird colony. The eggs of murres and gulls represent the majority of egg harvests, but the eggs of many smaller seabird species, such as auklets and terns, are also widely harvested in Alaska (Naves 2018).

**Fig. 12:**
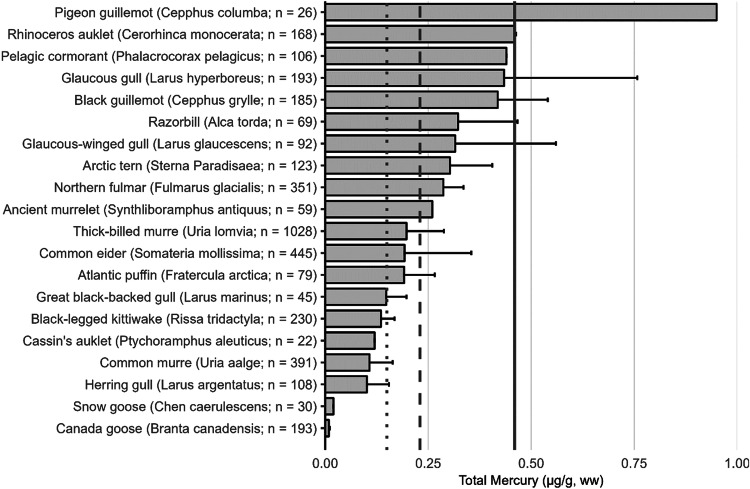
Mercury concentrations from Arctic and subarctic birds. Gray bars illustrate the arithmetic mean ± SD of global total Hg concentrations (µg/g, ww) in eggs of 20 species selected from the GBMS database. USEPA-USFDA human health thresholds for Hg consumption (µg/g, ww) are shown as dotted (0.15) dashed (0.23) and solid (0.46) lines (see Table 2 for consumption guidelines)

The GBMS database includes 3943 individual eggs from 20 species of marine bird of interest in the Arctic and subarctic were included from 32 publications. Generally, Hg concentrations in seabirds in the Canadian Arctic are below levels associated with health effects in wildlife. Whereas edible parts for human consumption (breast muscle, eggs) (Fig. 12) may approach or exceed the action level (0.22 µg/g, ww) (Table 2). For example, a recent review of Hg concentrations in Arctic seabirds found that 50% of individuals sampled (n > 5000) showed tissue Hg concentrations exceeding 0.20 µg/g, ww (Chastel et al. 2022).

Using kittiwakes (*Rissa* spp.), fulmars *(Fulmarus* spp.) and murres *(Urias* spp.) as bioindicators, Hg concentrations in seabird eggs from the Canadian Arctic have increased significantly over recent decades (Braune 2007; Braune et al. 2016; Burnham et al. 2021). In the Aleutian Islands of Alaska, there has been an effort to quantify MeHg uptake by local North Pacific fisheries and wildlife due to potential cumulative inputs of Hg from historic military activity (Burger and Gochfeld 2006; Anthony et al. 2007; Ricca et al. 2008), emissions from local volcanic activity (Ricca et al. 2008), and atmospheric and oceanic transport of Hg from Asia (Rocque and Winker 2004; Anthony et al. 2007; Driscoll et al. 2013) and Russia (Fisher et al. 2012).

Mercury biomonitoring deliberations could consider tracking bird egg Hg concentrations in the six seabird species that average or range above the 0.46 µg/g, ww threshold of restricted consumption (see Table 2 for human meal frequency and Fig. 12 for the Hg profile) and incorporate differences in species, interpretation of tissue type that incorporates species’ ecology, and association with Indigenous and subsistence communities.

#### Marine mammals – toothed whales

##### Rationale and caveats for biomonitoring

**Fig. 13:**
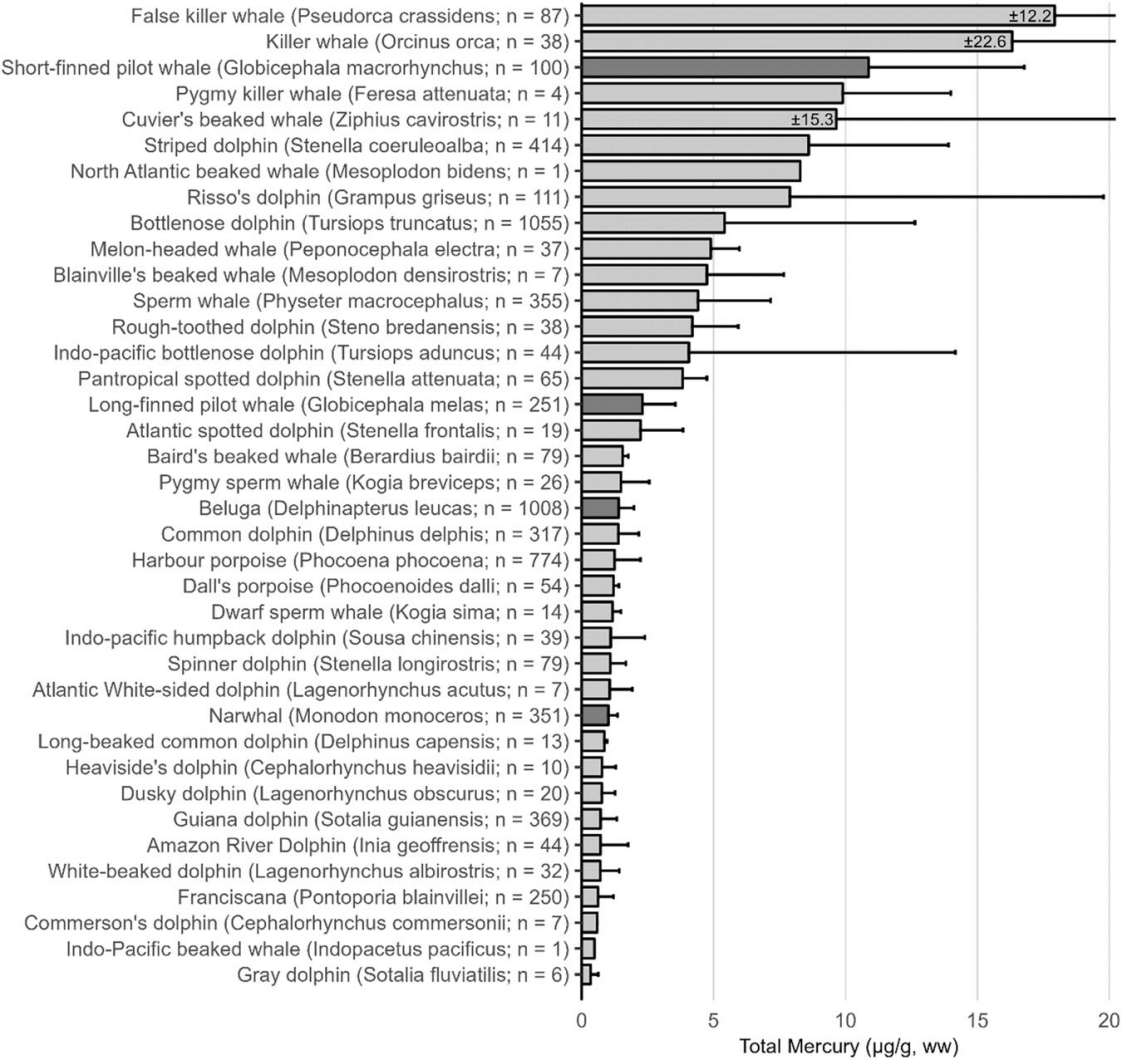
Muscle Hg concentrations in toothed whales. Bars illustrate the arithmetic mean ± SD of global total Hg concentrations (µg/g, ww) in muscle tissue of 38 toothed whale species. Dark gray bars are for species most regularly consumed by humans. All species exceed the USEPA-USFDA human health threshold for Hg consumption of 0.46 µg/g, ww level of “choices to avoid” (see Table 2 for consumption guidelines)

Toothed whales are the marine mammal taxa of greatest concern for human and ecological health because their Hg concentrations regularly exceed levels of concern for many species (Fig. 13). Although the effect levels in marine mammals are not well defined (Desforges et al. 2016), brain tissue levels are associated with neurotoxic effects (Dietz et al. 2013; Krey et al. 2015) and a study on bottlenose dolphins found lesions in the liver at 61 µg/g, ww and is being used by scientists as a benchmark for assessing ecological concern in marine mammals (Dietz et al. 2013). However, more recently groupings of marine mammals including toothed whales has been divided into groups such as “No risk, Low risk, Moderate risk, High risk and Severe risk” based on controlled experiments of harp seals (*Pagophilus groenlandicus*) (Dietz et al. 2022). Because liver tissue has limitations for assessing risk (e.g., levels of MeHg vary and are generally a small percentage of total Hg) and is not often consumed as a major part of the human diet, a more useful tissue to use for assessing the potential exposure of MeHg to humans is muscle tissue (AMAP 2021). Mercury biomonitoring deliberations should consider tracking muscle Hg concentrations in four of the toothed whale species that are regularly consumed and average above the 0.46 µg/g, ww threshold of ”choices to avoid” (see Table 2 for human meal frequency and Fig. 13 for the Hg profile and target species) and incorporate differences in species, home range, interpretation of tissue type that incorporates species’ ecology, and association with Indigenous and subsistence communities.

Many Indigenous Peoples and subsistence communities, mostly in the Arctic, depend on the harvest of marine mammals such as beluga (*Delphinapterus leucas)*, narwhal (*Monodon monoceros)*, and pilot whale (*Globicephala* spp.). Elevated tissue concentrations of Hg in these species are of high human health concern. Pilot whale harvesting by some subsistence-oriented countries, such as the Faroe Islands (Dam and Bloch 2000; Weihe and Debes Joensen 2012) have now ended (Krümmel and Gilman 2016; AMAP 2021), while in other countries whaling remains a concern such as in St. Vincent and the Grenadines (Fielding and Evans 2014; McCormack et al. 2020).Various species of porpoises and dolphins (Aubail et al. 2013; Correa et al. 2013), as well as beaked whales (which specialize in foraging on deep water cephalopods) also generally have elevated Hg tissue concentrations (Fig. 13; Bustamante et al. 2003; Garrigue et al. 2016, 2024). Other marine mammals, such as elephant seals (*Mirounga angustirostris*) foraging in the mesopelagic zone, also have elevated Hg concentrations (Peterson et al. 2015) and are especially vulnerable when concentrations can increase during haul-out periods when body mass declines as they fast and breed on land (Peterson et al. 2018).

The GBMS database includes 29,816 individuals of 79 species representing 199 publications. Based on the GBMS database, over 25 species of toothed whales have average muscle tissue Hg concentrations above 1.0 µg/g, ww. Therefore, toothed whales appear to be one of the more Hg contaminated groups of marine mammals. Toothed whales have global mean Hg concentrations in muscle tissue (3.2 µg/g, ww) that are well above general recognized consumption advisory levels recognized by most national standards (most relevant for beluga and pilot whales) because of the dependence of certain Arctic human communities on them) (Fig. 13).

Canadian Inuit regions published health advice outlining the importance of traditional country foods for Inuit health and well-being (AMAP 2015). However, Hg exposure through parts of the traditional diet has been found to be of concern in two Inuit regions. For example, health officials in Nunavik found that the main source of Hg exposure in their region is beluga meat and recommend that pregnant women and those of childbearing age should decrease their consumption of beluga meat. In Nunavut, Inuit women who are or may become pregnant are advised to avoid ringed seal (*Pusa hispida*) liver, while ringed seal meat is recommended as a healthy alternative.

Other marine mammals are also important bioindicators for human or ecological health and should be monitored. Ringed seals are a good candidate as they are common, widely distributed, and regularly harvested (Braune et al. 2015; see marine mammal section under “Ecological Health Bioindicators” for further information about ringed seals and other pinnipeds).

### Ecological health bioindicators

There are many species of fish and wildlife that are impacted by the adverse effects of elevated Hg on their physiology, behavior, and reproductive success (see summaries: Crump and Trudeau 2009; Dietz et al. 2013; Scheuhammer et al. 2015; Ackerman et al. 2016; Evers 2018; Whitney and Cristol 2017; AMAP 2021). Some are considered high profile species and are included by the IUCN on their Red List of Threatened Species, or formally listed by the United States Endangered Species Act of 1972.

The selection of the organism or suite of bioindicators depends on the objective. Taxa suitability may vary according to ecosystem interests (e.g., at habitat or biome levels of relevance), spatial gradient resolution (e.g., local, regional or global), temporal trends (e.g., short- or long-term), human or ecological health interests, endpoints of importance (e.g., reproductive impairment), known adverse toxicity thresholds (e.g., by tissue and taxa using endpoints of interest), sample availability (e.g., simple or challenging), and sampling outcome (e.g., non-lethal or lethal). A provisional list of some potential bioindicators for evaluating and monitoring environmental Hg loads for ecological health purposes can be grouped into four target biomes and their associated waterbodies and by major taxa of interest (Table 2; Evers et al. 2016). Some of these major taxa of interest for use as bioindicators are summarized below for sea turtles, birds, and marine mammals (the use of fish as indicators for Hg biomonitoring is covered under the “Human Health Bioindicators” section).

#### Sea turtles

##### Rationale and caveats for biomonitoring

Sea turtles have a wide distribution in tropical and subtropical regions, their dietary habits vary according to the species (e.g., herbivores, omnivores, and carnivores), and their lifespan is compatible with the residence time of Hg in the surface layer of the oceans (approximately 30 years) (Aguirre and Lutz 2004; Barbieri 2009; UNEP 2013; Evers 2018). These characteristics enable a spectrum of Hg concentrations through different trophic levels and facilitate the comparison among regions (Anan et al. 2001; Rodriguez et al. 2022). For instance, when examining the Hg levels in liver samples from juvenile green turtles (*Chelonia mydas*) from two distinct environments – one highly influenced by human activities (Bahia, Brazil) and another with less anthropogenic impact (Ceará, Brazil), the comparison revealed elevated concentrations of Hg in both the green sea turtles and their food items (algae and mollusks) from the highly affected location (Bezerra et al. 2015). Although comparisons were made with other tissues (e.g., muscle, kidney, and scutes), the liver was the only one that showed a significant relationship with the environmental concentrations, which can be explained by the role it plays in the storage and redistribution of recently ingested Hg (Schneider et al. 2013). Thus, the characteristics and function of each tissue are essential to understanding the metabolism of Hg and other metals in sea turtles.

Most Hg monitoring studies using sea turtles involved the utilization of internal organs (e.g., liver, kidney, and muscle), proving effective as estimators of environmental Hg concentrations (Bezerra et al. 2015; Rodriguez et al. 2022). However, due to their status as endangered species, this type of sampling is not viable for monitoring programs, while the use of non-invasive methods such as blood and keratinized matrices (e.g., scutes and nails) allows periodic monitoring (Sakai et al. 2000; Day et al. 2005; Bezerra et al. 2012; Rodríguez-Gutiérrez et al. 2020). Both tissue types can show more recent (e.g., blood) and longer term (e.g., scutes and nails) exposure (Day et al. 2005; Benjamin et al. 2018), characterizing bioaccumulation, patterns of temporal exposure to Hg and other trace metals (Bezerra et al. 2012; Schneider et al. 2015; Barraza et al. 2019; Villa et al. 2019). The study conducted by Day et al. (2005), found in the loggerhead sea turtle (*Caretta caretta*) along the southeastern coast of the United States a relationship between Hg concentrations in blood and scute samples and the foraging areas with greater contamination. Proximity to sources of contamination allows us to understand the differences between Hg concentrations in species of sea turtles, not only at the regional level but also at the global level.

According to Rodriguez et al. (2022), the high concentrations of Hg found for loggerhead sea turtles in the Mediterranean Sea compared to the Atlantic and Pacific Oceans, can be explained by the high density of submarine volcanoes and regional anthropogenic contamination (Selin 2009; Cinnirella et al. 2019; Tseng et al. 2021). Robust findings for Hg concentrations in fish and marine mammals supports the trend of elevated levels in sea turtles and the tendency to be higher than other areas within the Atlantic Ocean (Gworek et al. 2016; Kershaw and Hall 2019; Rodriguez et al. 2022).

Regions used as foraging areas by different species of sea turtles are especially important in Hg monitoring (Rodríguez-Gutiérrez et al. 2020) since diet is considered the main route of MeHg exposure (Gray 2002). Comprehending how the diet influences Hg concentrations in sea turtles allows for an understanding of the possible risks of consuming their meat and eggs (Green et al. 2010; Ross et al. 2016); generalized dietary groupings include vegetation (green sea turtles), jellyfish (leatherback sea turtles, *Dermochelys coriacea*), sponges (hawksbill sea turtles, *Eretmochelys imbricata*), and crustaceans (loggerhead sea turtle).

**Fig. 14:**
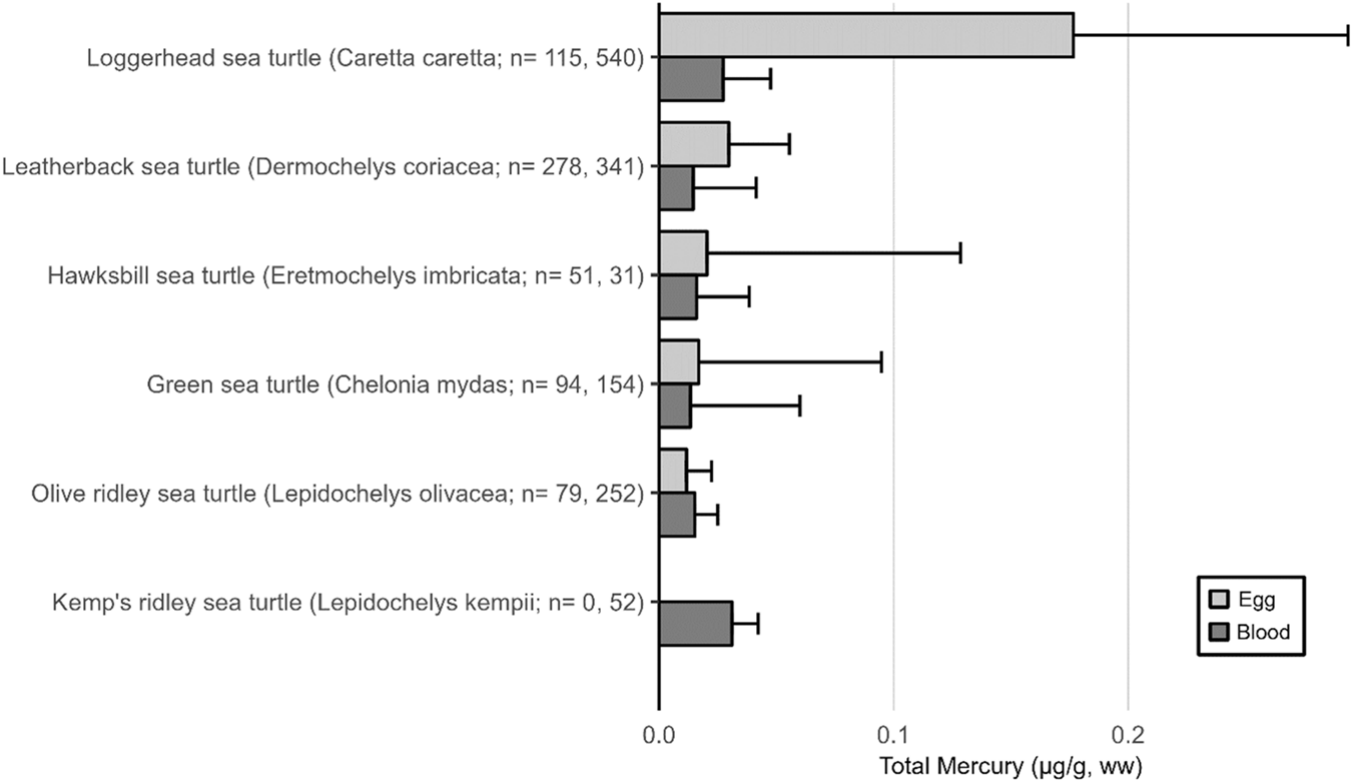
Mercury concentrations in sea turtles. Gray bars illustrate the arithmetic mean ± SD of global total Hg concentrations (µg/g, ww) in egg (light gray) and blood (dark gray) tissues of six sea turtle species

The GBMS database revealed that of the six species of sea turtles with tissue Hg concentrations (egg or blood), loggerhead sea turtles have the highest average egg Hg concentrations (Fig. 14). This finding holds significant implications for local communities that rely on this species’ eggs as supplemental protein (Ross et al. 2016; Guzmán et al. 2020; Tapilatu et al. 2020). Regions, where sea turtles may need to be monitored more intensively for elevated levels of Hg, include the Caribbean Sea, the Arabian Sea, and especially the Mediterranean Sea (Rodriguez et al. 2022). Areas with knowledge gaps in Hg exposure information are across the South Pacific (Rodriguez et al. 2022).

The lack of a standardized sampling methodology poses challenges in utilizing these data for global-scale environmental monitoring programs. The establishment of a standardized sampling methodology is imperative for future studies. Furthermore, using the carapace, as documented by Barrios-Rodríguez et al. (2023), is essential to standardize the collection point of scute samples due to differences among the vertebral, costal, and marginal scutes. The application of these guidelines together with a thorough assessment of characteristics such as diet, distribution area, contamination sources near foraging areas, and the age range of the individuals under study would allow a distinct comprehension of the origin of Hg in sea turtles. This approach would also aid in delineating species’ disparities and investigating worldwide Hg contamination.

#### Seabirds – ecological health assessment

##### Rationale and caveats for biomonitoring

Seabirds occupy a broad range of trophic levels, but most seabirds occur high in the food web and therefore biomagnify elevated concentrations of Hg and are therefore important bioindicators (Monteiro and Furness 1995; Mallory and Braune 2012; Elliott and Elliott 2013; Provencher et al. 2014; Gilmour et al. 2019a, b; Albert et al. 2021). Seabirds permit Hg monitoring across large geographical scales and variations within the same species or family over longitudinal (e.g., brown noddy, *Anous stolidus*, for the intertropical zone, and Adélie penguin, *Pygoscelis adeliae,* for the circumantarctic area (Cusset et al. 2023) or latitudinal scales (e.g., skuas and jaegers for both southern and northern hemispheres; Albert et al. 2022; Carravieri et al. 2017; Fleishman et al. 2019). The use of different tissues with different integration time (e.g., generally blood reflects short-term exposure and adult feathers reflect long-term exposure) constitute relevant approaches to provide integrated values of Hg contamination over different time scales. The variation in Hg contamination in seabird tissues can thus reveal differences in the degree of contamination between major ocean basins, as well as latitudinal gradients of contamination within basins, and trends at a series of spatial and temporal scales.

**Fig. 15:**
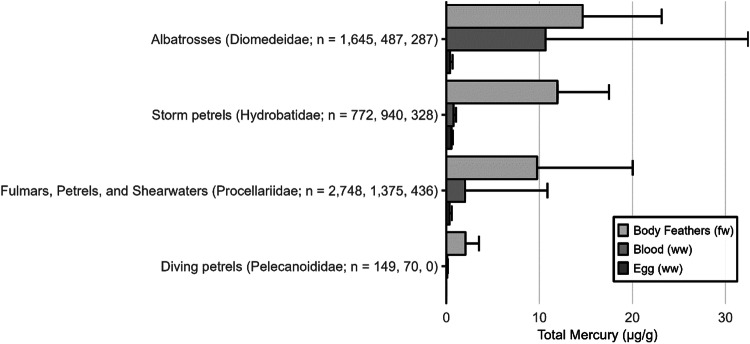
Global Hg concentrations in seabirds. Stacked bars illustrate the arithmetic mean ± SD of global total Hg concentrations (µg/g, ww) in three tissues: light gray (fw in feathers; n = 5314), gray (ww in blood; n = 2872) and dark gray (ww in eggs; n = 1051) of four seabird families within the Order Procellariformes

Mercury concentrations in adult body feathers generally reflect the bird’s exposure since the last molt. The significant remobilization of Hg stored in internal tissues during molting leads to depuration of up to 90% of the Hg stored since the last molt (Agusa et al. 2005a, 2005b; Braune 1987; Honda et al. 1986). Interpretation of feather Hg concentrations can be challenging as levels can relate to MeHg dietary uptake from different foraging sites from where the bird has been (breeding, migratory, or wintering) and prey items, level of stress, and age. Mercury biomonitoring deliberations could consider tracking adult feather, blood and egg Hg concentrations in Procellariforms or other seabirds (Fig. 15) and incorporate differences in ocean basins, proper interpretation of tissue types as associated with species’ ecology, movements, and diet. Tracking selenium body burdens is also important when interpreting toxicity of MeHg to seabirds (Cruz-Flores et al. 2024).

Use of feather Hg concentrations from chicks is difficult as they are challenging to interpret without accounting for age because rapid chick growth rates disassociate Hg in feathers with internal tissues (Peterson et al. 2019) as well as carryover from adult body burdens (Carravieri et al. 2023). However, it provides the advantage to reflect local contamination from the food brought by the adults to feed their chicks (Blévin et al. 2013). Mercury from multiple tissue types can be converted into a standard unit such as blood equivalencies to assess changes over time in seabirds (Pollet et al. 2022).

Due to their foraging strategies, behavioral ecologies, and life-history traits (e.g., breeding sequence, molting strategies, foraging ranges, migration patterns), seabirds generally have elevated body burdens of Hg that can ultimately impact their fitness, reduce their reproductive capacity and affect their population sizes over time (Braune et al. 2006; Tartu et al. 2013; Goutte et al. 2014a, b; Bond et al. 2015; Bauch et al. 2022; Chastel et al. 2022). For instance, particular concerns arise for adverse Hg effects on the increasingly rare ivory gull (*Pagophila eburnea*, Braune et al. 2006; Bond et al. 2015) and there is evidence of physiological harm to the near-threatened, black-vented shearwater (*Puffinus opisthomelas*; Soldatini et al. 2020) and sublethal effects on immunity, liver function and breeding parameters in an Antarctic seabird – the brown skua (*Stercorarius antarcticus*) (Ibañez et al. 2024). Many studies have focused on seabird Hg tissue concentrations from tropical to polar regions and from coastal to oceanic zones, covering most of the world’s oceans. Nevertheless, the South Pacific and other areas of the Southern Hemisphere appear to be less documented while the Arctic has received special attention, partly because seabirds are a food resource for human populations (AMAP 2011; Albert et al. 2021; Schneider et al. 2023).

The GBMS database shows that seabirds exhibit a wide range of Hg concentrations across tissue types (e.g., feathers, blood, eggs), driven by spatial differences, trophic ecology as well as phylogeny. For example, penguins generally have the lowest Hg concentrations in feathers, blood, and eggs, whereas Procellariiforms (e.g., petrels, shearwaters, storm-petrels, and albatrosses) generally have the highest Hg body burdens (Fig. 15). Because of their large diversity, the Procellariiforms are a well-studied group and display a wide range of tissue Hg concentrations that reflect phylogenetic and physiological differences with albatrosses exhibiting the highest Hg concentrations (Muirhead and Furness 1988; Stewart et al. 1999; Anderson et al. 2010; Tavares et al. 2013; Bustamante et al. 2016; Cherel et al. 2018; Mills et al. 2020).

The most important factor for predicting seabird Hg exposure is their foraging ecology (e.g., Carravieri et al. 2014). Because seabirds use a wide range of habitats, from the coastal margins to the open ocean, species or individuals with differing foraging behaviors can reflect Hg contamination from different parts of the ecosystems both horizontally (e.g., coastal and oceanic food webs) and vertically (i.e., benthic, epipelagic, and mesopelagic food webs). Therefore, the study of a group of seabirds with contrasting ecology from the same region allows determination of MeHg availability for multiple marine zones and thus provides a more holistic view (Ochoa-Acuña et al. 2002; Bond and Diamond 2009; Stenhouse et al. 2018; Pollet et al. 2023). For example, crustacean-feeding seabirds have lower Hg exposure than cephalopod- and fish-feeders (Carravieri et al. 2014) and epipelagic seabirds have lower Hg exposure than those relying on mesopelagic prey (Ochoa-Acuña et al. 2002; Furtado et al. 2021). Seabirds of the highest trophic levels (e.g., albatrosses or skuas) are therefore at risk to the effects of MeHg toxicity that are associated with potential long-term population declines and potentially can be impacted by co-occurring contaminants such as persistent organic pollutants (Goutte et al. 2014a, b) or by deficiency in selenium, which protects against Hg toxic effects (Manceau et al. 2021b).

Based on the GBMS database, storm-petrels breeding in the Northern Hemisphere have feather Hg concentrations that are ten-fold higher (13.8 ± 3.7 µg/g) than populations breeding in the Southern Hemisphere (2.1 ± 1.8 µg/g). Such a difference is not found for the Procellariidae (8.4 ± 7.4 vs 11.1 ± 12.5 µg/g, respectively). Differences between hemispheres could be explored further using seabirds with similar trophic ecology as well as close phylogeny.

#### Loons/Divers

##### Rationale and caveats for biomonitoring

**Fig. 16:**
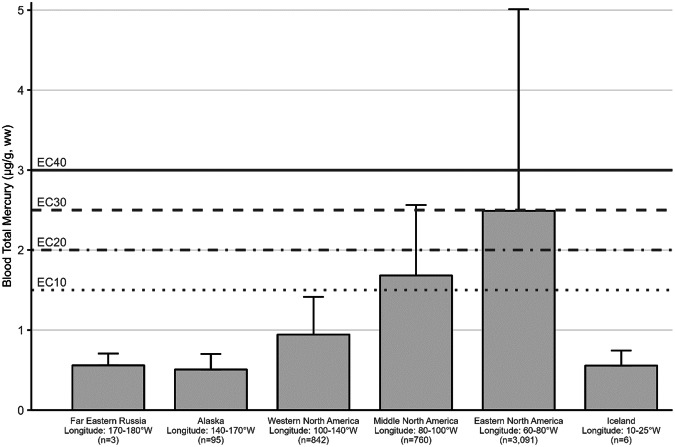
Mercury concentrations in loons (n = 4797). Gray bars illustrate the arithmetic mean ± SD of global total Hg concentrations (µg/g, ww) in common and yellow-billed loon indexed blood across parts of the northern hemisphere (from 10–180° W) from the GBMS database. Environmental concentrations that have a 10% or more reduction in reproductive success (e.g., EC10) are based on Table 3b

Species within the Order Gaviiformes (loons or divers) are piscivores that breed on freshwater ponds and lakes in temperate and Arctic ecosystems of the Northern Hemisphere. In the winter, all loon species migrate to marine ecosystems (with parts of some populations overwintering on freshwater lakes). The two largest loon species (common loon and yellow-billed loon, *Gavia adamsii*) are obligate piscivores and accordingly have some of the highest average Hg body burdens of birds in the world. Mercury biomonitoring deliberations could consider tracking adult and egg Hg concentrations in all loon/diver species, with an emphasis on the two largest species (Fig. 16) and account for differences in body size, age, sex, prey availability and waterbody type (e.g., lake or reservoir).

Loons have been used as bioindicators of MeHg availability in both their breeding and wintering areas for several decades for the common loon (Meyer et al. 1998; Scheuhammer et al. 1998; Evers et al. 1998, 2003, 2008, 2011a; Burgess et al. 2005; Jackson et al. 2016; Schoch et al. 2020) and more recently for the yellow-billed loon (Evers et al. 2014) and red-throated loon (*Gavia stellata*; Eriksson et al. 1992; Schmutz et al. 2009). The effects of Hg on loon reproductive success are well established (Burgess and Meyer 2008; Evers et al. 2008, 2011b; Depew et al. 2012b) and are used as benchmarks for evaluating ecological concern in piscivorous birds.

In Canada, the common loon and its prey are being monitored to evaluate the success of national regulatory standards to reduce Hg emissions (Scheuhammer et al. 2016); and recent findings indicate continued adverse reproductive impacts from Hg across Canada (Tozer et al. 2013), including an annual loss of 0.01 fledged chicks per territorial pair over the past 40 years in Ontario due to MeHg burden in prey (Bianchini et al. 2020). Loons are being used as a standard bioindicator across the United States as well (Evers et al. 1998, 2003). Based on original research (Evers et al. 1998), and supported by other datasets over the past two decades, the GBMS data was used to demonstrate a west to east increasing gradient of MeHg availability in lakes within temperate and boreal forest ecosystems, with Alaskan breeding populations having the lowest Hg concentrations and eastern North American populations the highest (Fig. 16). The smaller loon/diver species, while less piscivorous and having lower Hg concentrations (Jackson et al. 2016), remain potential bioindicators for MeHg availability across their ranges, especially in Scandinavia (Eriksson et al. 1992; Eriksson 2015).

#### Raptors

##### Rationale and caveats for biomonitoring

Birds of prey, or raptors, comprise a large and varied group of birds generally characterized as predators. Several raptors at the species (e.g., osprey, *Pandion haliaetus*) or genus (*Haliaeetus* eagles) levels have near global distribution and so are commonly used in spatial and temporal contaminant monitoring efforts (Bowerman et al. 2002; Hollamby et al. 2004; Weech et al. 2006; Grove et al. 2009; Henny et al. 2009; DeSorbo et al. 2018; Sun et al. 2019; Bjedov et al. 2023). In breeding areas, developing nestlings of many raptor species are often more efficiently captured for tissue sampling than resident adults – even though chick feather Hg concentrations are challenging to interpret. Nestlings can be effective for spatial and temporal Hg monitoring since they reflect exposure over a well-defined period of nesting development (e.g., six weeks), while adult exposure is more easily linked to risk (Ackerman et al. 2016; Evers 2018).

**Fig. 17:**
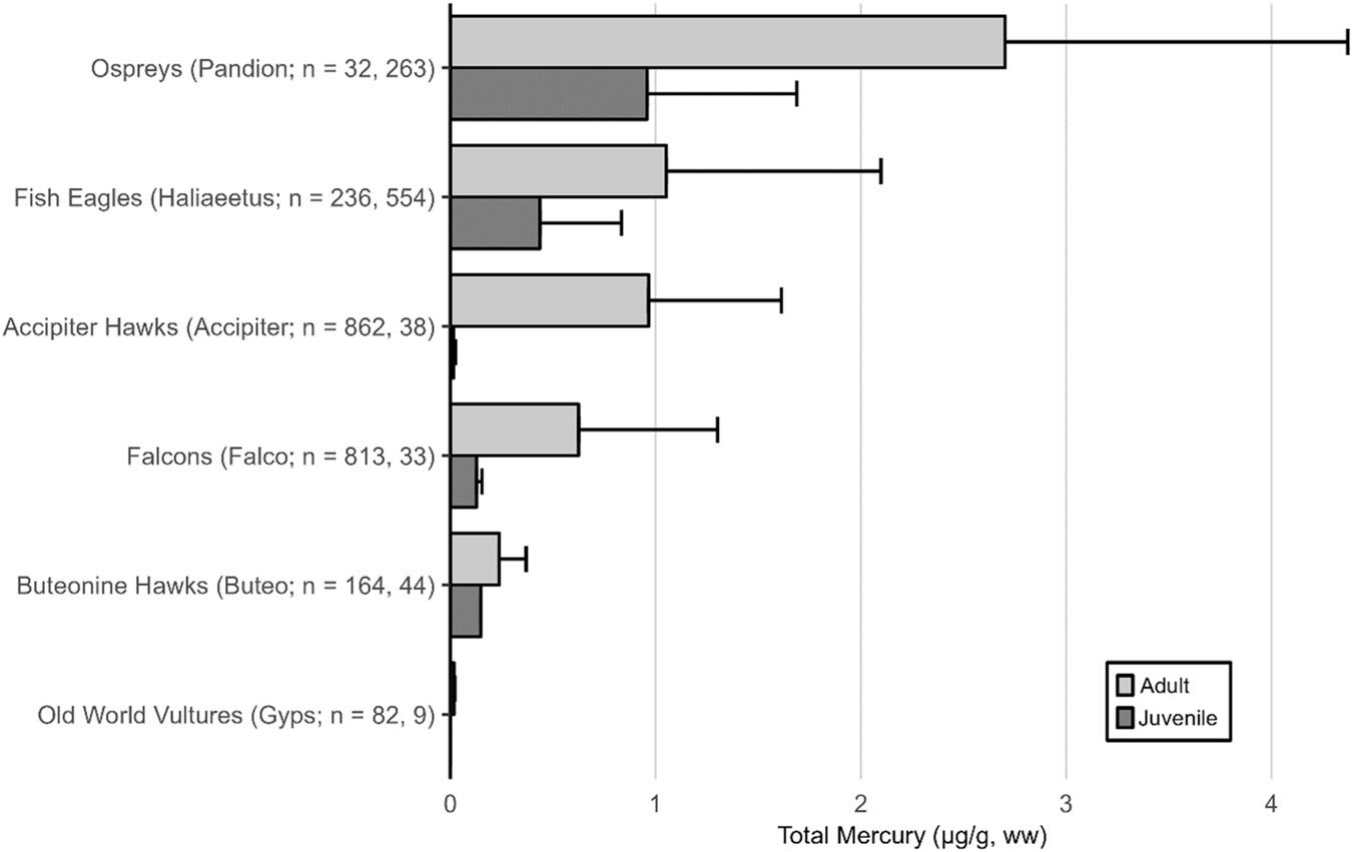
Global Hg concentrations in raptors (n = 3130). Stacked bars represent the global arithmetic mean ± SD of total Hg concentrations in blood (µg/g, ww) of adult (light gray) and juvenile (dark gray) age classes of six selected Genera of raptors

Adult raptors consistently exhibit higher Hg concentrations than nestlings, largely due to nestlings’ ability to depurate MeHg into growing feathers (Ackerman et al. 2011) and the bioaccumulation that outpaces depuration and demethylation, especially for older individuals. In both age groups, individuals sampled in association with nesting territories generally reflect MeHg exposure in the food web from that territory (Bowerman et al. 1994; DeSorbo et al. 2018). Greater MeHg availability associated with increasing elevational gradients (DeSorbo et al. 2020) or varying across subpopulations (Sun et al. 2019) have also been shown for *Haliaeetus* eagles. Mercury biomonitoring deliberations could consider tracking adult, nestling, and egg Hg concentrations in piscivorous raptors (e.g., *Pandion* and *Haliaeetus* species) and some terrestrial raptors (e.g., *Accipiter* and *Falco* species) (Fig. 17) as long as an accounting of differences in body size, dietary habits, and habitat type use among species is considered.

Piscivorous raptors, namely osprey and *Haliaeetus* eagles, are well-suited for Hg biomonitoring within and across multiple habitat types (marine, estuarine, river, lake) (Jackson et al. 2016; Rumbold et al. 2017; DeSorbo et al. 2018; Sun et al. 2019). *Haliaeetus* eagles are key sentinels in environmental programs used to monitor spatial and temporal Hg exposure patterns in North America, particularly in the U.S. Great Lakes ecosystem (Bowerman et al. 2002) and in Fennoscandia (Sun et al. 2019; Gómez-Ramírez et al. 2023). A study of bald eagles (*Haliaeetus leucocephalus*) in the Great Lakes of the United States found evidence that Hg adversely affects a proportion of this population (Rutkiewicz et al. 2011); in that study, 14–27% of individuals sampled were exposed to Hg at concentrations associated with subclinical neurological damage.

The GBMS database reveals that piscivorous raptors such as osprey and *Haliaeetus* eagles tend to exhibit the highest adult blood Hg concentrations among raptors (Fig. 17). Raptor species specializing in bird prey (e.g., many *Accipiter* and *Falco* spp.) generally have higher average Hg concentrations (Keyel et al. 2020) than those predominantly targeting small mammals (e.g., *Buteo* and *Circus* spp.) (Bourbour et al. 2019), while obligate scavengers are generally exposed to low levels of Hg (Herring et al. 2018).

While piscivorous raptors were predominantly emphasized in past Hg biomonitoring, recent studies show that MeHg is also prevalent in terrestrial-based food webs, and that invertivorous birds (Passeriformes) can have elevated MeHg concentrations (Jackson et al. 2011a, 2015; Evers 2018) that can result in levels of concern in raptors such as Accipiters and falcons that feed on those birds (Newton et al. 1999; Barnes and Gerstenberger 2015, 2019; Bourbour et al. 2019; Keyel et al. 2020). Studies that documented sublethal dietary MeHg exposure in captive American kestrels (*Falco sparverius*) demonstrated neurotoxic impacts (Bennett et al. 2009) and reproductive harm (Albers et al. 2007) and provide threshold benchmarks for wild populations. Other foodwebs of raptors should also be considered and mindfully assessed – for example, the trophic transfer in a novel foodweb of some striated caracara populations (*Phalcoboenus australis*), which focused on southern rockhopper penguins (*Eudyptes chrysocome*) for parts of the year, resulted in highly elevated Hg concentrations (Balza et al. 2021).

#### Freshwater birds

##### Rationale and caveats for biomonitoring

**Fig. 18:**
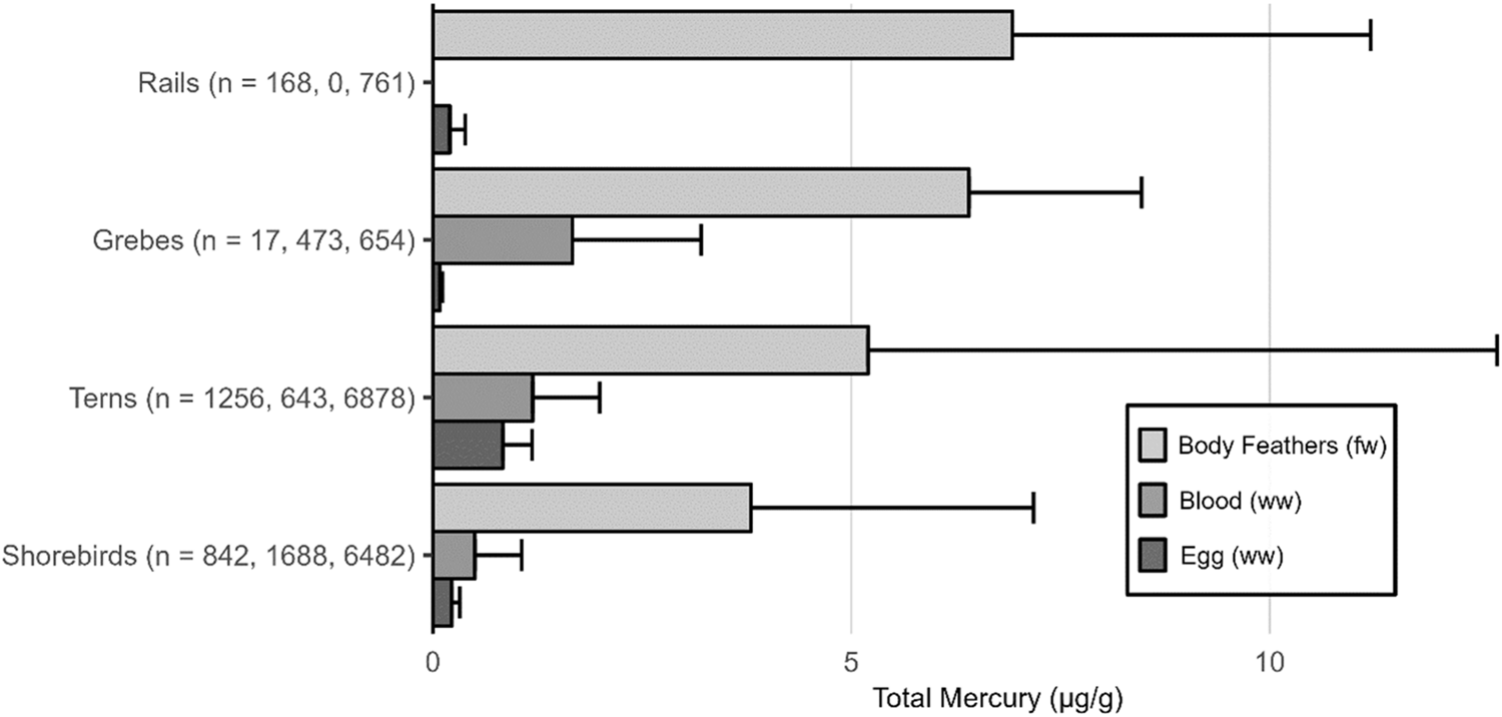
Mercury concentrations in four general freshwater bird groups (i.e., rails, grebes, terns, and shorebirds; n = 19,862). Bars illustrate the arithmetic mean ± SD of total Hg concentrations (µg/g) in body feathers (fw), blood (ww), and eggs (ww) from the GBMS database

As conferred, freshwater habitats are often conducive to MeHg production and bioaccumulation, and freshwater birds are among the most numerous non-marine birds exposed to naturally elevated levels of MeHg contamination. There are numerous freshwater bird species that are appropriate for long-term sampling for environmental Hg biomonitoring (Fig. 18). Among these, Forster’s terns (*Sterna forsteri*), Caspian terns (*Hydroprogne caspia*), and Clark’s grebes (*Aechmophorus clarkii*) are the most Hg contaminated bird species in western North America (Ackerman et al. 2016). Shorebirds can also be highly exposed to Hg contamination in breeding (Ackerman et al. 2007; Eagles-Smith et al. 2009a; Hargreaves et al. 2010; Perkins et al. 2016; Chastel et al. 2022; Perkins et al. 2023) and wintering areas (Lucia et al. 2014; Burger et al. 2018; Correia et al. 2023), and small differences in foraging strategies can result in large differences in Hg concentrations among species (Ackerman et al. 2007).

Other freshwater and estuarine birds of interest for Hg biomonitoring include kingfishers (Evers et al. 2005; Zamani-Ahmadmahmoodi et al. 2009; Hurtado et al. 2023; Oliveira et al. 2023; Pisconte et al. 2024), rails (Cumbee et al. 2008; Tsao et al. 2009; Ackerman et al. 2012; Casazza et al. 2014), and wading birds such as herons (Goutner et al. 2001), night-herons (Henny et al. 2002, 2007), ibis (Mullié et al. 1992; Klekowski et al. 1999; Heath and Frederick 2005), and egrets (Frederick et al. 1999, 2002; Sepúlveda et al. 1999a, 1999b; Rumbold et al. 2001; Zamani-Ahmadmahmoodi et al. 2010).

Documented adverse effects of Hg are known for shorebirds (Ackerman et al. 2008; Eagles-Smith et al. 2009a; Lucia et al. 2012), terns (Hoffman et al. 2011; Herring et al. 2010; Braune et al. 2012), rails (Heinz et al. 2009; Ackerman et al. 2012), and wading birds (Heinz et al. 2009; Sepúlveda et al. 1999a, 1999b; Frederick and Jayasena 2011) and therefore provide baseline information that can support the interpretation of meaningful Hg trends. As with other bird species, monitoring programs for freshwater birds could include tracking adult Hg concentrations in blood and eggs and should account for differences in sexes, sampling dates, locations, and habitats.

#### Landbirds

##### Rationale and caveats for biomonitoring

Many species of invertebrate-eating birds (herein called landbirds) are also at elevated risk to Hg exposure. Remarkably, landbirds can exhibit higher tissue Hg concentrations than fish-eating birds within the same ecosystem (Evers et al. 2005; Kopec et al. 2018; Sayers et al. 2023). They may also be more sensitive to MeHg, resulting in a higher likelihood of adverse impacts on reproductive success (Heinz et al. 2009; Jackson et al. 2011b; Whitney and Cristol 2017). An increasing number of studies characterizing Hg exposure in songbirds (Passeriformes) are demonstrating that certain clades are at higher risk than others, based largely on foraging behavior, and breeding habitats (Cristol and Evers 2020). Generally, gleaning, flycatching, and “predatory” songbirds that breed in wetland habitats (Edmonds et al. 2010; Jackson et al. 2011a, 2015, 2020; Hartman et al. 2013; Ackerman et al. 2016; Pacyna et al. 2017; Ackerman et al. 2019), including estuaries (Lane et al. 2011; Kopec et al. 2018; Sayers et al. 2021), rice fields (Abeysinghe et al. 2017; Xu et al. 2024) and tropical evergreen forest floodplains (Sayers et al. 2023) are at highest risk of Hg exposure, especially species that forage on predaceous arthropods such as spiders (Cristol et al. 2008; Janssen et al. 2023). The availability of MeHg to tropical resident songbirds (and other landbird groups) is increasingly becoming more evident and are more elevated for certain foraging guilds and habitat types (Lane et al. 2013; Townsend et al. 2013). Sayers et al. (2023) analyzed over 1800 individual neotropical Passeriformes and found warblers, woodcreepers, antbirds, and wrens to have the most elevated body burdens of Hg.

**Fig. 19:**
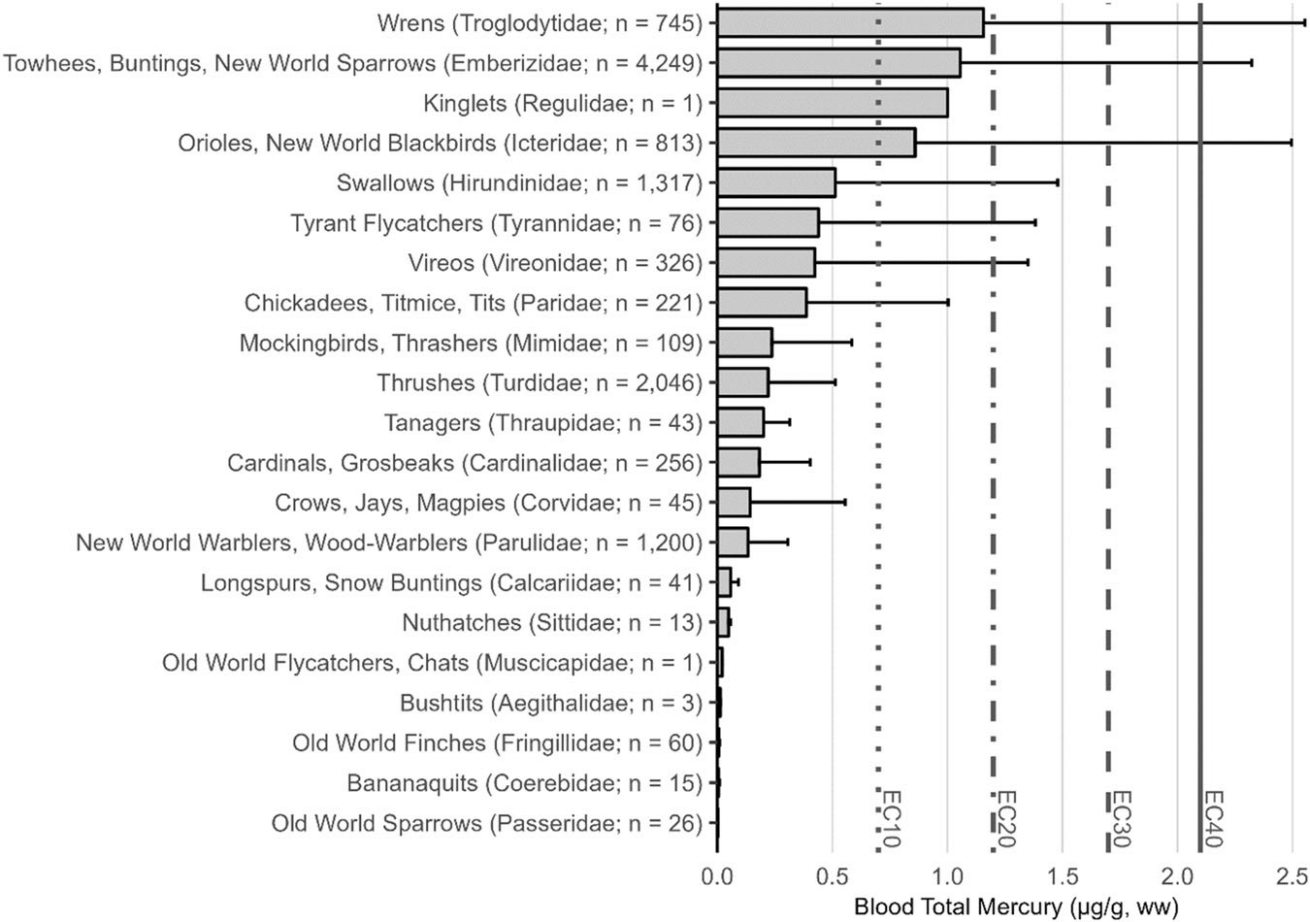
Mercury concentrations in in 21 families of songbirds in the United States (n = 11,606). Gray bars illustrate the arithmetic mean ± SD of total Hg concentrations in blood (µg/g, ww) from the GBMS database. For EC level definitions see Table 3b

Mercury biomonitoring deliberations could consider tracking adult Hg concentrations in blood or feathers of landbirds, with an emphasis on families known to exceed thresholds of interest (0.7 µg/g blood Hg), including 8 of 21 (38%) passerine families (see Table 3b for thresholds and Fig. 19 for Hg profiles) and incorporate differences in body size, prey availability and habitat type. A similar analyses of passerine blood Hg data collected within the new initiative called Tropical Research for Avian Conservation and Ecotoxicology (TRACE) Initiative found 23 of 51 (45%) Neotropical families with individuals exceeding thresholds of interest (Sayers et al. 2023).

Songbird species that spend most of their annual life cycle within wetland-oriented ecosystems and that migrate long-distances (e.g., neotropical migrants or Palearctic migrants) may also be at great risk of chronic Hg exposure adversely impacting migratory and reproductive success (Jackson et al. 2011b; Varian-Ramos et al. 2014; Ma et al. 2018a). New findings on elevated Hg exposure and migration physiology/behavior indicate significant adverse impacts are possible, especially for long-distance migrants that may experience decreased flight endurance (Seewagen et al. 2016, Seewagen 2020; Ma et al. 2018b; Branco et al. 2022) that could also be related to increasing flight feather asymmetry in high Hg individuals (Herring et al. 2017). Models including ones predicting Hg exposure to neotropical migrants now demonstrate that warblers (Paruilidae) are a particularly vulnerable group (Sayers et al. 2023). The GBMS data can be used for quantifying broad trends in different taxa – such as the higher Hg burdens in some songbird families (e.g., Troglodytidae, Emberizidae, and Icteridae)) versus those that likely forage more on seeds and berries from upland habitats (e.g., Cardinalidae, Calcariidae and Fringillidae).

#### Marine mammals – toothed and baleen whales, pinnipeds, and polar bears

##### Rationale and caveats for biomonitoring

**Fig. 20:**
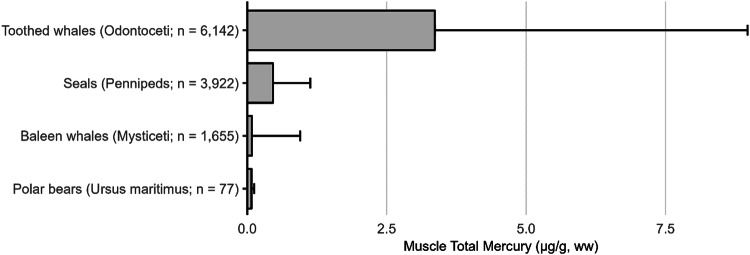
Global Hg concentrations from the GBMS database for marine mammals (n = 11,796). Gray bars illustrate the arithmetic mean ± SD of muscle total Hg concentrations (µg/g, ww) in four groups of marine mammals

Recent studies suggest that Hg concentrations in marine mammals have increased approximately 20 times relative to pre-industrial concentrations (Dietz et al. 2009; AMAP 2011). Of the four broad groups of marine mammals (toothed and baleen whales, seals and other pinnipeds, and polar bears, *Ursus maritimus*), toothed whales (Odontoceti) generally have the highest Hg body burdens (Fig. 20). Toothed whales include about 88 species of whales, dolphins, and porpoises and prey on higher trophic level organisms than baleen whales. Seals and other pinnipeds, such as walruses (*Odobenus rosmarus)* and Stellar’s sea lions (*Eumetopias jubatus*), are distributed around the world’s oceans and can also serve as biomonitoring options. Mercury biomonitoring deliberations should consider tracking muscle Hg concentrations in toothed whale and various pinniped species (Fig. 20) and account for differences in species, home range, ecology, and interpretation of tissue types. Lastly, Hg biomonitoring in polar bears has been used to provide insight into temporal changes in MeHg availability in Arctic ecosystems and should also be included in Arctic biomonitoring programs.

Compared to baleen whales, toothed whales generally forage higher on the food web and as a result average 47 times higher Hg concentrations (Fig. 20). Average Hg concentrations in many species of toothed whales are highly elevated — and potentially high enough to cause physiological, behavioral, or reproductive harm (Wagemann et al. 1998; Wagemann and Kozlowska 2005; Dietz et al. 2013, 2022).

The species of greatest concern from Hg contamination, based on existing GBMS data, include the false killer whale, killer whale, short-finned pilot whale, pygmy killer whale, striped dolphin, Risso’s dolphin, bottlenose dolphin, and various species of beaked whales (all with average Hg concentrations greater than 5.0 µg/g, ww in the muscle; Fig. 13). One reason for these elevated concentrations, in addition to their high trophic position, is that cetaceans cannot depurate MeHg through hair formation such as in polar bears and seals or through feathers by birds (Dietz et al. 2013). In general, Hg concentrations observed in small toothed whales (e.g., the Franciscana and Guiana dolphins) are considered low, and less relevant, when compared to those found in larger odontoceti, especially delphinids. However, it is important to note that these small dolphins weigh almost half, or less, than the weight of most delphinids (e.g., bottlenose dolphin) and therefore, because of increased metabolism in smaller species, the doses of Hg can be similar when accounting for individual size. Elevated concentrations of muscular Hg have been observed in endangered species of odontoceti, representing yet another risk factor impacting their populations (Manhães et al. 2022).

Some pinnipeds, such as the ringed seal are considered medium-trophic level predators and rely on large zooplankton, epibenthic and under-ice crustaceans, and pelagic and demersal fishes including Arctic cod and polar cod (*Boreogadus saida* and *Arctogadus glacialis*, respectively) for their diet (e.g., Lowry et al. 1980; Weslawski et al. 1994; Wathne et al. 2000). Other species, including bearded seals (*Erignathus barbatus*) and walrus, are more omnivorous, with diets that include benthic invertebrates (Born et al. 1981). This variation in diet influences the level of Hg exposure. Spatial variation in Hg concentrations in ringed seals suggests that the central and western Canadian Arctic is higher in Hg than other Arctic regions including Alaska, Greenland, Norway, and Russia (Rigét et al. 2005; Brown et al. 2016). Mercury in seals has also been shown to vary seasonally and is linked to variations in sea ice cover. Periods of greater sea ice are related to higher Hg concentrations, in part because seals are more reliant on fish. During warmer seasons when sea ice is reduced, seals can forage on a broader range of prey items, effectively reducing their exposure to Hg (Houde et al. 2020).

Polar bears live most of their lives on sea ice, hunting pinnipeds and other marine mammals. Seasonal variation in polar bear Hg exposure is related to sea ice fluctuations and availability of prey (Morris et al. 2022b). Higher Hg concentrations have been observed in spring and autumn, whereas low Hg concentrations have been observed during winter months when prey are more difficult to locate (AMAP 2011). Research has shown sex-specific differences in Hg levels but also Hg sensitivity (Bechshoft et al. 2016). While adult female polar bears have been found to have higher Hg concentrations in hair than adult males, adult males appear to be more sensitive with regards to Hg-related health responses. Also, in polar bear offspring, males were found to have higher Hg levels (Bechshoft et al. 2016). The different Hg concentrations in adult polar bears may be due to sex-specific variability in the diet. It has been suggested that female bears generally target smaller prey items, such as ringed seals (mostly fish-eaters) that are higher in Hg while males prey upon larger pinnipeds, such as bearded seals and walruses, that primarily forage on mollusks (Bechshoft et al. 2016). Males may also have reduced Hg exposure because of greater consumption of blubber, which is low in MeHg. Elevated Hg concentrations in female polar bears and the transfer of MeHg to the polar bear fetus represent a potential long-term conservation concern that could affect future populations of polar bears (Bechshoft et al. 2016). Like persistent organic pollutants, MeHg is transferred from mother to fetus in polar bears and the potential impacts require attention and further research (Cardona-Marek et al. 2009; Knott et al. 2012; Bechshoft et al. 2016).

As observed in ringed seals, spatial variation in Hg concentrations in polar bears indicates hotspot areas in the Canadian Arctic Archipelago and northwestern Greenland (e.g., AMAP 1998; Brown et al. 2016; Dietz et al. 1998, 2000, 2013; Routti et al. 2011, 2012; AMAP 2021, Dietz et al. 2022). These hotspots have recently been linked to the presence of elevated MeHg in the upper 400 m of the water column (AMAP 2021, Dietz et al. 2022).

## Discussion

The GBMS database compiles relevant data at multiple geographic levels that can be useful platforms for understanding the breadth of existing biotic Hg data around the world. As illustrated above, the existence of a standardized, queryable database allows for data to be analyzed according to a variety of relevant factors. Geographically, the world’s ocean basins and continents are suitable regional scales, although further reduction in area would likely be more manageable and insightful. Taxonomically, bioindicators of human exposure and/or ecological health that are important for policy decisions within the Minamata Convention are now identified, although their selection depends on many factors identified in this paper.

**Fig. 21:**
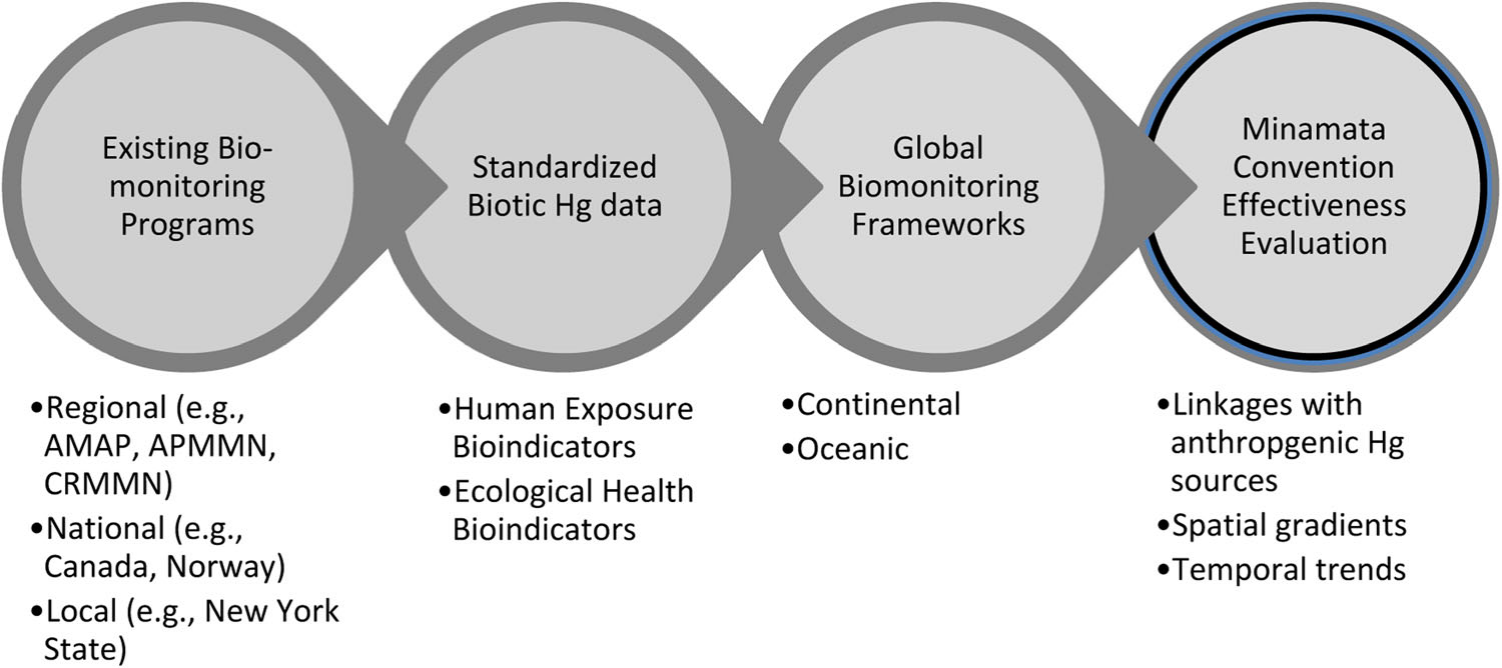
Conceptual diagram of the relationships and process for integrating existing data from biomonitoring programs (e.g., Arctic Monitoring and Assessment Program [AMAP], Asia Pacific Mercury Monitoring Network [APMMN], and the Caribbean Region Mercury Monitoring Network [CRMMN]) to design appropriate biomonitoring frameworks to inform global policy objectives of the Minamata Convention

The relationships among past and ongoing biomonitoring programs and databases (that exist at multiple spatial and jurisdictional levels), and how they can support the flow of data that are both comparable and sufficient to meet specified requirements are the core of international policy-related assessments. In this synthesis, the analyses have focused on assembling information currently available in the public domain into relevant categories that help inform the overarching monitoring-related interests within the Minamata Convention. This analysis provides insight into gaps in spatial coverage of data around the world, which in turn can facilitate the prioritization of cost-efficient and strategic global biomonitoring frameworks. An example would be a global monitoring framework distributed across continents and ocean basins that ultimately can reflect changes in environmental Hg loads directly related to human health and the environment (Fig. 21).

### Existing biomonitoring programs

Biomonitoring programs exist worldwide, particularly in developed countries in the northern hemisphere (e.g., U.S., Canada, across several European countries, South Korea, and Japan). However, only a few programs track long-term patterns of both environmental inorganic Hg (e.g., air) and the bioavailability of MeHg (e.g., fish). Existing biomonitoring programs were identified in a United Nations Environment Programme review (UNEP 2016). For the UNEP compilation, Hg biomonitoring programs were identified following a formal global request. Responses were compiled and provide the most up-to-date record of existing local, regional, and global abiotic and biotic Hg monitoring programs. These include programs underway within many national networks, including initiatives in Brazil, Canada, Colombia, Japan, Norway, Poland, South Korea, Spain, Sweden, United Kingdom, and the United States as well as a few regional and global networks (UNEP 2016). Data from these monitoring programs are in the GBMS database if they were published in peer-reviewed journals.

Some parts of the world are covered by long-established regional monitoring programmes. Examples include the Arctic, through the Arctic Monitoring and Assessment Programme (AMAP 2021) which is largely based on national programs such as Canada’s Northern Contaminants Program (NCP) (Chételat et al. 2015) and comparable programmes in other Arctic countries. The AMAP assessments also utilize data from other relevant monitoring initiatives such as the ARCTOX program for tracking Hg in seabirds (Albert et al. 2019). Other established international monitoring programmes exist for the European regional seas: the Northeast Atlantic (OSPAR Convention), Baltic Sea (HELCOM Convention) and Mediterranean Sea (Barcelona Convention).

There are several programs in the temperate regions of the United States (e.g., the U.S. Environmental Protection Agency’s seafood Hg monitoring program), European national programs (e.g., ROCCH, Réseau d’Observation de la Contamination Chimique), and Japan (e.g., Japanese National Institute for Environmental Studies). However, in developing countries and countries in economic transition there are fewer national or regional long-term initiatives. There are very few long-term Hg biomonitoring efforts in tropical biomes that include global development priorities such as Small Island Developing States or Least Developed Countries.

A review of the geographical coverage of Hg biomonitoring networks reveals a general lack of regional initiatives around the world, especially in Africa and Australia (UNEP 2016), which is notably similar to coverage of Hg concentrations in humans as well (Basu et al. 2018, 2023). Most Asian countries are minimally involved with national initiatives to monitor Hg levels in biota, notable exceptions being Japan and South Korea where more extensive programs exist and may be expanded by the Asia Pacific Mercury Monitoring Network (APMMN; www.apmmn.org). Conversely, Hg biomonitoring is ongoing in many countries within Europe, Oceana and across the Western Hemisphere. Also, Environmental Specimen Banks can be used as monitoring tools to provide long-term trends for contaminants in the environment, including Hg (Day et al. 2014; Paulus et al. 2015; Qiu et al. 2015; García-Seoane et al. 2017). Data that were generated from these monitoring programs and were published in peer-reviewed journals are now housed in the GBMS database.

To provide sustainable and long-term biomonitoring capacity in key regions (e.g., Arctic, tropical areas associated with ASGM, and Small Island Developing States), it could be valuable to focus on stabilizing and expanding existing local and national initiatives that use sample sizes with sufficient statistical power for understanding spatial gradients (such as biological MeHg hotspots; Evers et al. 2011b) and temporal trends (Bignert et al. 2004; Rigét et al. 2011; Morris et al. 2022a). Moreover, international collaboration and coordination among national programs could help create harmonized regional approaches and integrate biomonitoring activities into an interdisciplinary framework to assess environmental and human health risk. This creates an efficient, hierarchical framework whereby regional efforts can then be amalgamated to represent global spatio-temporal patterns. Mercury scientists from around the world, comprising the Open-Ended Science Group (OESG) and Technical Experts, are currently collaborating to compile global data to support the first effectiveness evaluation required by the Minamata Convention (UNEP 2019b, 2022).

A recent example of robust Hg monitoring efforts happening at a local level that are linked to local and regional policy regulations to lower Hg emissions and releases from anthropogenic sources is within New York State in the United States. Here, over 47,000 Hg concentrations have been collected in biota over nearly a 50-year period alongside detailed measurements in atmospheric deposition (both wet and dry) across the state (Evers et al. 2020). However, it was not until this information was collected into a standardized database designed to answer specific, policy-related questions that it was possible to adequately assess the status and trends of Hg contamination and risk, and to design more cost-effective monitoring programs in the future. By assembling long-term tracking using standard tissues and species of fish and birds, it was possible to identify important declines in environmental Hg loads (Adams et al. 2023). Several similar efforts have assembled vast amounts of Hg data into U.S. regional databases and serve to demonstrate the value of such synthesis for the northeastern United States and eastern Canada (Evers and Clair 2005), Great Lakes Region of the United States and Canada (Evers et al. 2011a), and western United States and Canada (Eagles-Smith et al. 2016b).

Although not included in the original Article 19 list of bioindicators, some invertebrate taxa are emerging as effective tools for tracing temporal and spatial trends in Hg risk to ecosystems. In particular, the interagency (US Geological Survey, National Park Service, US Fish and Wildlife Service) Dragonfly Mercury Project (Eagles-Smith et al. 2020) is the largest ongoing (2011-present) Hg monitoring network in the United States. Mercury in dragonfly larvae have been shown to be strongly correlated with those in paired fish and amphibians from the same water body (Eagles-Smith et al. 2020), as well as biogeochemical factors associated with Hg cycling (Willacker et al. 2023). As such, these bioindicators can also be used to better understand the factors and mechanisms influencing MeHg bioavilability to food webs. Additionally, an Integrated Impairment Index has been developed for dragonfly larvae that equates their Hg concentrations to various ecosystem health risk benchmarks (Eagles-Smith et al. 2020).

### Linking existing biota Hg data and biomonitoring programs with objectives of the Minamata Convention

Biomonitoring programs and summarized biotic Hg data can provide information to respond to three of the four identified policy questions under the Effectiveness Evaluation process established in Article 22 of the Minamata Convention (UNEP 2019b):

i. Have the actions taken by Parties of the Minamata Convention resulted in changes in mercury supply, use, emissions, and releases into the environment?

ii. Have those changes resulted in changes in levels of mercury in the environment, biotic media and vulnerable populations that can be attributed to the Minamata Convention?

iii. To what extent are existing measures under the Minamata Convention meeting the objective of protecting human health and the environment from mercury?

To answer these questions, biomonitoring will help with: (1) establishing linkages between Hg source types and biota, (2) describing spatial gradients, and (3) tracking temporal trends.

#### Establishing linkages between Hg source types and biota

A promising new method for linking major Hg source types and Hg found in biota is the use of variations in stable Hg isotope ratios (e.g., Blum et al. 2014; Kwon et al. 2014, 2020; Li et al. 2016, 2022; Renedo et al. 2020; Manceau et al. 2021c). Mercury has seven stable isotopes and undergoes mass fractionation following many different patterns of isotope ratio variation during chemical reactions. The most widely used isotopic ‘signals’ of sources and chemical processes are: mass dependent fractionation (MDF), odd isotope mass independent fractionation (odd-MIF), and even isotope mass independent fractionation (even-MIF). The magnitude of the three ‘signals’ as well as the ratios between them can be combined to distinguish Hg sources and chemical processes in the environment (Blum and Johnson 2017).

By measuring the isotopic ratios of Hg in environmental samples, certain linkages can be established, and others can be eliminated in investigations of Hg sources (Le Croizier et al. 2020). Conclusive source receptor-relationships are challenging for Hg, even when using isotopes, in part due to the extensive re-emission from the earth surface back to the atmosphere after initial deposition (Outridge et al. 2018). So, while the method is often not definitive by itself, by combining isotopic data with other information based on Hg concentrations and chemical speciation, the evidence considered together can be conclusive. There are many examples in the literature where Hg isotopes have been used to separate the origin of Hg from global gaseous Hg background, global precipitation, coal burning facilities, chlor-alkali facilities, gold mining, and other industrial sources – particularly at local scales (Obrist et al. 2018).

Several studies have shown that local atmospheric sources of Hg from industrial output can be identified in precipitation and in gaseous Hg because they contrast in isotopic composition with globally well-mixed atmospheric reservoirs (Sherman et al. 2012). Similarly, industrial inputs of Hg to rivers, lakes, and marine coastal areas can often be distinguished from natural background Hg and atmospherically deposited Hg based on isotopic composition (Donovan et al. 2014). Mercury isotopes have also been used as an indicator of Hg methylation and demethylation rates and hotspots within ecosystems, and more broadly as a tool for understanding Hg biogeochemistry (Donovan et al. 2016). In situations where at least two isotopically distinct sources of Hg are present, Hg isotopes have also been used to trace the source of Hg in biota and humans (Sherman and Blum 2013).

To link Hg sources with changing Hg concentrations in biota is complicated due to the complex post-emission processing of Hg (i.e., between emission/release and uptake in the food chain; Li et al. 2022). However, efforts have been made to make the link evaluating Hg burdens in different biotic media. For example, in the Fennoscandian fish Hg database more than 3000 lakes were classified per dominant Hg pollution source based on expert judgment, including (1) lakes with no local Hg pollution sources, implying that atmospheric deposition of Hg is the dominating pollution source, and (2) lakes with known local industry point source(s) (Braaten et al. 2019). The data indicated that for the point-source lakes (2), the temporal trends showed a significant long-term decreasing trend between 1965 and 2015. However, since 1995, the temporal trends were not decreasing, indicating that most of the change in concentrations happened earlier. The authors argue that, in Fennoscandia, a peak in industry emissions and releases occurred during the 1950s and 60 s and since the 1980s local emissions and releases in Fennoscandia have been reduced significantly. This is more recently confirmed in an evaluation of the effectiveness of Norwegian Hg regulations and policies (i.e., the National Mercury Assessment for Norway; Braaten et al. 2022) where the official governmental total emissions of Hg to the atmosphere and releases to soil and water are documented. The reasons for the decline in discharge and emissions in Scandinavia are, in addition to regional and national control legislation, improved technology and reduction of polluting industrial production.

#### Describing spatial gradients

The availability of MeHg to high trophic level organisms can vary widely in relation to environmental conditions. Some ecosystems are more sensitive to inorganic Hg input than others (Driscoll et al. 2007; Eagles-Smith et al. 2016a, 2018; Branfireun et al. 2020) and it is these areas where biological MeHg hotspots (or ecosystem sensitive areas) can form and are especially pronounced in higher trophic-level organisms (Evers et al. 2007). For terrestrial ecosystems, such areas are generally associated with wetlands and other temporally wetted habitats and can be particularly pronounced in ecosystems with water chemistry variables such as low pH, moderate to high dissolved organic carbon concentrations, low to moderate primary productivity, or availability of sulfur (Bishop et al. 2020). In particular, fluctuating water levels can have an important contribution in generating higher methylation rates and increases in MeHg bioavailability (Evers et al. 2007; Willacker et al. 2016); and may happen at daily (e.g., tidal), monthly (e.g., artificial reservoirs and pools), or seasonal (river floodplains and dry tropical areas flooded during the wet season) timeframes, as well as in areas where water levels are managed (e.g., rice agriculture).

Therefore, determining which areas may have elevated MeHg availability requires consideration of other environmental factors in addition to the deposition or release of inorganic Hg into the environment. Globally, models can now identify the sensitivity of ecosystems and therefore areas of greatest concern (Evers et al. 2023). Because of an understanding of environmental factors that drive methylation rates in temperate ecosystems in North America and Europe, as well as a history of abiotic and biotic Hg data collection there are many good examples that quantitatively assess the dynamics between atmospheric deposition of Hg and the ecological response in lakes.

For example, Kejimkujik National Park (Nova Scotia, Canada) experiences relatively low precipitation-weighted mean concentrations and deposition of total Hg (<5 ng/L and <7.5 µg/m^2^/y, respectively; Dastoor and Larocque 2004; Dastoor et al. 2015), yet biotic MeHg accumulation is some of the highest in North America (e.g., 0.30 and 3.0 µg/g, ww in fish muscle and bird blood, respectively; Evers et al. 1998; Burgess and Hobson 2006; Burgess and Meyer 2008; Wyn et al. 2009, 2010). Fennoscandia is another example where the contemporary global “background” levels of atmospheric Hg deposition support Hg concentrations in fish that often exceed healthy advisory levels, even after half a century of decline in the measured fish Hg levels of the region (Braaten et al. 2017, 2019). Most lakes and catchments in the area are sensitive to inorganic Hg input and have high methylation potential and MeHg bioavailability because of important habitat characteristics including a combination of low pH, high dissolved organic carbon, high percentage of hydrologically-connected wetlands, low primary productivity, and in some cases catchment disturbance such as forest management or beaver activity (Bishop et al. 2009; de Wit et al. 2014; Eklöf et al. 2018; Negrazis et al. 2022). Ultimately, the identification of biological MeHg hotspots for freshwater and terrestrial eocsystems can be improved via better standardization of existing biotic data (Evers et al. 2011b; Ackerman et al. 2016; Eagles-Smith et al. 2016a) to inform modeling of ecosystem sensitivity at multiple spatial scales (Evers et al. 2023).

In marine regions, spatial patterns in biological MeHg concentrations are less resolved but will be facilitated by the development of a global biotic database of Hg concentrations in marine species and supporting modeling efforts to help explain observed spatial patterns. Differences in MeHg concentrations across ocean basins are clear from the literature. For example, Médieu et al. (2021) reported a five-fold spatial gradient in total Hg concentrations in albacore tuna across the North Pacific Ocean. This trend is driven by local anthropogenic Hg release along the Asia coast – where total Hg concentrations in albacore tuna are highest. Globally, the highest concentrations of MeHg in seawater have been reported in some regions of the Southern Ocean, which also have elevated concentrations of MeHg in some food webs (Cossa et al. 2011). Considerable spatial variability in seawater MeHg concentrations has been reported among other ocean basins, with highest levels in subsurface waters of the most biologically productive areas (Cossa et al. 2009; Sunderland et al. 2009; Bowman et al. 2014, 2016; Munson et al. 2015; Kim et al. 2017). The Arctic appears to have higher concentrations of MeHg in near-surface seawater, which may reflect unique microbial activity resulting from the combination of stratification, freshwater discharges, and ice cover (Lehnherr et al. 2011; Heimbürger et al. 2015; Schartup et al. 2015a). Much work remains to gather more data from data-poor basins (see Tables 5 and 6), and link MeHg production areas in the ocean to tissue concentrations in marine biota.

#### Tracking temporal trends

Models simulating the deposition of Hg from anthropogenic emissions at global scales (using several anthropogenic emissions scenarios) indicate a best scenario of a decrease of up to 50% in the Northern Hemisphere and up to 35% in the Southern Hemisphere by 2035 relative to 2010 (Pacyna et al. 2016). Although tracking Hg emissions, deposition, and releases are important tools for understanding patterns of environmental Hg loads (Sundseth et al. 2017), the relationship between modeled (or measured) deposition and MeHg concentrations in biota is poorly understood in both freshwaters and oceans, and usually when measured divergence in Hg trends in air and biota is likely (Wang et al. 2019a). Reasons for this divergence are often unexplained but can sometimes be linked to shifts in trophic structure and dietary preferences initiated by invasive species (Lepak et al. 2019). Observations of long-term trends are critical for improving the understanding of the linkages and can be viewed through fish, birds, and marine mammal studies using data within the GBMS database.

Trends in inorganic Hg concentrations are thought to differ among ocean basins because anthropogenic emissions have strongly declined in North America and Europe, leading to large declines in atmospheric concentrations, especially in the Atlantic Ocean (Zhang et al. 2016). Lee and Fisher (2016) postulated that this may also explain observed declines in Atlantic bluefin tuna MeHg concentrations between 2004 and 2012 in the North Atlantic Ocean – which are supported in measured Hg concentrations in blue marlin (*Makaira nigricans*) (Rudershausen et al. 2023). In a Norwegian study, Braaten et al. (2020), argued for a link between declines in Hg in fish in pristine lakes since the 1970s and reduced sulfate deposition in northern Europe. Mercury trends in biota (downward) and sediment (upward) indicated a disconnect between lake Hg loading and food web Hg bioaccumulation. The authors suggested that reduced sulfate deposition constrains substrate availability for sulfate-reducing methylating bacteria (causing reduced food web MeHg exposure despite increased Hg loading to the lake).

The relationship of changing fish MeHg concentrations in different ocean basins is germane to a better understanding of the geographic origins of Hg in seafood by country or region. For example, in the U.S., 45% of population-wide MeHg exposure originates from open oceans (particularly the Pacific Ocean), 37% from domestic coastal ecosystems, and 18% from aquaculture and freshwater fisheries (Sunderland et al. 2018). While, in the North Pacific Ocean, both atmospheric emissions and freshwater discharges of Hg have been growing on the Asian continent leading to increased Hg levels (Amos et al. 2013, 2014; Streets et al. 2009; Sunderland et al. 2009; Zhang et al. 2015). Most recent data indicate the rate of growth in Hg emissions has been slowed by widespread implementation of emissions controls on new coal-fired utilities (Giang et al. 2015; Streets et al. 2017, 2019). While temporal data on fisheries in the North Pacific are more limited some researchers have suggested that there is evidence for increases in tuna MeHg concentrations over recent decades (Drevnick et al. 2015), which is further supported by additional analysis of bigeye tuna for the same area (Drevnick and Brooks 2017).

In freshwaters, the regional fish observations across the northeastern United States (e.g., Millard et al. 2020; Richter and Skinner 2020) and Fennoscandia (Braaten et al. 2017) stand out as examples of both the potential and challenges of long-term monitoring of biota. In the northeastern United States, records are decades long for fish. The patterns here are complex, despite documented decreases in atmospheric deposition and regional Hg emissions to the atmosphere (Evers et al. 2020). While fish of some species have shown long-term declines, gamefish (e.g., largemouth bass) have had stable Hg concentrations (Richter and Skinner 2020). In the Adirondack, New York region within the United States, comparative surveys of lakes sampled a decade apart in the 2000s showed Hg increases in fish (Millard et al. 2020). The wide variation of responses seen in the biota of the northeastern United States reveal the role of environmental factors on Hg biomagnification, including climate and dissolved organic carbon concentrations in surface waters.

In Fennoscandia, the half century of records dating back to the mid-1960s is composed of fish collected from different lakes, different species, and different sampling methods. Nonetheless, the large number of fish and the decades of Hg data make it possible to reasonably associate both regional declines in atmospheric deposition and reductions in local pollution sources have led to reduced levels of Hg in fish (Braaten et al. 2019). Such large, long-term data and associated analyses are a resource for both designing new, long-term programs and the interpretation of existing datasets compiled from different sources to achieve better spatial and temporal coverage.

While freshwater fish Hg data regularly exist for the North American and European continents because of intensive field sampling efforts linked to human exposure concerns since the 1960s (Johnels et al. 1967), birds are also useful bioindicators of those and other continents and biomes. Multi-decadal Hg biomonitoring in birds is well-established in North American lakes using blood, feathers and eggs in breeding common loons (Evers et al. 1998, 2003; Meyer et al. 2011; Schoch et al. 2020), in eggs for herring gulls for the Great Lakes (Blukacz-Richards et al. 2017), and in eggs for seabirds in the Arctic (Braune et al. 2016; Bianchini et al. 2022) and Atlantic Canada for the Leach’s storm petrel (Calvert et al. 2024).

Through the use of feathers from museum specimens, birds can also provide a temporal profile that commonly exceeds one century. Recent studies that have combined retrospective Hg analyses of museum feathers with their contemporary counterparts from field samples are particularly important. For example, from the tundra of Alaska’s North Slope feather Hg concentrations from 1845 to 2012 indicated a doubling of Hg body burdens for the yellow-billed loon with projections of a four-fold increase by 2050 (Evers et al. 2014). Another retrospective study across Canada and northeastern United States found feather Hg concentrations from 1871 to 2015 from the rusty blackbird (*Euphagus carolinus*) to have substantially increased by 10-fold or more (Perkins et al. 2020).

To investigate changes in Hg over the past century in the Arctic landscape, Dietz et al. (2011) analyzed Hg in polar bear hair from northwest Greenland during 1892–2008. Mercury concentrations showed yearly significant increases of nearly 2% over that nearly 120-year time period. No change in trophic levels over this period was detected from stable isotopes, so changes in feeding patterns do not explain the change in Hg exposure. These trends were in accordance with an earlier review (Dietz et al. 2009). The latter study examined the literature concerning the long-term changes of Hg in humans and selected Arctic marine mammals and birds of prey since preindustrial times (i.e., before 1800 A.D.), to determine the anthropogenic contribution to present-day Hg concentrations and the historical timing of any changes. The authors calculated historical trends in Hg concentration in hard tissues of various Arctic biota. They found that “on average, 92% of the present-day Hg in Arctic wildlife is likely to be of anthropogenic origin” (AMAP 2011), while studies across tissues and species from the circumpolar Arctic generally did not demonstrate a consistent trend during the last 30 years or so (Rigét et al. 2011; Morris et al. 2022a, b). This is related to varied responses in different tissues (e.g., muscle vs. liver tissues) and that biota Hg concentrations are influenced by many factors, including changes in the food web (Morris et al. 2022a).

### Framework for global mercury biomonitoring

To develop a sustainable and long-term global biomonitoring framework that could link existing biotic Hg data and biomonitoring programs with objectives of the Minamata Convention, several criteria could be considered, including: (1) stabilizing and expanding existing monitoring programs, (2) identifying areas that have regional data gaps so new programs can be purposely launched that define relevant sample sizes necessary for understanding spatial gradients that incorporate ecosystem sensitivity (Evers et al. 2011b) and temporal trends (Rigét et al. 2011; Morris et al. 2022a, b), and (3) identifying an existing queryable global environmental database platform to serve as a standardized and interpretive source of biota Hg information (e.g., United Nations Environment Program’s World Environment Situation Room; www.wesr.unep.org).

Moreover, international collaboration and coordination among national projects (emphasizing ratified countries) could create harmonized regional approaches and would integrate biomonitoring activities into an interdisciplinary framework to assess ecological and human health risks. Using the example of successful programs such as the Northern Contaminants Program (NCP) in Canada for the development of other national programs may also aid in building capacity of Indigenous Peoples in monitoring and research activities, which can lead to more robust monitoring and a more comprehensive understanding on Hg levels in the ecosystem and their possible drivers in addition to helping guide policy development (Houde et al. 2022). By developing a hierarchical framework, data compilation would be easier and regional and temporal trends could be assessed. Based on an information document for the Conference of Parties for their fourth meeting of the Minamata Convention on Mercury (UNEP 2022), guidance on monitoring of Hg and Hg compounds to support evaluation of the effectiveness can be grouped to achieve six objectives: (1) Estimation of contemporary Hg concentrations for areas without (i.e., background sites) or with (i.e., affected sites) local anthropogenic sources; (2) Identification of temporal trends; (3) Characterization of spatial patterns; (4) Estimation of source attribution; (5) Estimation of exposure and adverse impacts, and; (6) Quantification of key environmental processes to improve our understanding of cause-effect relationships.

Based on the knowledge of existing biotic Hg exposure, data availability is generally sufficient for tracking temporal trends and spatial gradients for all major taxa as bioindicators for both human health and the environment in the Arctic (AMAP 2021), as well as for fish in Canada and Europe (covering parts of the boreal and temperate mixed forests). There are some long-term Hg monitoring programs that include birds in North America (e.g., loons [*Gavia* spp] in temperate lakes of the United States and Canada; Scheuhammer et al. 2016; Evers et al. 1998, 2005, 2011a, 2020) and in the Arctic and subarctic (Fort et al. 2017). Retrospective MeHg analyses of museum bird feathers (Frederick et al. 2004; Head et al. 2011; Evers et al. 2014; Perkins et al. 2020) and mammal fur, whiskers, and baleen (Dietz et al. 2011) are a promising approach for expanding options for examining temporal trends in many regions of the world. Therefore, in the interest of using comparable data for relevant terrestrial biomes and associated aquatic areas, based on existing data (See Tables 5 and 6), we suggest that a matrix of available data and museum specimens can respond sufficiently to initial overarching questions related to temporal trends and spatial gradients.

A generalized assessment of global Hg data availability describes areas where existing data gaps are most notable - such as within the tropical rainforest biome and associated marine areas (Table 7). These areas are most problematic when coupled with Hg releases from artisanal small-scale mining activities and other major Hg source types. Information for marine mammals is generally missing as well, except for the Arctic Ocean. The preferred choice of trophic level 4 or higher bioindicators by biome and general ecosystem type (i.e., land, freshwater, marine) is influenced by objective (e.g., linking human Hg exposure, source types, understanding spatial gradients and tracking temporal trends) and several other factors (e.g., practicality, sustainability, comparability, and cost effectiveness are all factors to consider for Hg monitoring in biota).

**Table 7:** Generalized assessment of global Hg data availability at poor (Data gap), good (X) and excellent (XX) levels for trophic level 4 bioindicators within major biomes and associated marine areas for both ecological and human health bioindicators

Terrestrial biomes and associated marine areas	Ecological health bioindicators			Human and ecological health bioindicators		
	**Freshwater birds**	**Marine birds**	**Marine mammals**	**Freshwater fish**	**Marine fish**	**Marine mammals**
Arctic Tundra and Arctic Ocean	XX	XX	XX	XX	XX	XX
Boreal Forest-Taiga and N. Pacific and Atlantic Ocean	X	X	Data gap	XX	X	Data gap
Temperate Mixed Forest and Pacific and Atlantic Ocean	XX	X	Data gap	XX	X	Data gap
Tropical Rainforest and S. Pacific and Atlantic and Indian Ocean	Data gap	Data gap	Data gap	Data gap	Data gap	Data gap

The data availability category “excellent levels” indicates information is available for tracking both temporal trends and spatial gradients. This assessment is based on quantified findings in Table 6

One way to make a relatively standard comparison of global Hg exposure in high trophic level fish species is to assess the percentage that may exceed human health standards. Based on the GBMS database, the percentage of fish, seabirds (based on eggs), and marine mammals that average over or include individuals that exceed 0.46 µg/g, ww of total Hg in edible tissue can be determined for taxonomic groups of concern to people (Table 8). Notably, over half (or nearly half) of the species, genera or families include individuals that exceed 0.46 µg/g, ww in tuna, billfish, sharks, marine fish in the Mediterranean and Caribbean Seas, and freshwater fish in North and South America, and Europe, as well as marine mammals. Africa has the greatest number of fish families that are considered safe to consume by humans (for at least one meal per week). A global analysis of freshwater fish for muscle Hg concentrations within the GBMS database (n = 312,335) indicates that 45% of the 131 families include individuals that exceed 0.46 µg/g, ww.

**Table 8:** Percentage of species or families that include individuals with muscle tissue Hg concentrations exceeding the recently updated. USEPA – USFDA human health threshold of 0.46 µg/g, ww (“choices to avoid”)

Taxa group of interest to human health	Taxonomic unit	Number	Percentage exceeding risk
Tuna	Species (commercial)	6 of 9	67%
Billfish	Species	5 of 7	71%
Sharks	Genera	21 of 24	88%
Marine Fish – Mediterranean Sea	Families	24 of 36	67%
Marine Fish – Caribbean Sea	Families	25 of 39	64%
Freshwater Fish – African	Families	3 of 16	19%
Freshwater Fish – South America	Families	17 of 36	47%
Freshwater Fish – Asia	Families	21 of 31	39%
Freshwater, estuarine, marine fish - Australia	Families	7 of 18	38%
Freshwater Fish – North America/Europe	Species	12 of 25	48%
Seabirds – Arctic and subarctic	Species	6 of 20	30%
Marine Mammals – toothed whales	Species	38 of 38	100%

Percentages are based on data from the graphs presented within this paper

A further breakdown of global Hg exposure as indicated by biota can be viewed by biome. In the Arctic, standard bioindicators have been selected by AMAP to monitor Hg for human health and the environment and represent a long-term existing dataset and confidence for future coverage (AMAP 2021). In the taiga and boreal areas of the Northern Hemisphere comparable Hg data are available (because of relatively similar game fish species) in Canada, the United States and Fennoscandia. The practicality and sustainability of Canada’s NCP and those directed by the other country’s respective governments makes the operation of standardized Hg monitoring programs cost-effective (Depew et al. 2013; Gandhi et al. 2014). The major exception for these northern biomes is Russia (Morris et al. 2022a).

For temperate biomes in the western hemisphere, existing (or recent) efforts are primarily in place in parts of the United States (e.g., state efforts such as in New York; Millard et al. 2020) and parts of Europe (Braaten et al. 2019) for freshwater ecosystems and some marine areas – although they rarely reflect long-term datasets and are generally not standardized across states, provinces, and countries. However, regional efforts in the Great Lakes and national efforts in United States rivers have provided standardized abiotic data over time (Wathen et al. 2015a, 2015b; Grieb et al. 2020). Southern hemisphere Hg biomonitoring efforts in temperate biomes are not as strong as the northern hemisphere and if added could contribute to the knowledge of hemispheric Hg cycling (Chen and Evers 2023).

In tropical and subtropical areas, few Hg monitoring efforts and datasets are in place. Environmental Hg-related research has been significant in some countries, such as Brazil and China, but are not as robust for Hg biomonitoring as in temperate areas. The practicality, sustainability and comparability are also all challenging because of limited infrastructure and history of monitoring activities. In addition, ecosystems and habitats that are susceptible to creating elevated levels of MeHg availability that may or may not be associated with ASGM contamination include reservoirs (Ouédraogo and Amyot 2013), estuaries (Diop and Amara 2016), and large wetlands (Daso et al. 2015). River deltas and estuaries are especially of interest as they have high methylating abilities and fisheries resources that are important to local communities. There are very few data from these ecosystems along the African coast, which are among many examples where more investigations could help to fill the extensive data gaps on the African continent (see Table 6). In particular, human and ecological exposure to MeHg is thought to be generally lower in African versus North American and European temperate lakes (i.e., the “tropical African mercury anomaly”; Black et al. 2011). Since MeHg biomagnification rates appear to be similar in African lakes to temperate and Arctic lakes (Kidd et al. 2003), the selection of bioindicators needs to be cautiously made in geographic areas considered to have high sensitivity to Hg input (Evers et al. 2023). Ultimately, tropical ecosystems are especially limited with Hg biomonitoring programs.

Across ocean basins (outside of the Arctic and Antarctic Oceans), commercial fisheries for tuna and billfish provide a platform for long-term, sustainable, and cost-effective monitoring of Hg based on existing and regular capture opportunities that can be coupled with sampling (Esposito et al. 2018; Médieu et al. 2023). Nearshore fish monitoring signifies Hg concentrations that may differ from those in offshore more pelagic fish, especially when considering the complexity and variable processes related to offshore Hg deposition, methylation, bioavailability, and biomagnification (Médieu et al. 2021). In response, high trophic level species such as barracuda, snapper and grouper are important bioindicators for evaluating nearshore MeHg availability to fish (Christian et al. 2024).

Mercury biomonitoring will need to incorporate potential confounding impacts from global climate change (Pinkney et al. 2015; Sundseth et al. 2017; Schartup et al. 2019; Bishop et al. 2020; Sonke et al. 2023), which is supported by findings in marine ecosystems (McKinney et al. 2015; Sundseth et al. 2015; Wang et al. 2023a, 2023b; Bargagli and Rota 2024), Arctic ecosystems (McKinney et al. 2015; Sundseth et al. 2015; Chételat et al. 2022; McKinney et al. 2022; Grunst et al. 2023), subarctic and temperate lakes (Chen et al. 2018), temperate estuaries (Jonsson et al. 2017; Willacker et al. 2017), and terrestrial temperate (Eagles-Smith et al. 2018) and tropical ecosystems (Yang et al. 2023). Specific effects of global climate change that impact MeHg availability include enhanced air-seawater exchange, melting of polar ice caps and glaciers, increased thawing of permafrost, and changes in estuarine sulfur biogeochemistry. However, how these landscape processes relate to changes in biotic Hg exposure is relatively unknown. Sunderland et al. (2018) demonstrated that global climate change is altering fish harvest MeHg exposures in species such as cod and pollock that are sensitive to climate-driven warming of seawater.

Iterative efforts to link realistic and applied biomonitoring efforts at local levels with regional science-policy groups aimed at assisting the Conference of Parties of the Minamata Convention may ultimately help keep pace with the many emerging scientific findings that may fill existing information gaps. As the overall understanding of source types and their ecosystem linkages, spatial gradients, and temporal trends, and the interest of using bioindicators for human exposure and ecological health that reasonably reflect terrestrial, freshwater, and marine environments, two overarching global biotic Hg monitoring approaches have been identified for continents and oceans; these approaches are partly described below and are more fully detailed in Evers and Sunderland (2019).

#### Continental framework for integrated mercury monitoring

**Fig. 22:**
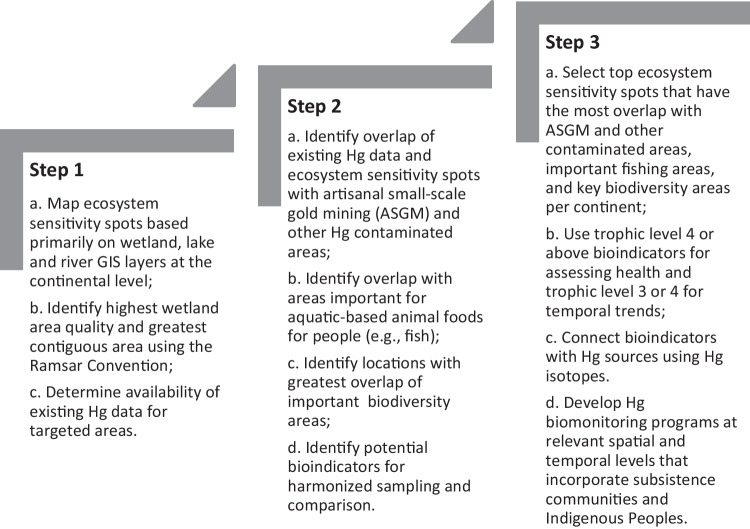
Stepwise components for developing a conceptual continental approach using biota for Hg monitoring (see Evers and Sunderland 2019)

To identify the best locations for global Hg monitoring requires multiple defined steps (Fig. 22). An initial step is to understand the complexities of a landscape and its ability to methylate Hg and make it available in the foodweb. Net mercury methylation rates are generally high in wetlands – particularly in estuarine wetlands such as mangroves, peatlands, and lake or rivers with shoreline wetlands especially those associated with fluctuating water levels. Forested areas are also an important factor for increasing dry deposition rates of atmospheric Hg in temperate (Driscoll et al. 2007; Obrist et al. 2018) and tropical (Gerson et al. 2022) ecosystems, while agricultural areas that introduce large amounts of phosphorus and nitrogen to freshwater systems tend to dampen methylation levels by promoting biodilution (Chen et al. 2008; Lavoie et al. 2013). As many of the most important wetland areas in the world are identified and protected through the Ramsar Convention (https://www.ramsar.org/), their 2,341 locations covering 252,489,973 ha, along with ASGM activities (e.g., Steckling et al. 2017), FAO data, and Red List species as identified through the International Union for Conservation of Nature (https://www.iucnredlist.org/), can be feasibly combined with the GBMS database to further advance analytical assessments. For example, the identification and potential overlap of ecosystem sensitivity spots with priority lakes, rivers and wetlands may help prioritize areas of greatest concern for protecting human health and the environment. Similarly, summarizing information by watershed has proven to be an important base area for mapping, providing additional hierarchical structure whereby the choice of the most appropriate scale for analysis (i.e., choice of hydrologic unit codes [HUC], and land-use impacts) will depend on the specific objectives being assessed (Evers et al. 2023).

#### Oceanic framework for integrated mercury monitoring

**Fig. 23:**
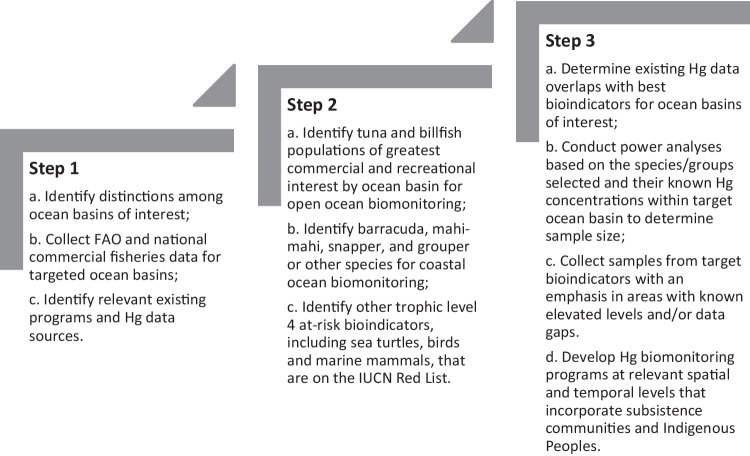
Stepwise components for developing a conceptual oceanic approach using biota for Hg monitoring (see Evers and Sunderland 2019)

The approach for monitoring Hg in oceanic areas greatly differs from the continental approach (Fig. 23). The cycling and movement of Hg in the world’s oceans varies by hemisphere, basin, ocean depth, and juxtaposition with the continental land masses and associated river deltas. Therefore, Hg concentrations in fish, birds, and marine mammals vary significantly. For example, bluefin tuna (representing three sibling species – the Atlantic, Pacific, and Southern) have average Hg concentrations in their muscle tissue across six ocean regions that may vary three-fold (Fig. 2). Reasons for this variation differ and need to be accounted for when globally monitoring Hg in oceanic areas.

Based on the GBMS database, the species of highest Hg concern with the greatest interest for human consumption are the larger tuna species and swordfish. The Hg concentrations in tuna vary greatly by species (Fig. 2) because of their growth rates, ultimate size, age, trophic level, and ocean basin and therefore species selection is important. Médieu et al. (2023) provide a template for monitoring Hg in tuna, to standardize data collection and reporting, and also suggest other environmental variables that could be integrated into monitoring to aid in interpretation of spatial and temporal trends. Coastal fish biomonitoring would generally include different species – for the Caribbean a new Hg biomonitoring network has identified four key species (barracuda, mahi-mahi, and various snapper and grouper species (Christian et al. 2024). While commercial harvest of some of the highest-trophic level fish is important for global Hg biomonitoring, perhaps some of the most vulnerable populations are Indigenous Peoples and subsistence communities who depend on a broad variety of biota for their local, traditional diet – which includes many species with established highly elevated MeHg body burdens in the Arctic and subarctic (Basu et al. 2018, 2023), within tropical systems (Salazar-Camacho et al. 2020), and across oceanic islands (Sabino et al. 2022).

## Conclusions

Efforts to assemble disparate but related biotic Hg data into standardized and comparable databases are essential for understanding the patterns and trends of Hg exposure, and for informing critical policies designed to lower Hg risks and impacts to ecosystems and people. Building from a history of successful regional efforts in North America, we describe the use of existing monitoring programs and a first effort to assemble a standardized global database of published, peer-reviewed Hg concentrations in biotic tissue - called the Global Biotic Mercury Synthesis (GBMS). We use this database to identify critical knowledge gaps and describe adoption of specific quantitative and replicable approaches to create harmonized biomonitoring efforts that can be developed and made available to countries. We provide examples of how to standardize efforts to document where, when, how, and what to monitor for tracking environmental Hg loads, their changes over time, and potential impacts on human and ecological health.

To illustrate the value of GBMS data to environmental policies, we present new syntheses of global Hg data in relation to Minamata Convention objectives. Our findings demonstrate that while there are a few large biological Hg datasets, they generally do not provide the ability to determine changes in biotic Hg exposure at regional or global scales over decadal periods (with the notable exceptions of AMAP, the Northern Contaminants Program in Canada, and the Fennoscandian fish database) in response to the obligations of the Minamata Convention (Evers et al. 2016; Potera 2019; Rosendal et al. 2020). Robust statistical approaches are critical for confidently tracking biotic Hg concentrations in the many different biomes around the world, and controlling for the effects of other factors, such as global climate change, altered foraging habitat, changes in primary productivity, and changing growth rates that can drive shifts in biotic MeHg concentrations that are not due to altered anthropogenic loading of Hg to the ecosystem. One factor in particular, global climate change, will alter future MeHg concentrations in biota in all biomes and ocean basins. Specific effects of global climate change include enhanced air-seawater exchange, melting of polar ice caps and glacier ice sheets, increased thawing of permafrost and changes in estuarine sulfur biogeochemistry – but how these landscape processes relate to changes in biotic Hg exposure is relatively unknown (Wang et al. 2019a).

Iterative efforts to link realistic and applied biomonitoring efforts at local levels with science-policy groups aimed at assisting the Conference of Parties of the Minamata Convention will ultimately help keep pace with the many emerging scientific findings that may fill existing information gaps that are key for local landscape management as well as global policymaking. Ultimately, the careful selection and use of bioindicators that closely match provisions of the Minamata Convention (e.g., linkages to Hg sources, spatial gradients, and temporal trends) can be a cost-effective and time-efficient way to track human and ecological health of anthropogenic loading of Hg into the air and onto the water and landscape at a global level (Evers et al. 2016; Evers and Sunderland 2019). As described, the methods for biomonitoring and the interpretation of the tissues sampled are generally well-established for many target taxa. The extensive knowledge of Hg exposure in a wide range of fish and wildlife that are available in existing monitoring programs and research efforts are described in the peer-reviewed literature, and now in the GBMS database. This therefore provides a platform for informed selection of the appropriate taxa within specific biomes or waterbodies. For example, a synthesis of the compiled global Hg datasets that represent Hg concentrations of biota ingested by people (i.e., freshwater and marine fish, seabirds, and marine mammals) found 45% of the 131 families of representative organisms include individuals that exceed the 0.46 µg/g, ww newly updated benchmark identified by the United States government as food “choices to avoid” (n = 312,335 individuals; Table 8).

Biomonitoring should build from existing programs, which are generally found within developed countries at local, national, and sometimes regional levels. Global pilot projects based on existing networks with local organizations and governmental agencies have been tested for fish (Buck et al. 2019) and humans (Trasande et al. 2016), and regional biomonitoring approaches in temperate and tropical marine ecosystems are described (Evers et al. 2008; Christian et al. 2024). Generating a more coordinated global approach that provides best practice examples, can connect existing biomonitoring programs and identify the ecosystem, taxa, or geographic gaps that are both needed and feasible. Research and monitoring efforts that work in an equitable and ethical partnership with Indigenous Peoples and utilize Indigenous Knowledge have been found to be particularly successful and can also be used as examples (Houde et al. 2022).

In order to better understand and reduce the impact of Hg on people and the environment, additional effort is needed to bridge information and knowledge gaps more effectively. There are many landscape, ecological, and demographic factors that influence MeHg generation and bioavailability – many of which are known and can be used for scaling models. Other factors that affect spatial gradients of biotic MeHg exposure still need further investigations (e.g., ASGM and climate change). Once global needs and interests of the Minamata Convention are determined by the Conference of Parties, we suggest that it is feasible to generate cost-efficient and reliable biomonitoring approaches at geographic scales of interest that can be integrated with existing local and regional Hg biomonitoring networks. Invariably, a commitment to long-term standardized regional biomonitoring approaches is needed - as proven by a 50+ year global tuna Hg assessment that identified multiple limitations generated by high inter-annual variability among species and geographical scale (Médieu et al. 2024).

Lastly, there is an urgency to monitor and assess the influence of MeHg on biota because of the potential adverse impacts to biological diversity (e.g., at ASGM sites: Palacios-Torres et al. 2018; Dossou et al. 2024) during a time when global stressors are causing long-term and significant declines (Leclère et al. 2020; Eddy et al. 2021). Recent evidence demonstrates that the multifaceted effects of anthropogenic chemicals and other pollutants such as Hg in the environment are posing a growing threat to biodiversity (Sigmund et al. 2023) and that there is justification in targeting a wider scope of environmental contaminants within strategies and actions associated with the post-2020 global biodiversity framework of the Convention on Biological Diversity (Sigmund et al. 2022).

### Supplementary information

GBMS References FINAL FINAL SUBMISSION4

## Data Availability

All data are available from Biodiversity Research Institute (www.briwildlife.org).
